# Efficacy and safety of prolyl hydroxylase inhibitors for anemia in chronic kidney disease: a network meta-analysis

**DOI:** 10.1080/0886022X.2026.2616572

**Published:** 2026-02-03

**Authors:** Yuanchen Niu, Tenghua Wang, Yaoxuan Zhan, Haimei Xu, Chen Li, Zhiqin Hu, Jin He, Yongmei Li, Yi Fang

**Affiliations:** a Guangdong Engineering Research Center of Early Clinical Trials of Biotechnology Drugs, The Fifth Affiliated Hospital, Guangzhou Medical University, Guangzhou, Guangdong, China; b Clinical Trial Institution Research Ward, Peking University People’s Hospital, Beijing, China

**Keywords:** Prolyl hydroxylase inhibitors, renal anemia, chronic kidney disease, dialysis, network meta-analysis

## Abstract

This Bayesian network meta-analysis evaluated the efficacy and safety of six prolyl hydroxylase inhibitors (PHIs) versus erythropoiesis-stimulating agents (ESAs) or placebo for chronic kidney disease (CKD) anemia. Electronic databases were searched through 1 May 2025. The prespecified primary outcome was change in hemoglobin, while secondary outcomes included iron metabolism indices (serum iron, transferrin saturation [TSAT], ferritin, hepcidin) and overall composite adverse events (AEs). Surface under the cumulative ranking curve (SUCRA) was used for ranking. Forty-six randomized controlled trials with 32,305 patients were included. In non-dialysis CKD (ND-CKD), roxadustat yielded the greatest hemoglobin rise (MD = 1.21 g/dL, 95% CI: 0.72–1.71; SUCRA = 88.6%) versus placebo and ESA. In dialysis CKD (D-CKD), roxadustat again ranked the highest (MD = 1.78 g/dL, 95% CI: 1.32–2.24; SUCRA = 86.2%) versus ESA and other PHIs. For TSAT, ESA ranked best in ND-CKD (MD = 5.24%, 95% CI: 1.16–9.32), while daprodustat led in D-CKD (MD = 3.07%, 95% CI: 0.22–5.91). Roxadustat improved serum iron in D-CKD (MD = 6.19 μg/dL, 95% CI: 2.81–9.58), and vadadustat and roxadustat reduced hepcidin in ND-CKD. Consistency assessment revealed no statistically significant heterogeneity/inconsistency between direct/indirect comparisons, supporting the robustness of the results. Regarding safety, ESA had the lowest AE risk in ND-CKD (OR = 0.85, 95% CI: 0.74–0.98), while roxadustat ranked lowest (SUCRA = 18.4%). Roxadustat demonstrated the strongest efficacy in hemoglobin improvement but higher AE incidence in ND-CKD, whereas ESA and daprodustat showed safety and iron metabolism benefits, supporting individualized therapy for renal anemia.**Registration number:** PROSPERO (CRD420251066181)

## 1. Introduction

Anemia is a common and clinically important complication of chronic kidney disease (CKD) caused primarily by reduced erythropoietin (EPO) production and iron dysregulation in renal failure [1,2]. By Kidney Disease: Improving Global Outcomes (KDIGO) criteria (2012), anemia in CKD is defined as hemoglobin <130 g/L in men or <120 g/L in women [3]. The prevalence of renal anemia rises as CKD progresses; roughly 60% of patients with non-dialysis-dependent CKD (ND-CKD) and over 90% of those on dialysis-dependent CKD (D-CKD) are anemic [4]. Globally, the burden of CKD-related anemia is massive: a recent analysis estimated 63.8 million cases in 2021 (up 96% since 1990) [5]. Furthermore, recent global forecasting studies project a continued rise in CKD mortality and case numbers over the coming decades, driven by demographic transitions, comorbid conditions, and healthcare system constraints [6]. National projections likewise indicate increasing CKD incidence across etiologies, underscoring the urgency of developing effective preventive and therapeutic strategies [7]. Renal anemia contributes substantially to morbidity in CKD, worsening fatigue, exercise tolerance, and quality of life, and is independently associated with higher rates of hospitalization, cardiovascular events, and mortality [2]. These impacts underscore the critical need for effective anemia therapies in CKD.

Current standard therapy for renal anemia is recombinant erythropoiesis-stimulating agents (ESAs)—such as epoetins and darbepoetin—plus iron supplementation [8]. ESAs are typically administered parenterally and require frequent dosing to maintain target hemoglobin. However, use of high-dose ESAs and higher hemoglobin targets has been linked to safety concerns: randomized trials have associated ESA therapy with an increased risk of stroke, myocardial infarction, and other adverse cardiovascular outcomes [9]. These safety warnings (including US Food and Drug Administration (FDA) black-box notices) and the burden of injections have motivated the search for alternative treatments. In recent years, prolyl hydroxylase inhibitors (PHIs)—sometimes called hypoxia-inducible factor prolyl hydroxylase (HIF-PH) inhibitors—have emerged as a novel class of oral anemia therapies. PHIs block HIF prolyl-hydroxylase enzymes, thereby stabilizing HIF transcription factors and increasing endogenous EPO production and iron mobilization [10]. Notable PHIs include roxadustat, daprodustat, vadadustat, molidustat, desidustat, and enarodustat, all of which effectively raise hemoglobin in both non-dialysis and dialysis CKD patients in clinical trials [11]. These agents have received approval in various regions for CKD anemia (e.g. roxadustat in Asia and Europe, daprodustat in Japan/USA), reflecting their promise to simplify treatment (oral dosing) and address ESA shortcomings.

Phase 3 randomized trials have demonstrated that PHIs reliably correct anemia in CKD. For example, in the ASCEND trials daprodustat (vs. epoetin/darbepoetin) achieved noninferior hemoglobin responses and similar cardiovascular event rates in dialysis and non-dialysis populations [12]. Similarly, roxadustat showed comparable efficacy to ESAs in large trials [13]. To date, multiple meta-analyses and network meta-analyses (NMAs) of PHIs versus ESAs/placebo have been published. These generally find that all PHIs raise hemoglobin significantly over placebo and perform comparably to ESAs, with no single PHI clearly superior in efficacy [14]. For instance, a recent NMA including three PHIs (roxadustat, daprodustat, vadadustat vs. placebo/ESA) found that each PHI effectively elevated hemoglobin in CKD, and that hemoglobin responses and safety outcomes were broadly similar [15]. However, most previous NMAs have either pooled dialysis and non-dialysis populations or only partially stratified by dialysis status, and none have systematically compared hemodialysis with peritoneal dialysis patients, leaving uncertainty about potential modality-specific differences in PHI efficacy and safety.

In light of these gaps, this study differs from prior work in that it directly compares all six currently approved PHIs within the same class, while using placebo and ESAs as common reference comparators to ensure network connectivity. This design allows for a unified evaluation of efficacy and safety, fully stratified by dialysis status and further distinguishing hemodialysis from peritoneal dialysis to explore the modality-specific effects. To achieve this, we conducted a network meta-analysis (NMA) capable of integrating both direct and indirect evidence, thereby enabling simultaneous comparisons and ranking of multiple treatments [16]. In this study, our primary objective is to compare the efficacy and safety of all six approved PHIs in patients with CKD-related anemia. Our secondary objective is to evaluate these outcomes stratified by dialysis status (D-CKD versus ND-CKD), since treatment effects may differ between these subpopulations. By synthesizing the totality of randomized controlled trial (RCT) data in a unified model, this analysis aims to inform clinicians and guideline panels about the optimal choice of PHI for renal anemia, balancing benefits and risks in diverse CKD populations.

## 2. Methods

This systematic review and NMA was conducted in accordance with the Preferred Reporting Items for Systematic Reviews and Meta-Analyses (PRISMA) 2020 guidelines and the PRISMA extension statement for NMAs [17,18]. The protocol was prospectively registered in the PROSPERO database (CRD420251066181) to ensure methodological transparency and reduce the risk of reporting bias. Ethical approval and informed consent were not required, as this study involved secondary analysis of published data only.

### 2.1. Data sources and search strategy

A comprehensive literature search was conducted in PubMed, MEDLINE, Embase, Cochrane Central Register of Controlled Trials, Scopus, and Web of Science from database inception to 1 May 2025, with no time restrictions. Articles in all languages were screened. As prespecified in the study protocol, an updated search was performed in May 2025 across all databases prior to final analysis, using the same predefined strategy to ensure completeness. Potentially eligible non-English studies were translated using a two-step process: initial translation with professional software followed by verification by bilingual researchers to ensure the accuracy of data extraction. The updated search identified no additional eligible RCTs in non-English languages. The search combined Medical Subject Headings (MeSH) and relevant keywords related to ‘prolyl hydroxylase inhibitors’, ‘kidney disease’, ‘hemoglobin’, and ‘randomized controlled trial’ using Boolean operators (‘AND’, ‘OR’). The full search strategy is presented in Supplementary File 1. References of all included studies and recent systematic reviews published in the past 5 years were also screened to ensure comprehensiveness. Two reviewers independently screened titles/abstracts and full texts. Discrepancies were resolved through discussion or adjudication by a third reviewer. For PubMed, the full search string was: (‘anemia’[MeSH Terms] OR ‘anemia’ [All Fields]) AND (‘chronic kidney disease’ [MeSH Terms] OR ‘chronic kidney disease’ [All Fields] OR ‘CKD’ [All Fields]) AND (‘prolyl hydroxylase inhibitor’ [All Fields] OR ‘PHI’ [All Fields] OR individual PHI drug names) AND (‘randomized controlled trial’ [Publication Type] OR randomized [Title/Abstract] OR placebo [Title/Abstract] OR randomly [Title/Abstract]).

### 2.2. Study selection

Studies were included if they met the following criteria: (1) adult participants (aged ≥18 years) diagnosed with CKD, including D-CKD and ND-CKD populations; (2) intervention group receiving one of six PHIs (roxadustat, daprodustat, molidustat, desidustat, enarodustat, or vadadustat); (3) control group receiving either standard care or an alternative comparator; (4) reporting of at least one of the following outcomes: hemoglobin levels, iron metabolism parameters (ferritin, hepcidin, transferrin saturation [TSAT], serum iron), or cardiovascular adverse events (hypertension, thrombosis); (5) RCT; and (6) no language restriction; studies published in any language were eligible.

Exclusion criteria were: (1) studies involving pediatric patients (<18 years); (2) insufficient detail regarding the intervention; (3) unavailable mean or standard deviation (SD), and no response from authors after four data requests within 6 weeks; (4) studies reporting outcomes solely in graphical format without extractable numerical values, unless precise data could be obtained directly from the study authors; (5) non-RCT designs; and (6) conference abstracts or protocols. Study eligibility was independently assessed by two reviewers according to title, abstract, and full-text screening.

### 2.3. Data extraction

Eligible studies were managed using EndNote X9 to remove duplicates. Two reviewers (YN and TW) independently extracted relevant information: study characteristics (e.g. authors, year), participant characteristics (e.g. age, sex), intervention and comparator details (drug, dosage, frequency, duration), and outcomes (see Supplementary File 2.1). Abbreviations were standardized across the manuscript, and all abbreviations (e.g. TSAT, SUCRA [surface under the cumulative ranking curve], TIW [three times a week]) were defined at first appearance. When mean and SD were missing, values were estimated using Cochrane-recommended methods, including derivation from standard errors, confidence intervals, *p*-values, or reported ranges [19]. In total, imputation was applied in seven studies, and the specific method used for each case is detailed in Supplementary File 2.2. If an included study reported multiple arms using the same intervention, relevant data were pooled using a weighted approach, in which means were averaged according to sample size and pooled SDs were calculated using standard formulas recommended by the Cochrane Handbook. For clarity, throughout the manuscript, N refers to the number of participants and k to the number of randomized trials, with trial counts reported as ‘k’ to avoid ambiguity.

### 2.4. Risk of bias assessment

The risk of bias was assessed at the study level using the Cochrane Risk of Bias 2.0 (RoB 2) tool. Domains included: randomization process, deviations from intended interventions, missing outcome data, outcome measurement, and selective reporting [20]. Disagreements were resolved through consultation with a third reviewer.

### 2.5. Treatment coding

Treatment nodes were categorized based on the type of intervention used in each study. For the purpose of analysis, studies involving dialysis-dependent patients and non-dialysis-dependent patients were evaluated separately. Within each subgroup, treatments were coded as follows: rox (roxadustat), dap (daprodustat), mol (molidustat), des (desidustat), ena (enarodustat), vad (vadadustat), esa (ESA, analyzed as an independent active comparator rather than being merged into ‘standard care’), and pbo (placebo/no active therapy). Accordingly, ‘standard care’ was not treated as a single heterogeneous category; ESA was modeled separately to avoid misclassification and minimize heterogeneity in the control group. All eligible treatments within each subgroup were considered for head-to-head comparisons using NMA. To ensure valid indirect comparisons, placebo and ESAs were employed solely as common reference comparators within the network, thereby preserving overall connectivity. No specific treatment served as a universal comparator across all studies.

### 2.6. Outcome measures

The primary efficacy outcome was the change in hemoglobin level. Secondary outcomes included serum iron and TSAT, where higher values reflect better outcomes, and ferritin and hepcidin, where lower values indicate improved status. Safety outcomes included the incidence of adverse events.

### 2.7. Data analysis

NMAs were performed using Stata version 17.0 (StataCorp, TX, USA). A random-effects model was chosen instead of a fixed-effects model because clinical and methodological heterogeneity was expected across trials, including differences in dialysis status, intervention regimens, follow-up durations, and study designs. This approach accounts for both within-study and between-study variability and provides more conservative and generalizable estimates. Between-study variance was estimated using the Restricted Maximum Likelihood (REML) method, which is widely recommended for its robustness in random-effects meta-analyses. For continuous outcomes (e.g. hemoglobin, serum iron), mean differences (MDs) with 95% confidence intervals (CIs) were calculated. For dichotomous outcomes (e.g. adverse events), odds ratios (ORs) with 95% CIs were used.

The network structure was visualized using network plots to assess treatment connectivity. Statistical heterogeneity was evaluated using the I^2^ statistic, with thresholds of 25%, 50%, and 75% indicating low, moderate, and high heterogeneity, respectively. Consistency within the network was assessed using the design-by-treatment interaction model. When the global inconsistency test indicated potential disagreement (*p* < 0.05), we conducted additional local inconsistency assessment using the node-splitting method. Detailed results of these assessments are presented in Supplementary File 7. For contrasts with ≥2 direct randomized trials, random-effects pairwise meta-analyses were also conducted and compared with the corresponding network estimates. Ranking probabilities for all interventions were estimated based on the SUCRA, where a higher SUCRA value indicates better performance.

Publication bias was evaluated using comparison-adjusted funnel plots and Egger’s test (*p* < 0.05 considered indicative of bias) [21]. Predictive interval plots were used to explore the between-study variability of the estimated effects. In response to potential small-study effects and publication bias, additional sensitivity analyses were conducted. These included: (1) re-estimating all networks after excluding trials at overall high risk of bias or high risk in any RoB 2 domain; (2) repeating analyses for outcomes with funnel-plot asymmetry or significant Egger’s tests after removing influential small studies identified by contour-enhanced funnel plots and leave-one-out diagnostics; and (3) applying trim-and-fill to pairwise contrasts with ≥10 studies. The use of trim-and-fill in NMA remains controversial; therefore, these results were interpreted with caution and regarded as exploratory sensitivity analyses rather than confirmatory evidence. Pooled effects, between-study variance, and SUCRA rankings from these analyses were compared with the primary results.

All statistical tests were two-sided, and *p*-values less than 0.05 were considered statistically significant. Bayesian NMA was further conducted within a Markov chain Monte Carlo (MCMC) framework using Stata packages ‘network’ and ‘mvmeta’. We employed vague, non-informative priors for treatment effect parameters (normal distribution with mean = 0, variance = 10,000) and for between-study variance (uniform distribution from 0 to 5) to minimize prior influence. Both fixed-effect and random-effects models were compared, with model selection guided by the Deviance Information Criterion (DIC), favoring the model with the lower DIC. Convergence of the MCMC chains was assessed using the Gelman–Rubin statistic (values < 1.05 deemed acceptable) and visual inspection of trace and density plots to confirm stability and adequate mixing. Model estimation included 50,000 burn-in iterations followed by 100,000 sampling iterations with a thinning interval of 10 to reduce autocorrelation. Posterior estimates were summarized as mean differences or odds ratios with 95% credible intervals (CrIs), and SUCRA values were calculated to quantify treatment ranking probabilities. The Bayesian framework was applied to complement frequentist analyses, provide probabilistic treatment rankings, and allow meta-regression for outcomes with high heterogeneity. To ensure transparency, the comparison of DIC values between fixed-effect and random-effects Bayesian models across all major outcomes is reported in Supplementary File 8. Subgroup analyses were also conducted for dialysis versus non-dialysis patients to assess differences in treatment effects, with interpretation considering potential limitations in power and between-study variability.

## 3. Results

### 3.1. Characteristics of included studies

**Figure 1.**
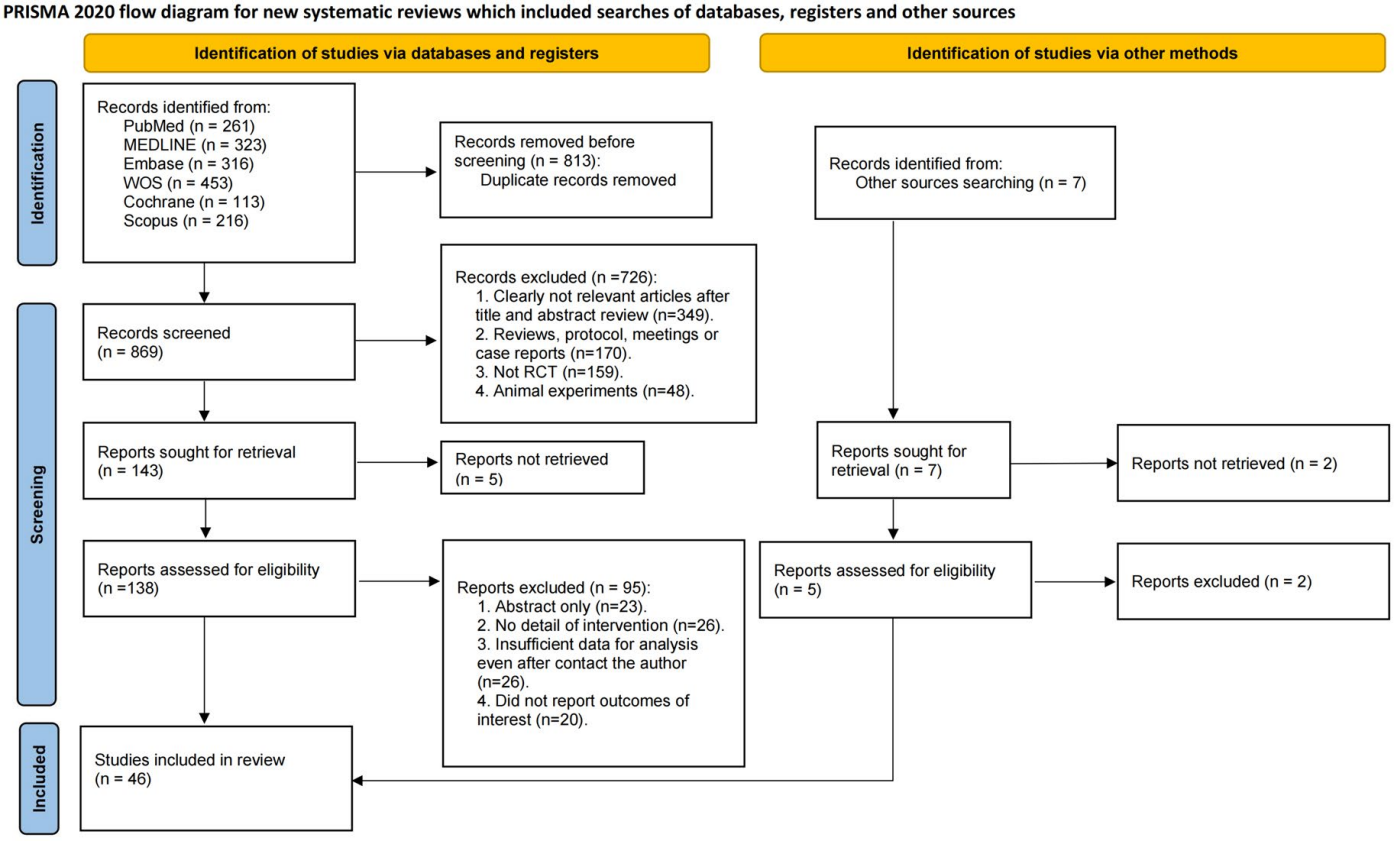
PRISMA Flow diagram of the search process for studies.

A total of 1,682 records were identified through the initial electronic search. After excluding 813 duplicates, 869 articles were screened by title and abstract. Of these, 726 were excluded, and 143 full-text articles were assessed for eligibility. Ultimately, 46 RCTs were included [22–67], involving 32,305 patients with anemia of CKD for systematic review and NMA (Figure 1).

The included studies were published between 2015 and 2025, with a median publication year of 2019. Sample sizes ranged from 12 to 1,937 participants, with a median of 30. The mean age of participants ranged from 47.6 to 72.4 years, with a median of 62.4 years. Forty-four studies reported the sex distribution, with the proportion of male participants ranging from 16% to 86% (median: 56.5%). Baseline BMI was reported in 19 studies, ranging from 20.3 to 34.2 kg/m^2^ (median: 26.8 kg/m^2^). A total of 19,379 patients were ND-CKD, and 12,926 were D-CKD.

Among the dialysis population, 15 studies exclusively enrolled patients receiving hemodialysis, 2 studies enrolled patients receiving peritoneal dialysis, and 10 studies included mixed peritoneal-hemodialysis cohorts. Due to the limited number of peritoneal dialysis studies, a separate subgroup analysis was not feasible; therefore, the primary analysis compared dialysis versus non-dialysis populations. The treatment duration ranged from 2 to 208 weeks, with a median of 52 weeks.

Among ND-CKD patients, the intervention nodes and number of trials were: daprodustat (dap; *k* = 4), desidustat (des; *k* = 2), enarodustat (ena; *k* = 2), ESAs (ESA; *k* = 14), molidustat (mol; *k* = 4), placebo (pbo; *k* = 14), roxadustat (rox; *k* = 9), and vadadustat (vad; *k* = 5). Among D-CKD patients, the nodes and number of trials were: daprodustat (dapD; *k* = 5), desidustat (desD; *k* = 1), enarodustat (enaD; *k* = 2), ESA (esaD; *k* = 24), molidustat (molD; *k* = 3), placebo (pboD; *k* = 2), roxadustat (roxD; *k* = 13), and vadadustat (vadD; *k* = 3). Detailed characteristics of the included studies are provided in Supplementary File 2.1.

### 3.2. Results of NMA

#### 3.2.1. Hemoglobin

For ND-CKD patients, 22 trials involving 16,197 participants were included. Figure 2.1 presents the network structure and direct comparisons. According to SUCRA rankings (Figure 3.1), the top three interventions for improving hemoglobin levels were roxadustat (88.6%), enarodustat (81.2%), and daprodustat (60.1%), while placebo ranked the lowest (9.0%). As shown in Table 1.1, roxadustat (MD = 1.21, 95% CI: 0.72–1.71) and enarodustat (MD = 1.11, 95% CI: 0.19–2.03) significantly increased hemoglobin compared with placebo. Additionally, roxadustat significantly outperformed ESA (MD = 0.89, 95% CI: 0.20–1.57).

**Figure 2.**
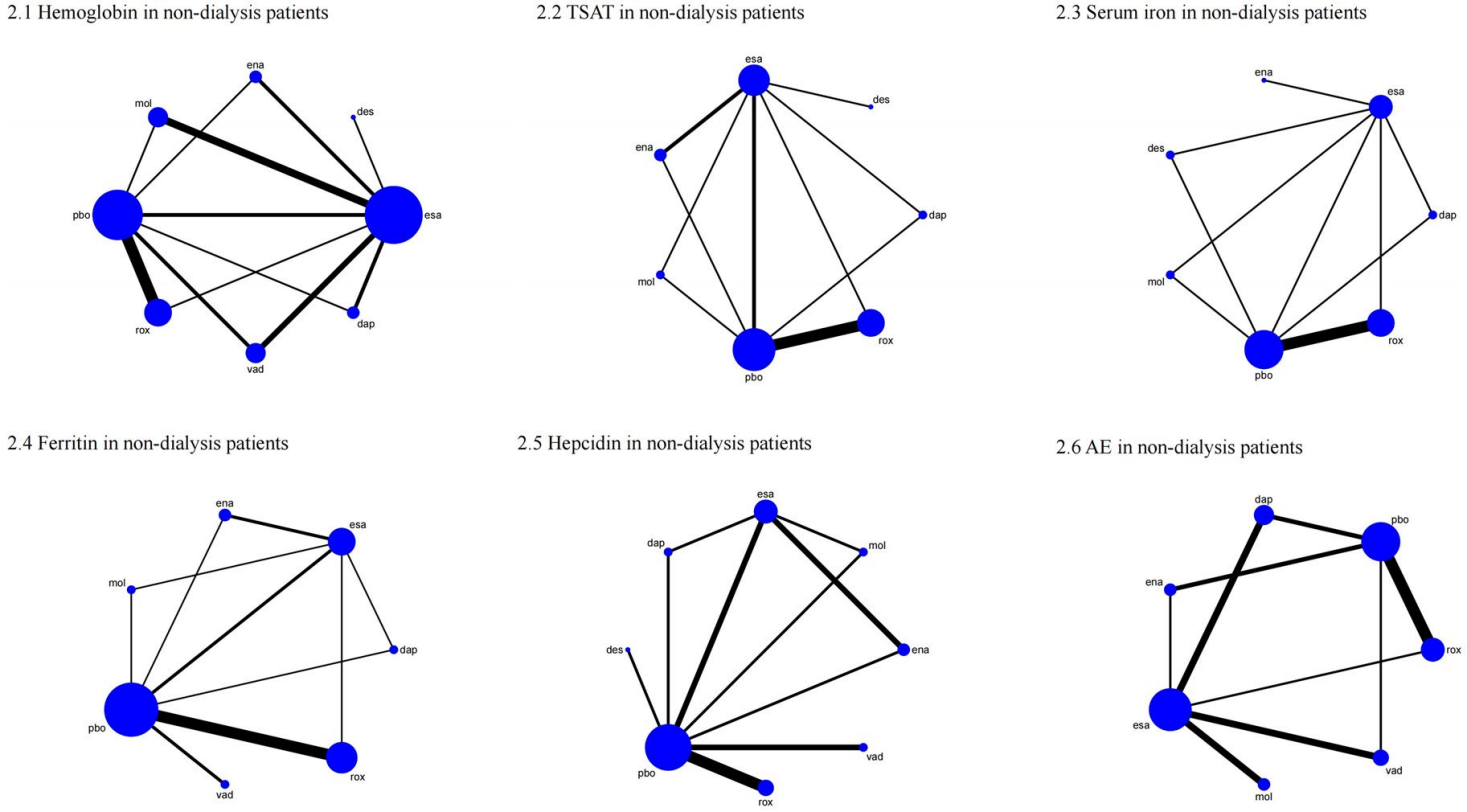
Network plot comparison of outcomes in non-dialysis patients. 1: Hemoglobin, 2: TSAT, 3: Serum iron, 4: Ferritin, 5: Hepcidin, 6: AE. TSAT: transferrin saturation; AE: adverse event; rox: roxadustat; dap: daprodustat; vad: vadadustat; des: desidustat; ena: enarodustat; mol: molidustat; pbo: placebo; esa: erythropoiesis-stimulating agent.

**Figure 3.**
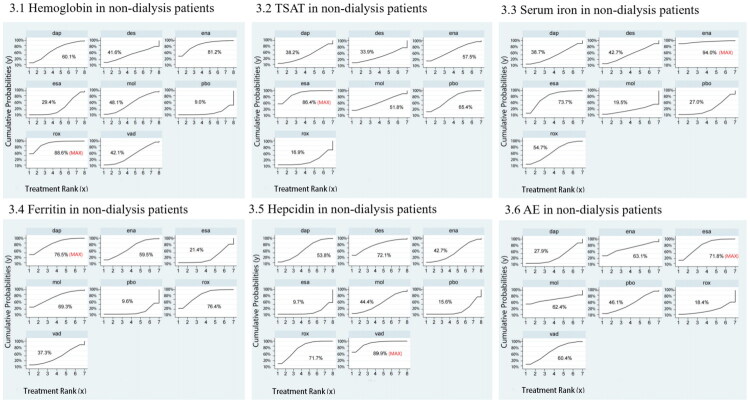
SUCRA probability ranking plot of outcomes in non-dialysis patients. 1: Hemoglobin, 2: TSAT, 3: Serum iron, 4: Ferritin, 5: Hepcidin, 6: AE. TSAT: transferrin saturation; AE: adverse event; rox: roxadustat; dap: daprodustat; vad: vadadustat; des: desidustat; ena: enarodustat; mol: molidustat; pbo: placebo; esa: erythropoiesis-stimulating agent.

**Table 1.** League table of efficacy in non-dialysis patients. **Table 1.1.** Hemoglobin in non-dialysis patients.

rox							
0.10 (−0.91,1.11)	ena						
0.49 (−0.43,1.40)	0.38 (−0.71,1.48)	dap					
0.67 (−0.22,1.55)	0.56 (−0.47,1.59)	0.18 (−0.77,1.13)	mol				
0.74 (−0.04,1.53)	0.64 (−0.36,1.64)	0.26 (−0.65,1.17)	0.08 (−0.76,0.92)	vad			
0.77 (−0.65,2.18)	0.66 (−0.83,2.16)	0.28 (−1.16,1.72)	0.10 (−1.28,1.48)	0.02 (−1.36,1.40)	des		
**0.89 (0.20,1.57)**	0.78 (−0.06,1.62)	0.40 (−0.34,1.14)	0.22 (−0.40,0.84)	0.14 (−0.46,0.74)	0.12 (−1.12,1.36)	esa	
**1.21 (0.72,1.71)**	**1.11 (0.19,2.03)**	0.73 (−0.09,1.54)	0.55 (−0.24,1.34)	0.47 (−0.19,1.13)	0.45 (−0.92,1.81)	0.33 (−0.24,0.90)	pbo

rox: roxadustat; dap: daprodustat; vad: vadadustat; des: desidustat; ena: enarodustat; mol: molidustat; pbo: placebo; esa: erythropoiesis-stimulating agent.

For D-CKD patients, 22 trials involving 10,244 participants were included. The network structure is shown in Figure 4.1. According to SUCRA rankings (Figure 5.1), the top three interventions were roxadustat (86.2%), desidustat (81.8%), and daprodustat (74.5%), while placebo was ranked the lowest (0.0%). As presented in Table 2.1, all active treatments significantly improved hemoglobin compared with placebo, including roxadustat (MD = 1.78, 95% CI: 1.32–2.24), desidustat (MD = 1.77, 95% CI: 1.26–2.27), daprodustat (MD = 1.72, 95% CI: 1.30–2.14), erythropoiesis-stimulating agent (MD = 1.62, 95% CI: 1.17–2.07), enarodustat (MD = 1.54, 95% CI: 1.10–1.98), molidustat (MD = 1.46, 95% CI: 0.95–1.97), and vadadustat (MD = 1.35, 95% CI: 0.88–1.81). Furthermore, roxadustat (MD = 0.43, 95% CI: 0.27–0.59), desidustat (MD = 0.42, 95% CI: 0.17–0.67), daprodustat (MD = 0.37, 95% CI: 0.17–0.58), and erythropoiesis-stimulating agent (MD = 0.27, 95% CI: 0.14–0.40) were significantly more effective than vadD. Roxadustat also significantly outperformed molidustat (MD = 0.32, 95% CI: 0.07–0.57) and erythropoiesis-stimulating agent (MD = 0.16, 95% CI: 0.06–0.26). To further explore the potential sources of variability in outcomes with substantial heterogeneity, a Bayesian network meta-regression was conducted. The analysis did not identify any statistically significant modifying effects, and the results are provided in Supplementary File 6.

**Figure 4.**
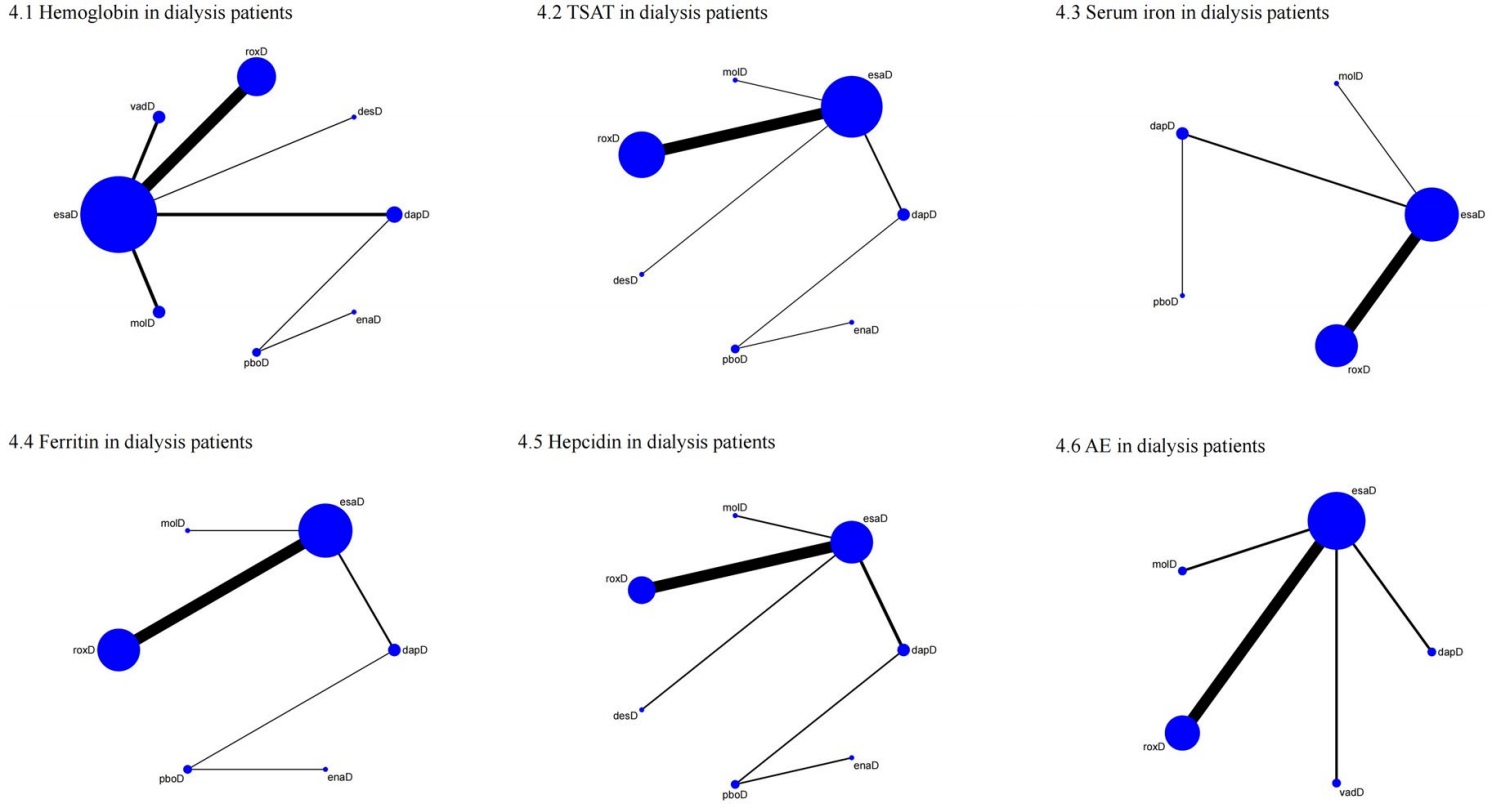
Network plot comparison of outcomes in dialysis patients. 1: Hemoglobin, 2: TSAT, 3: Serum iron, 4: Ferritin, 5: Hepcidin, 6: AE. TSAT: transferrin saturation; AE: adverse event; roxD: roxadustat; dapD: daprodustat; vadD: vadadustat; desD: desidustat; enaD: enarodustat; molD: molidustat; pboD: placebo; esaD: erythropoiesis-stimulating agent.

**Figure 5.**
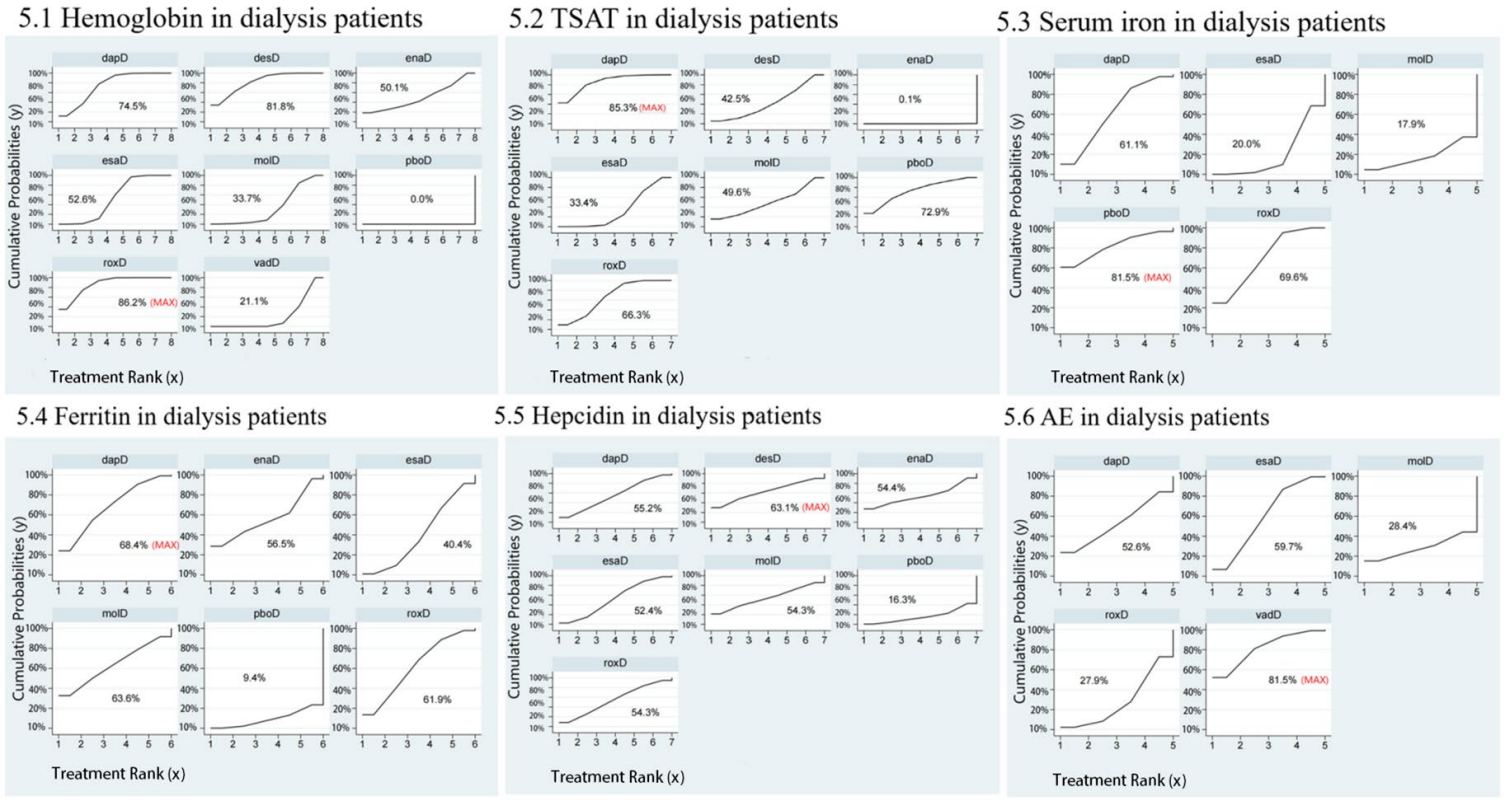
SUCRA probability ranking plot of outcomes in dialysis patients. 1: Hemoglobin, 2: TSAT, 3: Serum iron, 4: Ferritin, 5: Hepcidin, 6: AE. TSAT: transferrin saturation; AE: adverse event; roxD: roxadustat; dapD: daprodustat; vadD: vadadustat; desD: desidustat; enaD: enarodustat; molD: molidustat; pboD: placebo; esaD: erythropoiesis-stimulating agent.

**Table 2.** League table of efficacy in dialysis patients. **Table 2.1.** Hemoglobin in dialysis patients.

roxD							
0.01 (−0.23,0.25)	desD						
0.06 (−0.13,0.25)	0.05 (−0.22,0.32)	dapD					
**0.16 (0.06,0.26)**	0.15 (−0.07,0.37)	0.10 (−0.05,0.26)	esaD				
0.24 (−0.40,0.88)	0.23 (−0.44,0.89)	0.18 (−0.43,0.79)	0.08 (−0.56,0.71)	enaD			
**0.32 (0.07,0.57)**	0.31 (−0.01,0.63)	0.26 (−0.02,0.54)	0.16 (−0.07,0.39)	0.08 (−0.59,0.75)	molD		
**0.43 (0.27,0.59)**	**0.42 (0.17,0.67)**	**0.37 (0.17,0.58)**	**0.27 (0.14,0.40)**	0.19 (−0.45,0.84)	0.11 (−0.15,0.38)	vadD	
**1.78 (1.32,2.24)**	**1.77 (1.26,2.27)**	**1.72 (1.30,2.14)**	**1.62 (1.17,2.07)**	**1.54 (1.10,1.98)**	**1.46 (0.95,1.97)**	**1.35 (0.88,1.81)**	pboD

roxD: roxadustat; dapD: daprodustat; vadD: vadadustat; desD: desidustat; enaD: enarodustat; molD: molidustat; pboD: placebo; esaD: erythropoiesis-stimulating agent.

#### 3.2.2. TSAT

In ND-CKD patients, 13 studies (*N* = 4,077) were included (Figure 2.2). The top three interventions ranked by SUCRA (Figure 3.2) were erythropoiesis-stimulating agent (86.4%), placebo (65.4%), and enarodustat (57.5%), with roxadustat ranking lowest (16.9%). As shown in Table 1.2, both erythropoiesis-stimulating agent (MD = 5.24, 95% CI: 1.16–9.32) and placebo (MD = 3.63, 95% CI: 0.96–6.30) significantly increased TSAT compared with roxadustat.

**Table 1.2.** TSAT in non-dialysis patients.

esa						
1.61 (−2.16,5.37)	pbo					
1.99 (−2.83,6.81)	0.38 (−4.95,5.71)	ena				
2.50 (−4.01,9.01)	0.89 (−5.70,7.48)	0.51 (−7.32,8.34)	mol			
3.60 (−1.35,8.56)	1.99 (−2.79,6.78)	1.61 (−4.95,8.17)	1.10 (−6.62,8.83)	dap		
4.20 (−2.05,10.45)	2.59 (−4.71,9.89)	2.21 (−5.68,10.10)	1.70 (−7.32,10.73)	0.60 (−7.38,8.57)	des	
**5.24 (1.16,9.32)**	**3.63 (0.96,6.30)**	3.25 (−2.47,8.98)	2.74 (−4.19,9.68)	1.64 (−3.63,6.91)	1.04 (−6.42,8.51)	rox

TSAT: transferrin saturation; rox: roxadustat; dap: daprodustat; des: desidustat; ena: enarodustat; mol: molidustat; pbo: placebo; esa: erythropoiesis-stimulating agent.

In D-CKD patients, 18 studies (*N* = 6,664) were analyzed (Figure 4.2). SUCRA rankings (Figure 5.2) showed daprodustat (85.3%), placebo (72.9%), and roxadustat (66.3%) as the top interventions, while enarodustat ranked the lowest (0.1%). As shown in Table 2.2, all treatments (daprodustat, placebo, roxadustat, molidustat, desidustat, erythropoiesis-stimulating agent) significantly outperformed enarodustat. Daprodustat (MD = 3.07, 95% CI: 0.22–5.91) and roxadustat (MD = 1.66, 95% CI: 0.43–2.89) also outperformed erythropoiesis-stimulating agent.

**Table 2.2.** TSAT in dialysis patients.

dapD						
0.63 (−2.42,3.68)	pboD					
1.41 (−1.68,4.50)	0.78 (−3.56,5.12)	roxD				
2.47 (−3.30,8.23)	1.84 (−4.69,8.36)	1.06 (−4.11,6.22)	molD			
2.83 (−1.58,7.23)	2.20 (−3.16,7.56)	1.42 (−2.16,5.00)	0.36 (−5.68,6.40)	desD		
**3.07 (0.22,5.91)**	2.44 (−1.74,6.61)	**1.66 (0.43,2.89)**	0.60 (−4.42,5.62)	0.24 (−3.12,3.60)	esaD	
**12.96 (7.91,18.01)**	**12.33 (8.30,16.36)**	**11.55 (5.63,17.47)**	**10.49 (2.83,18.16)**	**10.13 (3.43,16.84)**	**9.89 (4.09,15.69)**	enaD

TSAT: transferrin saturation; roxD: roxadustat; dapD: daprodustat; desD: desidustat; enaD: enarodustat; molD: molidustat; pboD: placebo; esaD: erythropoiesis-stimulating agent.

#### 3.2.3. Serum iron

Among ND-CKD patients, 13 studies (*N* = 6,650) were included (Figure 2.3). SUCRA rankings (Figure 3.3) identified enarodustat (94.0%), erythropoiesis-stimulating agent (73.7%), and roxadustat (54.7%) as the top treatments; molidustat ranked lowest (19.5%). No significant differences were observed among interventions (Table 1.3).

**Table 1.3.** Serum iron in non-dialysis patients.

ena						
10.65 (−5.72,27.02)	esa					
13.31 (−4.56,31.19)	2.66 (−4.53,9.86)	rox				
14.60 (−3.81,33.01)	3.95 (−4.49,12.39)	1.29 (−8.67,11.24)	des			
15.05 (−3.22,33.33)	4.40 (−3.74,12.54)	1.74 (−6.61,10.09)	0.45 (−10.60,11.50)	dap		
16.22 (−1.62,34.07)	5.57 (−1.54,12.69)	2.91 (−1.21,7.03)	1.62 (−8.12,11.37)	1.17 (−6.57,8.91)	pbo	
19.56 (−1.38,40.51)	8.91 (−4.16,21.99)	6.25 (−7.20,19.70)	4.96 (−10.17,20.09)	4.51 (−9.95,18.97)	3.34 (−9.77,16.45)	mol

rox: roxadustat; dap: daprodustat; des: desidustat; ena: enarodustat; mol: molidustat; pbo: placebo; esa: erythropoiesis-stimulating agent.

In D-CKD patients, 18 studies (*N* = 6,018) were included (Figure 4.3). The top interventions based on SUCRA (Figure 5.3) were placebo (81.5%), roxadustat (69.6%), and daprodustat (61.1%), while molidustat ranked lowest (17.9%). Roxadustat significantly increased serum iron compared with erythropoiesis-stimulating agent (MD = 6.19, 95% CI: 2.81–9.58) (Table 2.3).

**Table 2.3.** Serum iron in dialysis patients.

pboD				
3.04 (−10.33,16.40)	roxD			
3.83 (−6.72,14.38)	0.79 (−7.40,8.99)	dapD		
9.23 (−3.70,22.16)	**6.19 (2.81,9.58)**	5.40 (−2.07,12.87)	esaD	
11.83 (−6.98,30.64)	8.79 (−5.28,22.87)	8.00 (−7.57,23.57)	2.60 (−11.06,16.26)	molD

roxD: roxadustat; dapD: daprodustat; molD: molidustat; pboD: placebo; esaD: erythropoiesis-stimulating agent.

#### 3.2.4. Ferritin

For ND-CKD patients, 15 studies (*N* = 6,551) were included (Figure 2.4). According to SUCRA rankings (Figure 3.4), the most effective interventions for reducing ferritin levels were daprodustat (76.5%), roxadustat (76.4%), and molidustat (69.3%), while placebo ranked lowest (9.6%). As presented in Table 1.4, both daprodustat (MD = −53.03, 95% CI: −97.93 to −8.13) and roxadustat (MD = −51.31, 95% CI: −74.92 to −27.70) significantly reduced ferritin compared with placebo. Additionally, roxadustat was significantly more effective than ESA (MD = −41.38, 95% CI: −80.98 to −1.79). A reduction in ferritin may reflect enhanced iron mobilization and utilization; however, this should be interpreted with caution, as it can also indicate functional iron deficiency requiring supplementation.

**Table 1.4.** Ferritin in non-dialysis patients.

dap						
−1.73 (−51.49,48.04)	rox					
−4.82 (−72.79,63.15)	−3.10 (−63.95,57.76)	mol				
−14.61 (−72.73,43.52)	−12.88 (−63.89,38.12)	−9.79 (−77.24,57.67)	ena			
−32.48 (−94.08,29.12)	−30.76 (−79.13,17.62)	−27.66 (−98.56,43.24)	−17.87 (−80.83,45.08)	vad		
−43.11 (−88.04,1.82)	**−41.38 (−80.98,−1.79)**	−38.29 (−94.49,17.92)	−28.50 (−70.28,13.28)	−10.63 (−65.21,43.95)	esa	
**−53.03 (−97.93,−8.13)**	**−51.31 (−74.92,−27.70)**	−48.21 (−105.22,8.80)	−38.42 (−85.12,8.27)	−20.55 (−62.76,21.66)	−9.92 (−44.63,24.78)	pbo

rox: roxadustat; dap: daprodustat; vad: vadadustat; ena: enarodustat; mol: molidustat; pbo: placebo; esa: erythropoiesis-stimulating agent.

For D-CKD patients, 16 studies involving 6,100 participants were included (Figure 4.4). The top-ranked treatments by SUCRA (Figure 5.4) were daprodustat (68.4%), molidustat (63.6%), and roxadustat (61.9%), with placebo ranked lowest (9.4%). However, as shown in Table 2.4, there were no statistically significant differences among treatment arms.

**Table 2.4.** Ferritin in dialysis patients.

dapD					
−1.92 (−106.96,103.11)	molD				
−8.88 (−83.44,65.67)	−6.96 (−91.30,77.39)	roxD			
−17.21 (−140.72,106.30)	−15.29 (−177.43,146.84)	−8.33 (−152.60,135.94)	enaD		
−23.42 (−92.30,45.45)	−21.50 (−100.81,57.81)	−14.54 (−43.26,14.18)	−6.21 (−147.63,135.21)	esaD	
−84.71 (−180.06,10.64)	−82.78 (−224.64,59.08)	−75.82 (−196.86,45.21)	−67.49 (−146.00,11.02)	−61.28 (−178.91,56.34)	pboD

roxD: roxadustat; dapD: daprodustat; enaD: enarodustat; molD: molidustat; pboD: placebo; esaD: erythropoiesis-stimulating agent.

#### 3.2.5. Hepcidin

Among ND-CKD patients, 12 studies (*N* = 3,053) were included (Figure 2.5). Based on SUCRA rankings (Figure 3.5), the top three treatments for reducing hepcidin were vadadustat (89.9%), desidustat (72.1%), and roxadustat (71.7%), while erythropoiesis-stimulating agent was ranked lowest (9.7%). As shown in Table 1.5, both vadadustat (MD = −63.03, 95% CI: −104.75 to −21.32) and roxadustat (MD = −44.51, 95% CI: −79.40 to −9.62) significantly decreased hepcidin compared with ESA. Vadadustat (MD = −57.96, 95% CI: −89.15 to −26.77) and roxadustat (MD = −39.43, 95% CI: −60.70 to −18.17) were also significantly better than placebo. Decreased hepcidin may indicate improved iron mobilization *via* reduced inflammation-mediated sequestration, but clinicians should also be vigilant for possible functional iron deficiency.

**Table 1.5.** Hepcidin in non-dialysis patients.

vad							
−14.14 (−76.35,48.07)	des						
−18.52 (−56.28,19.24)	−4.39 (−62.26,53.49)	rox					
−32.76 (−77.90,12.38)	−18.62 (−81.56,44.32)	−14.23 (−53.18,24.71)	dap				
−38.92 (−89.48,11.65)	−24.78 (−91.71,42.16)	−20.39 (−65.50,24.72)	−6.16 (−53.62,41.30)	mol			
−40.64 (−87.83,6.55)	−26.50 (−90.96,37.96)	−22.12 (−63.51,19.28)	−7.88 (−50.37,34.60)	−1.73 (−49.88,46.43)	ena		
**−57.96 (−89.15,−26.77)**	−43.82 (−97.65,10.01)	**−39.43 (−60.70,−18.17)**	−25.20 (−57.83,7.43)	−19.04 (−58.83,20.75)	−17.32 (−52.78,18.15)	pbo	
**−63.03 (−104.75,−21.32)**	−48.89 (−109.42,11.63)	**−44.51 (−79.40,−9.62)**	−30.27 (−62.77,2.23)	−24.12 (−63.71,15.48)	−22.39 (−54.05,9.26)	−5.07 (−32.75,22.61)	esa

rox: roxadustat; dap: daprodustat; vad: vadadustat; des: desidustat; ena: enarodustat; mol: molidustat; pbo: placebo; esa: erythropoiesis-stimulating agent.

In D-CKD patients, 13 studies (*N* = 3,675) were included (Figure 4.5). According to SUCRA (Figure 5.5), the top-ranked interventions for reducing hepcidin were desidustat (63.1%), daprodustat (55.2%), and enarodustat (54.4%), with placebo ranked lowest (16.3%). No significant differences were detected among the interventions (Table 2.5).

**Table 2.5.** Hepcidin in dialysis patients.

desD						
−14.53 (−157.76,128.70)	dapD					
−18.91 (−236.52,198.70)	−4.38 (−168.20,159.45)	enaD				
−12.70 (−177.94,152.54)	1.83 (−140.08,143.74)	6.21 (−210.53,222.95)	molD			
−15.56 (−141.79,110.66)	−1.03 (−94.66,92.59)	3.34 (−185.34,192.03)	−2.86 (−127.58,121.85)	roxD		
−17.40 (−135.05,100.25)	−2.87 (−84.57,78.83)	1.51 (−181.56,184.58)	−4.70 (−120.73,111.33)	−1.84 (−47.56,43.89)	esaD	
−86.99 (−271.02,97.04)	−72.46 (−188.01,43.10)	−68.08 (−184.21,48.05)	−74.29 (−257.30,108.72)	−71.43 (−220.15,77.30)	−69.59 (−211.11,71.93)	pboD

roxD: roxadustat; dapD: daprodustat; desD: desidustat; enaD: enarodustat; molD: molidustat; pboD: placebo; esaD: erythropoiesis-stimulating agent.

#### 3.2.6. AE

Among ND-CKD patients, 21 studies involving 14,975 participants reported AE outcomes (Figure 2.6). Based on SUCRA rankings (Figure 3.6), the safest interventions in terms of AE incidence were erythropoiesis-stimulating agent (71.8%), enarodustat (63.1%), and molidustat (62.4%), while roxadustat ranked lowest (18.4%). As presented in Table 1.6, ESA significantly reduced AE incidence compared with daprodustat (OR = 0.85, 95% CI: 0.74–0.98).

**Table 1.6.** Adverse event in non-dialysis patients.

esa						
1.01 (0.60,1.71)	ena					
1.04 (0.47,2.33)	1.03 (0.40,2.68)	mol				
0.97 (0.85,1.09)	0.95 (0.56,1.62)	0.93 (0.41,2.08)	vad			
0.90 (0.68,1.19)	0.89 (0.54,1.46)	0.86 (0.37,2.02)	0.93 (0.69,1.25)	pbo		
**0.85 (0.74,0.98)**	0.84 (0.50,1.42)	0.81 (0.36,1.84)	0.88 (0.73,1.06)	0.94 (0.72,1.23)	dap	
0.77 (0.53,1.11)	0.76 (0.43,1.35)	0.74 (0.30,1.78)	0.80 (0.54,1.17)	0.85 (0.62,1.17)	0.90 (0.63,1.31)	rox

rox: roxadustat; dap: daprodustat; vad: vadadustat; ena: enarodustat; mol: molidustat; pbo: placebo; esa: erythropoiesis-stimulating agent.

In D-CKD patients, 15 studies (*N* = 5,883) were included (Figure 4.6). The top-ranked interventions by SUCRA (Figure 5.6) were vadadustat (81.5%), erythropoiesis-stimulating agent (59.7%), and daprodustat (52.6%), while roxadustat ranked lowest (27.9%). However, no statistically significant differences in AE incidence were observed among treatment arms (Table 2.6).

**Table 2.6.** Adverse event in dialysis patients.

vadD				
0.88 (0.65,1.18)	esaD			
0.86 (0.49,1.51)	0.97 (0.60,1.58)	dapD		
0.65 (0.26,1.67)	0.74 (0.31,1.81)	0.76 (0.28,2.10)	molD	
0.74 (0.50,1.10)	0.85 (0.66,1.09)	0.87 (0.49,1.55)	1.14 (0.45,2.87)	roxD

roxD: roxadustat; dapD: daprodustat; vadD: vadadustat; molD: molidustat; esaD: erythropoiesis-stimulating agent.

For contrasts with ≥2 direct RCTs, random-effects pairwise meta-analyses yielded results consistent with the corresponding network estimates, with overlapping 95% confidence/credible intervals for all primary outcomes. Node-splitting detected no significant local inconsistency for hemoglobin, iron indices, or adverse events (all *p* > 0.05).

### 3.3. Risk of bias and publication bias

Among the 46 included RCTs, 22 were assessed as having an overall low risk of bias, 19 as having some concerns, and 5 as high risk. Regarding the randomization process, 35 studies were rated as low risk, 9 as some concerns, and 2 as high risk. In the domain of deviations from intended interventions, 38 were low risk, 5 had some concerns, and 3 were high risk. For missing outcome data, 31 studies were low risk, 12 had some concerns, and 3 were high risk. Regarding outcome measurement, 43 studies were low risk, 2 had some concerns, and 1 was high risk. All 46 studies were assessed as low risk for selective reporting (Supplementary File 3).

Funnel plots were used to assess potential publication bias (Supplementary File 4). Most scatterplots were approximately symmetric along the vertical axis, indicating no major asymmetry. However, Figures S4.1 to S4.10 and S4.12 displayed varying degrees of asymmetry, suggesting potential publication bias, whereas Figure S4.11 showed a relatively uniform distribution. Egger’s tests identified possible small-study effects in two comparisons: serum iron in ND-CKD patients (Figure S4.5) and AE in D-CKD patients (Figure S4.12), with *p*-values < 0.05. For all other outcomes, Egger’s tests yielded *p*-values > 0.05, indicating no significant evidence of publication bias in the overall analysis.

### 3.4. Sensitivity analyses

After excluding trials at overall high risk of bias or high risk within any RoB 2 domain, pooled estimates and SUCRA ranks for all primary and key secondary outcomes were materially unchanged. Roxadustat’s superiority over placebo and its advantage versus ESA for hemoglobin in ND-CKD and D-CKD persisted, and the class-level hierarchy remained stable. For outcomes with evidence of small-study effects (serum iron in ND-CKD; adverse events in D-CKD), removal of influential small studies attenuated funnel-plot asymmetry and eliminated the Egger signal. Trim-and-fill imputations produced only minimal shifts in point estimates without altering conclusions or treatment rankings.

## 4. Discussion

Based on data from 46 RCTs encompassing 32,305 patients with anemia of CKD, this comprehensive NMA systematically evaluated and compared the efficacy and safety profiles of six PHIs across both D-CKD and ND-CKD populations. Several key findings emerged. First, roxadustat demonstrated the most consistent and superior efficacy in improving hemoglobin levels among all PHIs across both ND-CKD and D-CKD populations. It ranked highest overall and outperformed placebo and ESAs, indicating its robust hematopoietic potential regardless of dialysis status. Second, PHIs exhibited dialysis-status-dependent effects on iron metabolism indices, with ESA and vadadustat demonstrating greater efficacy in enhancing TSAT and reducing hepcidin in ND-CKD patients, while daprodustat and roxadustat performed more favorably in D-CKD populations. Notably, roxadustat significantly improved serum iron levels in dialysis patients, and both vadadustat and roxadustat were superior to ESA in lowering hepcidin among non-dialysis patients, underscoring a potential mechanistic distinction between PHIs and conventional ESA therapy in iron handling. Third, roxadustat was associated with a higher likelihood of adverse events in ND-CKD patients, suggesting potential safety concerns in this subgroup. ESA remained the safest option in terms of overall adverse event risk, particularly in the non-dialysis setting. Although no statistically significant differences in AE incidence were observed among PHIs in the dialysis subgroup, roxadustat consistently ranked lowest in safety profiles across both populations. Overall, these data highlight a tradeoff between efficacy and tolerability and support tailoring PHI choice by dialysis status.

Hemoglobin is the primary therapeutic target in the management of anemia associated with CKD, as low hemoglobin levels contribute to tissue hypoxia, fatigue, impaired cognitive and physical functioning, and increased cardiovascular morbidity and mortality [68]. Accordingly, correcting hemoglobin is central to improving quality of life and outcomes in both D-CKD and ND-CKD populations. In the present NMA, roxadustat demonstrated the most consistent and pronounced efficacy in increasing hemoglobin across both ND-CKD and D-CKD subgroups, ranking highest among all evaluated agents. This finding supports prior phase III trials, such as the ROCKIES and DOLOMITES studies, which reported that roxadustat was non-inferior or even superior to epoetin alfa and placebo in raising hemoglobin levels in both dialysis and nondialysis settings [69]. Contrary to some previous meta-analyses suggesting class-wide equivalence [70], our analysis suggests a more distinct advantage for roxadustat, particularly when compared head-to-head within the PHI class.

Several pharmacological and mechanistic factors may explain the superior performance of roxadustat. First, unlike ESAs which stimulate erythropoiesis through direct EPO receptor activation, roxadustat stabilizes HIFs, enhancing endogenous EPO production in a physiologic range and simultaneously promoting iron metabolism by suppressing hepcidin [71]. This dual mechanism is especially advantageous in ND-CKD patients, who often have functional iron deficiency and chronic inflammation that limit ESA responsiveness. Roxadustat has also been shown to increase intestinal iron absorption and improve mobilization from stores, thereby addressing both erythropoietic drive and iron availability [41]. In D-CKD patients, where inflammation and ESA resistance are more common, roxadustat’s broader activation of HIF-responsive pathways may offer additional benefits. Moreover, pharmacokinetic differences (such as its oral administration, steady-state erythropoietic stimulation, and activity that are consistent despite dialysis clearance) may contribute to its superior hemoglobin profile in dialysis populations [72]. Collectively, these properties support roxadustat as a practical first-line option where ESA response is suboptimal or oral therapy is preferred.

Beyond hemoglobin correction, effective management of renal anemia also requires optimization of iron homeostasis, as iron availability is a critical determinant of erythropoietic response. Dysregulated iron metabolism, characterized by elevated hepcidin levels, impaired intestinal absorption, and iron sequestration, is particularly prevalent in CKD patients due to chronic inflammation and reduced renal clearance [73]. Thus, iron-related biomarkers such as TSAT, serum iron, ferritin, and hepcidin provide essential insight into the mechanistic efficacy of anemia therapies beyond erythropoiesis stimulation. Interpretation of ferritin and hepcidin should be contextualized by dialysis status and inflammatory tone; declines in these markers may indicate enhanced iron mobilization and attenuation of inflammatory blockade, yet warrant vigilance for functional iron deficiency, which is distinct from absolute deficiency and may necessitate iron supplementation. This dual interpretation aligns with the inflammation-iron metabolism framework and is clinically relevant when tailoring iron management strategies. Our NMA revealed the nuanced effects of PHIs on iron parameters, with notable variation between dialysis and non-dialysis populations. In ND-CKD patients, traditional ESAs and vadadustat were more favorable in preserving TSAT and reducing hepcidin, while PHIs such as roxadustat and daprodustat demonstrated relatively inferior performance on these markers. Conversely, in D-CKD patients, daprodustat and roxadustat outperformed other interventions in improving TSAT and serum iron levels, indicating their potential to mobilize iron more effectively under dialysis-related inflammatory conditions. Notably, roxadustat significantly increased serum iron in D-CKD but not in ND-CKD, and hepcidin-lowering effects observed with vadadustat and roxadustat in ND-CKD were not statistically significant in the dialysis subgroup. These dialysis-dependent patterns suggest that inflammation, iron losses, and altered clearance shape biomarker responses and may influence PHI selection.

These findings are partially consistent with previous reports suggesting that PHIs exert iron-modulatory effects by inhibiting hepcidin synthesis through HIF pathway activation [74]. However, our results also emphasize that not all PHIs exert uniform effects on iron metabolism, and their efficacy appears context-dependent. In ND-CKD, where residual kidney function and lower inflammation allow for more dynamic iron regulation, ESAs may better preserve iron balance due to their established dosing protocols. In contrast, PHIs such as roxadustat and daprodustat may offer superior benefit in D-CKD by overcoming ESA resistance and enhancing iron utilization under inflammatory blockade. These subgroup differences may be attributed to dialysis-related iron losses, reduced hepcidin clearance, and systemic inflammation, all of which attenuate iron mobilization. PHIs, by reducing hepcidin expression and promoting iron absorption and release from macrophage stores, are better suited to address these deficits in D-CKD [8,75]. That said, TSAT is a surrogate: improvements may not directly translate into survival or cardiovascular benefit, and confirmation in trials with hard endpoints (all-cause mortality, MACE) is needed. The differential modulation of iron markers across dialysis strata underscores the importance of tailoring anemia therapies not only to hemoglobin targets but also to iron status and dialysis context. In practice, aligning PHI choice with iron phenotype and dialysis modality may optimize both erythropoiesis and iron handling.

While improving hemoglobin and iron metabolism is central to the treatment of renal anemia, the safety profile of anemia therapies, particularly their impact on AEs, is equally critical in guiding clinical decision-making. CKD patients, especially those undergoing dialysis, often present with significant cardiovascular comorbidities, inflammation, and altered drug clearance, all of which may amplify the risk of treatment-related complications [13,76]. Therefore, understanding the relative safety of PHIs is essential for evaluating their net clinical benefit. Our NMA identified notable differences in the AE profiles of PHIs across dialysis strata. In ND-CKD patients, roxadustat was consistently associated with a higher incidence of AEs and ranked lowest in SUCRA-based safety rankings, whereas ESAs demonstrated a more favorable AE profile and significantly lower risk compared with daprodustat. In contrast, among D-CKD patients, no statistically significant differences in AE incidence were observed between interventions; however, roxadustat still ranked lowest, and vadadustat emerged as the top-ranked treatment in terms of safety. Although the included RCTs provided overall AE data, most did not stratify events by severity or subtype (e.g. cardiovascular vs. gastrointestinal), thereby precluding subgroup analyses for specific AEs such as thromboembolic events or hypertension. This lack of granularity limits in-depth exploration of the observed higher AE risk with roxadustat in ND-CKD, and future trials should adopt standardized AE classification and detailed reporting to enable more nuanced safety assessments.

These observations point to a possible safety signal with roxadustat in ND-CKD, despite broadly similar AE rates among PHIs in dialysis. Previous large-scale trials have reported mixed safety outcomes for PHIs. For instance, the ASCEND-ND trial identified an increased risk of cardiovascular events with roxadustat compared to darbepoetin in ND-CKD patients, aligning with our observation of higher AE risk in this subgroup [63]. Conversely, other studies, including those evaluating vadadustat and daprodustat, reported noninferior or similar AE rates to ESA comparators, particularly in dialysis-dependent populations [58]. These differences may reflect not only drug-specific safety profiles but also population-level factors influencing AE susceptibility. The increased AE risk associated with roxadustat in ND-CKD may stem from its potent and rapid erythropoietic stimulation, which could predispose patients to hypertension, thromboembolic events, and cardiovascular strain, especially in those with preserved renal function and limited uremic buffering [77]. Moreover, HIF stabilization influences a wide range of downstream targets, including angiogenic and metabolic pathways, which may have unintended systemic effects [78]. In contrast, D-CKD patients, who typically receive closer clinical monitoring and more frequent interventions (e.g. dialysis-related fluid and blood pressure control), may be better equipped to tolerate these effects, potentially explaining the more favorable safety signals observed in this subgroup [79]. Accordingly, PHI selection should weigh cardiovascular risk and monitoring capacity, with heightened vigilance when prescribing roxadustat to ND-CKD patients.

This study provides clinically actionable insights into the comparative efficacy and safety of PHIs across D-CKD and ND-CKD populations, a topic of increasing relevance as these agents continue to gain global regulatory approval. Our findings suggest that roxadustat may be a preferred agent for hemoglobin correction in both ND-CKD and D-CKD patients due to its superior efficacy. However, its safety profile, particularly in non-dialysis patients, necessitates a cautious, individualized approach to treatment. These nuanced results reinforce the importance of dialysis status in modulating therapeutic response and risk. By ranking all six PHIs alongside ESAs and placebo, our analysis offers a practical hierarchy to guide treatment selection that integrates both efficacy and tolerability. This evidence may also inform updates to international guidelines, which have yet to incorporate head-to-head comparative data on PHIs due to the absence of direct trials. In sum, a precision approach—aligning therapy with dialysis modality, iron status, and cardiovascular risk—appears warranted.

This study has several important strengths. First, it represents the most comprehensive and up-to-date NMA of PHIs to date, synthesizing data from 46 RCTs, and evaluating both efficacy and iron metabolism outcomes across dialysis strata. Second, we performed rigorous SUCRA-based treatment rankings and used a dual frequentist-Bayesian analytic framework, enhancing the robustness, transparency, and interpretability of our findings. These methodological approaches strength the generalizability and clinical utility of our conclusions. Nonetheless, several limitations should be acknowledged. First, although indirect comparisons *via* NMA offer a powerful tool in the absence of head-to-head trials, they remain susceptible to underlying heterogeneity and residual confounding due to differences in trial design, populations, and dosing strategies. Second, our subgroup analyses by dialysis status, while prespecified, may be underpowered in certain treatment arms, particularly for rare safety outcomes. These analyses were intended to distinguish dialysis-dependent from non-dialysis patients; no formal interaction tests were performed. Therefore, the findings should be interpreted as descriptive rather than confirmatory, with caution given the limited power and variability. Third, in the dialysis-dependent cohort, most included trials did not provide disaggregated outcome data for patients receiving hemodialysis versus those receiving peritoneal dialysis. Given the established differences in anemia pathophysiology, inflammatory burden, iron losses, fluid status, and potential variation in pharmacokinetics and treatment responsiveness between these modalities, the inability to conduct modality-specific analyses limits the granularity and clinical applicability of our findings. This limitation reflects gaps in the evidence base and underscores the need for future trials that stratify and report by dialysis modality. Fourth, although factors such as CRP levels and dialysis frequency are clinically relevant to the inflammation-iron metabolism hypothesis, these variables were not consistently reported across trials and therefore could not be incorporated into the meta-regression. The analyses were restricted to variables consistently available across studies, namely baseline TSAT, dialysis frequency, mean age, publication year, sample size, PHI starting dose, and male proportion. While clinically plausible, these covariates do not capture all potential modifiers, and unmeasured factors may still influence the results. Fifth, although prognostic endpoints such as MACE are of substantial clinical interest, their reporting was limited and inconsistent across included trials. Only a minority of studies—such as those analyzed by Sackeyfio et al.—provided sufficient MACE data, precluding reliable pooling or network-level comparisons [15]. While Sackeyfio et al. found no significant difference in MACE risk between PHIs and ESAs, our broader safety analysis, particularly the lower SUCRA ranking of roxadustat in ND-CKD, may differ due to variations in endpoint definitions, follow-up durations, and study inclusion criteria. Sixth, although we attempted to adjust for missing variance data using the validated methods, the imputation of summary statistics in some studies may introduce bias. Although asymmetry was detected for selected outcomes, complementary sensitivity analyses (high-risk-of-bias exclusion, influence diagnostics, and trim-and-fill) did not materially change effect estimates or rankings; nevertheless, residual small-study or reporting biases cannot be completely excluded. As the use of trim-and-fill in the context of NMA remains methodologically debated, the results derived from this approach should be regarded as exploratory and interpreted with caution rather than as definitive evidence. Future head-to-head RCTs with standardized outcomes, longer follow-up, modality-specific reporting, and harmonized safety endpoints are needed to validate and extend these findings.

## 5. Conclusion

This NMA of 46 RCTs provides the most comprehensive comparison to date of PHIs in CKD-related anemia. Roxadustat demonstrated the most consistent efficacy in raising hemoglobin across both dialysis and non-dialysis populations, while daprodustat and vadadustat showed favorable iron-modulating effects in dialysis-dependent patients, ESA was associated with better safety profiles. However, the efficacy gains of roxadustat were accompanied by a higher incidence of adverse events—particularly in non-dialysis patients—highlighting the need for careful patient selection, individualized risk assessment, and close safety monitoring. These findings highlight the importance of individualized PHI selection based on dialysis status while balancing efficacy with the potential safety risks. This study informs clinical decision-making and supports the integration of precision strategies in anemia management for CKD.

## Supplementary Material

Supplementary_Materials.docx

## Data Availability

All data generated or analyzed during this study are included in this published article and its supplementary information files. Specifically, the final extracted dataset is provided in Supplementary File 9, and the full Stata code used for the NMA is provided in Supplementary File 10 to ensure reproducibility and methodological transparency.
