# Supplementary material for: Efficacy and safety of prolyl hydroxylase inhibitors for anemia in chronic kidney disease: a network meta-analysis
Source: Ren Fail. 2026 Feb 3;48(1):2616572. doi: 10.1080/0886022X.2026.2616572 (PMC12872084; doi:10.1080/0886022X.2026.2616572)
Supplement: Supplementary_Materials.docx [file IRNF_A_2616572_SM8847.docx]

**Supplementary Materials**

**Table of Contents:**

Supplementary 1.1: PRISMA checklist…………………………………...……………………………..2

Supplementary 1.2: Search strategy……………………………………………………………………..5

Supplementary 2.1: Characteristics of studies and subjects included in the review……………………..7

Supplementary 2.2: Studies with imputed or estimated standard deviations (SDs)……………………12

Supplementary 3: Risk of Bias………………………………………………………………………….13

Supplementary 4: Publication bias……………………………………………………………………...16

Supplementary 5: Forest plot…………………………………………………………………………...22

Supplementary 6: Meta regression……………………………………………………………………...28

Supplementary 7: Node splitting method results……………………………………………………….29

Supplementary 8: Comparison of Deviance Information Criterion (DIC) between Bayesian fixed-effect and random-effects models……………………………………………………………………………..31

Supplementary 9: Dataset Used for Network Meta-Analysis…………………………………………..32

Supplementary 10: Stata Code for Network Meta-Analysis………...………………………………….44

**Supplementary 1.1: PRISMA Checklist**

| Item # | Checklist Item | Reported on Page # |
| --- | --- | --- |
|  |  |  |
| 1 | Identify the report as a systematic review incorporating a network meta-analysis (or related form of meta-analysis). | 1 |
| 2 | Provide a structured summary including, as applicable:  **Background:** main objectives  **Methods:** data sources; study eligibility criteria, participants, and interventions; study appraisal; and *synthesis methods, such as network meta-analysis.*  **Results:** number of studies and participants identified; summary estimates with corresponding confidence/credible intervals; treatment rankings may also be discussed. Authors may choose to summarize pairwise comparisons against a chosen treatment included in their analyses for brevity.  **Discussion/Conclusions:** limitations; conclusions and implications of findings.  **Other:** primary source of funding; systematic review registration number with registry name. | 1,2 |
| 3 | Describe the rationale for the review in the context of what is already known*, including mention of why a meta-analysis of randomized controlled trials and dose-response analysis has been conducted.* | 2-4 |
| 4 | Provide an explicit statement of questions being addressed, with reference to participants, interventions, comparisons, outcomes, and study design (PICOS). | 6 |
| 5 | Indicate whether a review protocol exists and if and where it can be accessed (e.g., Web address); and, if available, provide registration information, including registration number. | 5 |
| 6 | Specify study characteristics (e.g., PICOS, length of follow-up) and report characteristics (e.g., years considered, language, publication status) used as criteria for eligibility, giving rationale. *Clearly describe eligible treatments included in the treatment network, and note whether any have been clustered or merged into the same node (with justification).* | 6 |
| 7 | Describe all information sources (e.g., databases with dates of coverage, contact with study authors to identify additional studies) in the search and date last searched. | 5-6 |
| 8 | Present full electronic search strategy for at least one database, including any limits used, such that it could be repeated. | Supplementary 1 |
| 9 | State the process for selecting studies (i.e., screening, eligibility, included in systematic review, and, if applicable, included in the meta-analysis). | 6-7 |
| 10 | Describe method of data extraction from reports (e.g., piloted forms, independently, in duplicate) and any processes for obtaining and confirming data from investigators. | 6-7 |
| 11 | List and define all variables for which data were sought (e.g., PICOS, funding sources) and any assumptions and simplifications made. | 6-7 |
| S1 | Describe methods used to explore the geometry of the treatment network under study and potential biases related to it. This should include how the evidence base has been graphically summarized for presentation, and what characteristics were compiled and used to describe the evidence base to readers. | 7 |
| 12 | Describe methods used for assessing risk of bias of individual studies (including specification of whether this was done at the study or outcome level), and how this information is to be used in any data synthesis. | 7, Supplementary 3 |
| 13 | State the principal summary measures (e.g., risk ratio, difference in means). Also describe the use of additional summary measures assessed, such as treatment rankings and surface under the cumulative ranking curve (SUCRA) values, as well as modified approaches used to present summary findings from meta-analyses. | 8-10 |
| 14 | Describe the methods of handling data and combining results of studies for each network meta-analysis. This should include, but not be limited to:   - Handling of multi-arm trials; - Selection of variance structure; - Selection of prior distributions in Bayesian analyses; and - Assessment of model fit. | 8-10 |
| S2 | Describe the statistical methods used to evaluate the agreement of direct and indirect evidence in the treatment network(s) studied. Describe efforts taken to address its presence when found. | 8-10, Supplementary 5, Supplementary 7 |
| 15 | Specify any assessment of risk of bias that may affect the cumulative evidence (e.g., publication bias, selective reporting within studies). | 8-10,  Supplementary 4 |
| 16 | Describe methods of additional analyses if done, indicating which were pre-specified. This may include, but not be limited to, the following:   - Sensitivity or subgroup analyses; - Meta-regression analyses; - Alternative formulations of the treatment network; and - Use of alternative prior distributions for Bayesian analyses (if applicable). | 8-10,  Supplementary 6 |
| 17 | Give numbers of studies screened, assessed for eligibility, and included in the review, with reasons for exclusions at each stage, ideally with a flow diagram. | Figure 1 |
| S3 | Provide a network graph of the included studies to enable visualization of the geometry of the treatment network. | Figure 2, Figure 4 |
| S4 | Provide a brief overview of characteristics of the treatment network. This may include commentary on the abundance of trials and randomized patients for the different interventions and pairwise comparisons in the network, gaps of evidence in the treatment network, and potential biases reflected by the network structure. | 11-13 |
| 18 | For each study, present characteristics for which data were extracted (e.g., study size, PICOS, follow-up period) and provide the citations. | 10, Supplementary 2 |
| 19 | Present data on risk of bias of each study and, if available, any outcome level assessment. | 14, Supplementary 3, Supplementary 4 |
| 20 | For all outcomes considered (benefits or harms), present, for each study: 1) simple summary data for each intervention group, and 2) effect estimates and confidence intervals. *Modified approaches may be needed to deal with information from larger networks.* | 11-14, Supplementary 2 |
| 21 | Present results of each meta-analysis done, including confidence/credible intervals. In larger networks, authors may focus on comparisons versus a particular comparator (e.g. placebo or standard care), with full findings presented in an appendix. League tables and forest plots may be considered to summarize pairwise comparisons. If additional summary measures were explored (such as treatment rankings), these should also be presented. | 11-14, Figure 3, Figure 5, Supplementary 5 |
| S5 | Describe results from investigations of inconsistency. This may include such information as measures of model fit to compare consistency and inconsistency models, *P* values from statistical tests, or summary of inconsistency estimates from different parts of the treatment network. | Supplementary 5, Supplementary 7 |
| 22 | Present results of any assessment of risk of bias across studies for the evidence base being studied. | 14, Supplementary 3 |
| 23 | Give results of additional analyses, if done (e.g., sensitivity or subgroup analyses, meta-regression analyses*, alternative network geometries studied, alternative choice of prior distributions for Bayesian analyses,* and so forth). | 14-15, Supplementary 6 |
| 24 | Summarize the main findings, including the strength of evidence for each main outcome; consider their relevance to key groups (e.g., healthcare providers, users, and policy-makers). | 15 |
| 25 | Discuss limitations at study and outcome level (e.g., risk of bias), and at review level (e.g., incomplete retrieval of identified research, reporting bias). *Comment on the validity of the assumptions, such as transitivity and consistency. Comment on any concerns regarding network geometry (e.g., avoidance of certain comparisons).* | 20-21 |
| 26 | Provide a general interpretation of the results in the context of other evidence, and implications for future research. | 15-22 |
| 27 | Describe sources of funding for the systematic review and other support (e.g., supply of data); role of funders for the systematic review. This should also include information regarding whether funding has been received from manufacturers of treatments in the network and/or whether some of the authors are content experts with professional conflicts of interest that could affect use of treatments in the network. | 23 |

# PICOS = population, intervention, comparators, outcomes, study design.

# Supplementary 1.2: Search Strategy

| #24 | Search: ( "Roxadustat"[Mesh] OR "roxadustat"[Title/Abstract] OR "FG-4592"[Title/Abstract] OR "ASP1517"[Title/Abstract] OR "Daprodustat"[Mesh] OR "daprodustat"[Title/Abstract] OR "GSK1278863"[Title/Abstract] OR "Molidustat"[Mesh] OR "molidustat"[Title/Abstract] OR "BAY 85-3934"[Title/Abstract] OR "Vadadustat"[Mesh] OR "vadadustat"[Title/Abstract] OR "AKB-6548"[Title/Abstract] OR "Enarodustat"[Mesh] OR "enarodustat"[Title/Abstract] OR "JTZ-951"[Title/Abstract] OR "Desidustat"[Mesh] OR "desidustat"[Title/Abstract] OR "ZYAN1"[Title/Abstract] ) AND ( "Anemia"[Mesh] OR "Anemia, Renal"[Mesh] OR "Anemia, Iron-Deficiency"[Mesh] OR "Renal Insufficiency, Chronic"[Mesh] OR "Kidney Failure, Chronic"[Mesh] OR "renal anemia"[Title/Abstract] OR "kidney anemia"[Title/Abstract] OR "CKD anemia"[Title/Abstract] OR "anemia of chronic kidney disease"[Title/Abstract] OR "anemia in CKD"[Title/Abstract] OR "chronic kidney disease anemia"[Title/Abstract] ) AND ( "Randomized Controlled Trial"[Publication Type] OR "Controlled Clinical Trial"[Publication Type] OR "randomized"[Title/Abstract] OR "randomised"[Title/Abstract] OR "randomization"[Title/Abstract] OR "randomisation"[Title/Abstract] OR "placebo"[Title/Abstract] OR "trial"[Title/Abstract] OR "randomly"[Title/Abstract] OR "RCT"[Title/Abstract] OR "clinical trial"[Title/Abstract] OR "clinical trials as topic"[MeSH Terms] ) |
| --- | --- |
| #23 | Search: ( "Randomized Controlled Trial"[Publication Type] OR "Controlled Clinical Trial"[Publication Type] OR "randomized"[Title/Abstract] OR "randomised"[Title/Abstract] OR "randomization"[Title/Abstract] OR "randomisation"[Title/Abstract] OR "placebo"[Title/Abstract] OR "trial"[Title/Abstract] OR "randomly"[Title/Abstract] OR "RCT"[Title/Abstract] OR "clinical trial"[Title/Abstract] OR "clinical trials as topic"[MeSH Terms] ) |
| #22 | Search: ( "Anemia"[Mesh] OR "Anemia, Renal"[Mesh] OR "Anemia, Iron-Deficiency"[Mesh] OR "Renal Insufficiency, Chronic"[Mesh] OR "Kidney Failure, Chronic"[Mesh] OR "renal anemia"[Title/Abstract] OR "kidney anemia"[Title/Abstract] OR "CKD anemia"[Title/Abstract] OR "anemia of chronic kidney disease"[Title/Abstract] OR "anemia in CKD"[Title/Abstract] OR "chronic kidney disease anemia"[Title/Abstract] ) |
| #21 | Search: "renal anemia"[Title/Abstract] OR "kidney anemia"[Title/Abstract] OR "CKD anemia"[Title/Abstract] OR "anemia of chronic kidney disease"[Title/Abstract] OR "anemia in CKD"[Title/Abstract] OR "chronic kidney disease anemia"[Title/Abstract] |
| #20 | Search: "Kidney Failure, Chronic"[Mesh] |
| #19 | Search: "Renal Insufficiency, Chronic"[Mesh] |
| #18 | Search: "Anemia, Iron-Deficiency"[Mesh] |
| #17 | Search: "Anemia, Renal"[Mesh] |
| #16 | Search: "Anemia"[Mesh] |
| #15 | Search: "Desidustat"[Mesh] OR "desidustat"[Title/Abstract] OR "ZYAN1"[Title/Abstract] ) |
| #14 | Search: "desidustat"[Title/Abstract] OR "ZYAN1"[Title/Abstract] ) |
| #13 | Search: "Desidustat"[Mesh] |
| #12 | Search: "Enarodustat"[Mesh] OR "enarodustat"[Title/Abstract] OR "JTZ-951"[Title/Abstract] |
| #11 | Search: "enarodustat"[Title/Abstract] OR "JTZ-951"[Title/Abstract] |
| #10 | Search: "Enarodustat"[Mesh] |
| #9 | Search: "Vadadustat"[Mesh] OR "vadadustat"[Title/Abstract] OR "AKB-6548"[Title/Abstract] |
| #8 | Search: "vadadustat"[Title/Abstract] OR "AKB-6548"[Title/Abstract] |
| #7 | Search: "Vadadustat"[Mesh] |
| #6 | Search: "Molidustat"[Mesh] OR "molidustat"[Title/Abstract] OR "BAY 85-3934"[Title/Abstract] |
| #5 | Search: "molidustat"[Title/Abstract] OR "BAY 85-3934"[Title/Abstract] |
| #4 | Search: "Molidustat"[Mesh] |
| #3 | Search: "Roxadustat"[Mesh] OR "roxadustat"[Title/Abstract] OR "FG-4592"[Title/Abstract] OR "ASP1517"[Title/Abstract] OR "Daprodustat"[Mesh] OR "daprodustat"[Title/Abstract] OR "GSK1278863"[Title/Abstract] |
| #2 | Search: "roxadustat"[Title/Abstract] OR "FG-4592"[Title/Abstract] OR "ASP1517"[Title/Abstract] OR "Daprodustat"[Mesh] OR "daprodustat"[Title/Abstract] OR "GSK1278863"[Title/Abstract] |
| #1 | Search: "Roxadustat"[Mesh] |

# Supplementary 2.1: Characteristics of studies and subjects included in the review

| **Study** | **Country/**  **Region** | **Study design** | **Subjects  (intervention/ control)** | **Sex (male/female) (intervention/ control)** | **Mean age  (intervention/ control)** | **Baseline BMI (intervention/ control)** | **Population** | **Intervention detail** | | **Treatment Duration** | **Outcomes** | **Dialysis status** |
| --- | --- | --- | --- | --- | --- | --- | --- | --- | --- | --- | --- | --- |
|  |  |  |  |  |  |  |  | **Intervention group** | **Control group** |  |  |  |
| Agrawal 2022 | India | Open-label RCT | 588(294/294) | 147/147 vs. 149/145 | 53.38±13.9 3vs. 52.2±13.7 | 24.16±4.27 vs. 26.64±5.29 | Aged 18-80 years; CKD (Stages 3-5); Hb level within 7-10g/dL | desidustat (100 mg) TIW | darbepoetin alfa | 24 w | ①③⑤ | Non–dialysis |
| Akizawa 2017 | Japan | Double-blind RCT | 97 (78/19) | 38/40 vs. 12/7 | 62.2±9.69 vs. 63.4±8.98 | 21.85±3.31 vs. 20.3±2.70 | HD 3 times weekly for ≥ 8 weeks; Hb level within 9.5-12g/dL | daprodustat (4, 6, 8, or 10 mg) QD | placebo | 4 w | ①②③④⑤⑥ | Hemodialysis |
| Akizawa 2019a | Japan | Double-blind RCT | 94(71/23) | 39/32 vs. 9/14 | 68.5±11.2 vs. 65.3±14.7 | NA | Aged ≥20 years; Hb level within 8.0-10.5 g/dL | enarodustat (2, 4, or 6 mg) QD | placebo | 6 w | ①②④⑤⑥ | Non–dialysis |
| Akizawa 2019b | Japan | Double-blind RCT | 82(60/22) | 45/15 vs. 15/7 | 62.3±10.0 vs. 60.7±13.0 | NA | Aged ≥20 years; HD or HDF 3 times per week for at least 12 weeks; Hb level within 9.5-12g/dL | enarodustat (2, 4, 6 mg) QD | placebo | 30 w | ①②④⑤⑥ | Hemodialysis |
| Akizawa 2019c | Japan | Double-blind RCT | 121(101/20) | 56/45 vs. 9/11 | 68.7±12 vs. 67.1±15.9 | NA | D1: Anemia associated with CKD; not receiving dialysis | molidustat (25, 50, or 75 mg QD, or 25 or 50 mg BID) | placebo | 16 w | ①②③④⑤ | Non–dialysis  Non–dialysis  Hemodialysis |
|  |  | Open-label RCT | 124(92/32) | 45/47 vs. 18/14 | 67.6±10.5 vs. 68.8±8.7 | NA | D2: Anemia associated with CKD; previously treated with ESA; not receiving dialysis | molidustat (25, 50, or 75 mg) QD | darbepoetin | 16 w | ①②③④⑤ |  |
|  |  | Open-label RCT | 199(157/42) | 91/66 vs. 29/13 | 59.4±12.5 vs. 58.9±9.1 | NA | D4: Anemia associated with CKD; previously treated with ESA; receiving regular HD | Molidustat (25, 50, 75, or 150 mg) QD | epoetin | 16 w | ①②③④⑤ |  |
| Akizawa 2019d | Multinational | Open-label RCT | 160(118/42) | 47/61 vs. 22/20 | 70±10 vs. 69±12 | NA | D3: patients who either experienced a stopping event in D1 or completed the treatment period in D1 or D2 | molidustat (individually optimized dose) | darbepoetin | 144 w | ①⑥ | Non–dialysis  Hemodialysis |
|  |  | Open-label RCT | 87(57/30) | 33/24 vs. 23/7 | 61±12 vs. 59±9 | NA | D5: patients who completed the end-of-treatment visit in D4. | molidustat (15, 25, 50, 75, 100, and 150 mg) QD | epoetin | 144 w | ①⑥ |  |
| Akizawa 2019e | Japan | Double-blind RCT | 107(80/27) | 39/41 vs. 11/16 | 64.4±8.7 vs. 61.9±10.6 | NA | Aged 20-74 years; anemia associated with NDD-CKD; Hb levels ≤10 g/dL | roxadustat (50, 70,or 100 mg) TIW | placebo | 24 w | ①②④⑤⑥ | Non–dialysis |
| Akizawa 2020a | Japan | Open-label RCT | 237 (74/163) | 55/19 vs.98/65 | 66.2±12.2 vs. 66.2±12.1 | 23.36±3.50 vs.23.36 ±3.64 | maintenance hemodialysis | Roxadustat, 50 or 70 mg TIW, 24 weeks | ESA TIW, 24weeks | 24 w | ①②③④⑤⑥ | Hemodialysis |
| Akizawa 2020b | Japan | Double-blind RCT | 301(150/151) | 101/49 vs. 107/44 | 64.6±11.7 vs. 64.9±10.1 | NA | Aged ≥20 years; HD three times weekly for 12 weeks; Hb level within 10-12 g/dL | roxadustat (70 or 100 mg) TIW | darbepoetin alfa | 24 w | ①②③⑤⑥ | Hemodialysis |
| Akizawa 2021a | Japan | Double-blind RCT | 172(86/86) | 61/25 vs. 61/25 | 63.2±10.8 vs. 64.8±10.3 | 61/25 vs. 61/25 | Aged ≥20 years; HD 3 times a week at least 12 weeks; Hb level within 9.5-12g/dL | enarodustat 4 mg/d as the initial dose | darbepoetin alfa | 24 w | ①②③④⑤⑥ | Hemodialysis |
| Akizawa 2021b | Japan | Double-blind RCT | 229(153/76) | 91/62 vs. 49/27 | 66.2±10.3 vs. 64.8±10.6 | 22.61±3.34 vs. 22.25±3.12 | Aged ≥20 years; HD or HDF 3 times per week for at least 12 weeks; Hb level within 9.5-12g/dL | molidustat 75 mg/d as the initial dose | darbepoetin alfa | 52 w | ①⑥ | Hemodialysis |
| Barratt 2021 | Multinational | Open-label RCT | 616(323/293) | 145/178 vs. 129/164 | 66.8±13.6 vs. 65.7±14.4 | 27.95±5.76 vs. 28.74±6.06 | Aged ≥18 years; CKD (Stages 3-5); Hb levels ≤10.5 g/dL | roxadustat (70 or 100 mg) TIW | darbepoetin alfa | 104 w | ①②③⑤⑥ | Non–dialysis |
| Besarab 2015 | USA | single-blind RCT | 116(88/28) | 33/55 vs. 16/12 | 64 vs. 68.6 | NA | Aged 18−80 years; CKD (Stages 3-4) | Roxadustat (1, 1.5, 2 and 0.7 mg/kg) BIW or TIW | placebo | 4 w | ②③⑤⑥ | Non–dialysis |
| Charytan 2021 | USA | Open-label RCT | 741(370/371) | 187/183 vs. 215/156 | 57.6±13.6 vs. 58.4±13.3 | 30.2±7.4 vs. 30.5±7.5 | Aged ≥18 years; dialysis for ≥3 months before screening; Hb level within 9-12g/dL | Roxadustat (70, 100, 150, or 200 mg) TIW | epoetin alfa | 52 w | ①②③④⑤ | Mixed peritoneal–hemodialysis |
| Chen 2017 | China | Double-blind RCT | 91(61/30) | 18/43 vs. 8/22 | 48.8±13.8 vs. 51.4±11.9 | NA | Aged 18−80 years; CKD (Stages 3-5); Hb levels≤10 g/dL | roxadustat (1.1-1.75 or 1.5-2.25 mg/kg) TIW | placebo | 8 w | ①②③④⑤⑥ | Non–dialysis  Hemodialysis |
|  |  | Open-label RCT | 96(74/22) | 45/29 vs. 13/9 | 49.9±12.8 vs. 53.8±10 | NA | Aged 18−80 years; received dialysis; Hb level within 9-12g/dL | roxadustat (1.1-1.8, 1.5-2.3, or 1.7-2.3 mg/kg) TIW | epoetin alfa | 6 w | ①②③④⑤⑥ |  |
| Chen 2019a | China | Double-blind RCT | 152(101/51) | 36/65 vs. 20/31 | 54.7±13.3 vs. 53.2±13.1 | NA | Aged 18−75 years; CKD (Stages 3-5); Hb level within 7-10g/dl | roxadustat (70 or 100mg) TIW | placebo | 9 w | ①②③⑤⑥ | Non–dialysis |
| Chen 2019b | China | Open-label RCT | 304(204/100) | 126/78 vs. 58/42 | 47.6±11.7 vs. 51.0±11.8 | NA | Aged 18−75 years; dialysis for at least 16 weeks; Hb level within 9-12g/dl | roxadustat 100mg or 120 mg as the starting dose | epoetin alfa | 26 w | ①②③⑤⑥ | Mixed peritoneal–hemodialysis |
| Chertow 2021a | Multinational | Open-label RCT | 1751(879/872) | 404/475 vs. 366/506 | 65.2±14.27 vs. 64.9±13.71 | NA | Aged ≥18 years; Hb levels≤10 g/dL; had not received previous treatment with ESAs | vadadustat (300mg as the starting dose) | darbepoetin alfa | 208 w | ①⑥ | Non–dialysis |
| Chertow 2021b | Multinational | Open-label RCT | 1725(862/863) | 394/468 vs. 375/488 | 67.3±13.14 vs. 66.5±13.52 | NA | Aged ≥18 years; Hb levels≤10 g/dL; had treated with ESAs | vadadustat (300mg as the starting dose) | darbepoetin alfa | 208 w | ①⑥ | Non–dialysis |
| Coyne 2021 | Multinational | Double-blind RCT | 922(616/306) | 241/375 vs. 130/176 | 64.9±12.6 vs. 64.8±13.2 | 27.4±6.3 vs. 27.3±6.0 | Aged ≥18 years; CKD (Stages 3-5); Hb levels≤10g/dL | roxadustat (70 or 100 mg) TIW | placebo | 52 w | ①②③④⑤ | Non–dialysis |
| Coyne 2022 | USA | Double-blind RCT | 407(270/137) | 149/121 vs. 81/56 | 60 vs. 56 | NA | HD 3 times per week for at least 90 days; Hb level within 8-11.5g/dL | daprodustat (2-48 mg) TIW | epoetin alfa | 52 w | ①②③④⑤ | Hemodialysis |
| Csiky 2021 | Multinational | Open-label RCT | 834(414/420) | 245/169 vs. 235/185 | 61.0±13.8 vs. 61.8±13.4 | 26.87±4.86 vs. 26.95±5.59 | Aged ≥18 years; HD/PD for at least 4 months; Hb level within 9.5-12g/dL | Roxadustat (20, 50, or 100 mg) TIW | ESA | 52 w | ①⑥ | Mixed peritoneal–hemodialysis |
| Eckardt 2021a | Multinational | Open-label RCT | 369(181/188) | 107/74 vs. 113/75 | 56.5±14.8 vs. 55.6±14.6 | NA | Aged ≥18 years; undergoing dialysis;Hb level within 8-11g/dl | vadadustat 300mg as the starting dose | darbepoetin alfa | 176 w | ①⑥ | Mixed peritoneal–hemodialysis |
| Eckardt 2021b | Multinational | Open-label RCT | 3554(1777/1777) | 990/787 vs. 1004/773 | 57.9±13.9 vs. 58.4±13.8 | NA | Aged ≥18 years; undergoing dialysis;Hb level within 8-11g/dl | vadadustat 300mg as the starting dose | darbepoetin alfa | 176 w | ① | Mixed peritoneal–hemodialysis |
| Fishbane 2021 | Multinational | Double-blind RCT | 2761(1384/1377) | 564/820 vs. 603/774 | 60.9±14.7 vs. 62.4±14.1 | 26.7±6.0 vs. 26.9±6.1 | Aged ≥18 years; CKD (Stages 3-5); Hb level < 10g/dL | roxadustat (70 mg as the starting dose) TIW | placebo | 52 w | ②③ | Non–dialysis |
| Fishbane 2022 | Multinational | Open-label RCT | 2106(1051/1055) | 625/426 vs. 626/429 | 53.5±15.3 vs. 54.5±15.0 | 27.0±6.8 vs. 26.9±6.4 | Aged ≥18 years; HD or PD for ≥30 days before randomization; Hb level within 10-12g/dL | Roxadustat (70-200 mg as the starting dose ) TIW | epoetin alfa | 164 w | ②③⑤⑥ | Mixed peritoneal–hemodialysis |
| Gang 2022 | India | Open-label RCT | 392(196/196) | 135/61 vs. 134/62 | 51.02±13.97 vs. 50.91±13.48 | 22.42±4.96 vs. 22.90±5.01 | Aged ≥18 years; on dialysis (≥2 times in a week) for at least 12 weeks; baseline Hb level within 8-11g/dL | desidustat (100 mg) TIW | epoetin alfa | 24 w | ①④⑤ | Mixed peritoneal–hemodialysis |
| Holdstock 2016 | Multinational | Double-blind RCT | 72(54/18) | 16/38 vs. 4/14 | 68.3±11.5 vs. 69.2±11 | 32.0±8.2 vs. 32.1±9.6 | Aged ≥18 years; CKD (Stages 3-5); not on dialysis and were not taking rhEPO; Hb level within 8.5-11g/dL | daprodustat (0.5, 2, 5 mg) QD | placebo | 4 w | 1. ③④⑤ | Non–dialysis  Hemodialysis |
|  |  | Double-blind RCT | 82(62/20) | 43/19 vs. 16/4 | 55.7±17.7 vs. 64.2±12.8 | 29.5±7.8 vs. 29.4±7.7 | Aged ≥18 years; HD 3 times weekly for at least 8 weeks, and were using rhEPO; Hb level within 9.5-12.0 g/dL | daprodustat (0.5, 2, 5 mg) QD | rhEPO | 4 w | 1. ②③④⑤ |  |
| Hou 2022 | China | Open-label RCT | 129(86/43) | 47/39 vs. 25/18 | 48±12 vs. 48.3±13 | 23.3±3.6 vs. 24.1±3.2 | Patients diagnosed with CKD and renal anemia; Hb levels ≤12 g/dL | roxadustat (100 or 120 mg) TIW | ESAs | 24 w | ②③⑤⑥ | Peritoneal dialysis |
| Johansen 2023 | Multinational | Double-blind RCT | 614(307/307) | 131/176 vs. 129/178 | 66.0 vs. 67.0 | NA | Aged ≥18 years; CKD (Stages 3-5); Hb level within 8.5-10.5 g/dL | Daprodustat (2 or 4 mg) QD | placebo | 28 w | ①②③④⑤⑥ | Non–dialysis |
| Martin 2017 | USA | Double-blind RCT | 91(72/19) | 36/36 vs. 7/12 | 65.7±9.6 vs. 64.9±10.0 | 30.4±5.8 vs. 29.8±6.6 | Aged 18−79 years; CKD (Stages 3-4); Hb levels≤10.5 g/dL | vadadustat (240, 370, 500, or 630 mg) QD | placebo | 6 w | ①②④ | Non–dialysis |
| Meadowcroft 2019 | USA | Open-label RCT | 210(171/39) | 108/63 vs. 26/13 | 59.6±13.3 vs. 59.7±18.7 | 27.7±7.5 vs. 27.2±5.8 | HD 3-5 times weekly; Hb level within 9-11.5g/dL | daprodustat (4, 6, 8, 10 or 12 mg) QD | placebo | 24 w | ①②③④⑤ | Hemodialysis |
| Nangaku 2021 | Multinational | Open-label RCT | 217(108/109) | 66/42 vs. 69/40 | 69±11 vs. 71±9 | 23±3.3 vs. 24±3.7 | CKD (Stages 3-5); Hb level within 8-11 g/dL(ESA-naïve) or 9-13 g/dL(ESA users) | daprodustat (2-4 mg) QD | epoetin beta pegol | 52 w | ①②③④⑤⑥ | Non–dialysis |
| Nangaku 2022 | Japan | Open-label RCT | 299 (149/150) | 96/53 vs. 92/58 | 68±11.6 vs. 70±9.1 | 23.2±3.43 vs. 24.2±4.15 | Aged ≥20 years; Hb level within 8-11 g/dL(ESA-naïve) or 9-13 g/dL(ESA users) | daprodustat (1-24 mg) QD | ESA | 52 w | ⑥ | Non–dialysis |
|  |  | Double-blind RCT | 355(220/135) | 160/60 vs. 89/46 | 64±10.0 vs. 64±10.5 | 23.2±3.43 vs. 23.0±3.69 | Aged ≥20 years; HD or PD; Hb level within 8-10 g/dL | daprodustat (4 mg) QD | ESA | 52 w | ⑥ | Mixed peritoneal–hemodialysis |
| Parmar 2019 | India | Double-blind RCT | 117(87/30) | 33/54 vs. 17/13 | 48.48±12.1 vs. 46.9±12.66 | NA | Aged 18-65 years; CKD (Stages 1-4); Hb level within 6.5-11g/dL | desidustat (100, 150, or 200 mg) TIW | placebo | 6 w | ③④ | Non–dialysis |
| Pergola 2016 | USA | Double-blind RCT | 210(138/72) | 57/81 vs. 38/34 | 66.6±9.97 vs. 65.9±12.33 | 31.9±6.56 vs. 30.0±7.27 | Aged ≥20 years; CKD (Stages 3-5) | vadadustat (150-600 mg) QD | placebo | 20 w | 1. ④⑥ | Non–dialysis |
| Provenzano 2016 | USA | Open-label RCT | 90(67/23) | 45/22 vs. 14/9 | 56.9±12.1  vs. 57±11.6 | NA | Aged 18-75 years; HD 3 times a week for 4 or more months. Hb levels were 9.0-13.5 g/dL | roxadustat (1.0, 1.5, 1.8, or 2.0 mg/kg) TIW | epoetin alfa | 19 w | ①②③④⑤⑥ | Hemodialysis |
| Provenzano 2021 | Multinational | Open-label RCT | 1043(522/521) | 309/213 vs. 307/214 | 53.8±14.7 vs. 54.3±14.6 | NA | Aged ≥18 years; HD or PD for 2 weeks to ≤ 4 months; Hb levels ≤10g/dl. | roxadusta (70 or 100 mg) TIW | epoetin alfa | 52 w | ①②③④⑤ | Mixed peritoneal–hemodialysis |
| Shutov 2021 | Multinational | Double-blind RCT | 594(391/203) | 169/222 vs. 99/104 | 62.0 vs. 63.0 | NA | Aged ≥18 years; diagnosed with CKD stage 3-5; Hb levels ≤10g/dL | roxadusta (70 or 100 mg) TIW | placebo | 104 w | ①⑥ | Non–dialysis |
| Singh 2021 | Multinational | Open-label RCT | 3872(1937/1935) | 835/1102 vs. 864/1071 | 67 vs. 67 | NA | CKD (Stages 3-5) | daprodustat (1-4 mg) QD | darbepoetin alfa | 148 w | ①⑥ | Non–dialysis |
| Singh 2022 | Multinational | Open-label RCT | 312(157/155) | 96/61 vs. 98/57 | 52 vs. 56 | NA | Started and received HD or PD within 90 days before randomization; Hb levels were 8-10.5g/dL | daprodustat (2mg) QD | darbepoetin alfa | 52 w | ①⑥ | Mixed peritoneal–hemodialysis |
| Toka 2025 | USA | Open-label, sponsor-blind RCT | 456(304/152) | 176/128 vs. 66/86 | 60.7±13.74 vs. 61.6±12.80 | 31.2±7.6 vs. 31.8±7.7 | Aged ≥18 years; on dialysis (TIW) for at least 12 weeks; baseline Hb level within 8.5-11g/dL | vadadustat (600 or 900 mg) TIW | MPG-EPO | 26 w | ①⑥ | Hemodialysis |
| Wu 2024 | China | Open-label RCT | 25(13/12) | 4/9 vs. 7/5 | 57.3±12.09 vs. 52.8±12.64 | NA | Aged ≥18 years; on dialysis (TIW) | roxadusta (100 or 120 mg) TIW | rHuEPO | 2 w | ②③④⑤⑥ | Mixed peritoneal–hemodialysis |
| Yamamoto 2021a | Japan | Open-label RCT | 162(82/80) | 50/32 vs. 50/30 | 72.1±9.3 vs. 71.2±10.1 | NA | Aged ≥20 years; CKD (Stages 3-5); Hb levels were 8-11g/dL | molidustat 25 mg QD at a starting dose | darbepoetin | 52 w | ①⑥ | Non–dialysis |
| Yamamoto 2021b | Japan | Open-label RCT | 164(82/82) | 45/37 vs. 54/28 | 69.0±10.3 vs. 72.4±10.3 | NA | Aged ≥20 years; CKD (Stages 3-5);Hb levels were 10-13g/dL | molidustat 25 or 75mg QD at a starting dose | darbepoetin | 52 w | ①⑥ | Non–dialysis |
| Zhang 2023 | China | Open-label RCT | 159(106/53) | 59/47 vs. 26/27 | 47.59±13.63 vs. 50.15±12.02 | 23.58±3.66 vs. 24.80±4.01 | Aged 18-75; Received PD > 6 weeks; Hb levels ofwere 60–100 g/L | Roxadustat (70, 100, or 120 mg) TIW | ESAs | 24 w | ①④⑤ | Peritoneal dialysis |

Note: RCT, randomized controlled trial; QD, once daily; TIW, three times weekly; BID, twice daily; BIW, twice weekly; HD, hemodialysis; HDF, hemodiafiltration; PD, peritoneal dialysis; ESA, erythropoiesis-stimulating agent; CKD, chronic kidney disease; NDD-CKD, non–dialysis-dependent CKD; BMI, body mass index; Hb, hemoglobin; w, weeks; m, months; NA, not applicable; ① hemoglobin; ② ferritin; ③ serum iron; ④ hepcidin; ⑤ TSAT; ⑥ AE.

**Supplementary 2.2: Studies with imputed or estimated standard deviations (SDs)**

| **First Author**  **(Year)** | **Intervention(s)** | **Comparator(s)** | **Outcome(s) requiring SD estimation** | **Method of SD estimation** | **Notes** |
| --- | --- | --- | --- | --- | --- |
| Akizawa 2019a | Enarodustat | Placebo | Hb, TSAT | SD derived from standard error (SE) using Cochrane formulas | Short trial (6 weeks), SE reported |
| Akizawa 2019b | Enarodustat | Placebo | Hb | SD derived from confidence intervals (CIs) | 30-week dialysis trial |
| Akizawa 2019c | Molidustat | Placebo | Serum iron | SD estimated from reported ranges (Hozo et al., 2005 method) | Non-dialysis arm |
| Chen 2017 | Roxadustat | Placebo | Hb, ferritin | SD derived from p-values (Cochrane formulas) | Small sample, 8 weeks |
| Chen 2019a | Roxadustat | Placebo | Hb | SD imputed based on similar roxadustat RCTs with comparable population | Non-dialysis patients |
| Parmar 2019 | Desidustat | Placebo | hepcidin | SD derived from ranges (Hozo et al., 2005 method) | Non-dialysis CKD |
| Provenzano 2016 | Roxadustat | Epoetin alfa | Hb, TSAT | SD imputed from related studies with same intervention class | Hemodialysis patients |

Notes: Hb = hemoglobin; TSAT = transferrin saturation; SD = standard deviation.

SDs were estimated following the Cochrane Handbook recommendations: (i) conversion from SE, CI, or p-values; (ii) estimation from ranges (Hozo et al., 2005); (iii) imputation from comparable studies when no direct data were available.

A total of 7 studies required SD estimation, as listed in this table.

# Supplementary 3: Risk of Bias

| **Author** | **Bias arising from the randomization process** | **Bias due to deviations from intended intervention** | **Bias due to missing outcome data** | **Bias in measurement of the outcome** | **Bias in selection of the reported result** | **Overall** |
| --- | --- | --- | --- | --- | --- | --- |
| Agrawal2022 | Low | Low | Low | Low | Low | Low |
| Akizawa2017 | Low | Low | Low | Low | Low | Low |
| Akizawa2019a | Low | Low | Low | Some Concerns | Low | Some Concerns |
| Akizawa2019b | Some Concerns | Low | Low | Low | Low | Some Concerns |
| Akizawa2019c | Low | Low | Low | Low | Low | Low |
| Akizawa2019d | Low | Low | Low | Low | Low | Low |
| Akizawa2019e | Low | Low | Some Concerns | Low | Low | Some Concerns |
| Akizawa2020a | Low | Low | Low | Low | Low | Low |
| Akizawa2020b | Some Concerns | Low | Some Concerns | Low | Low | Some Concerns |
| Akizawa2021a | Low | Low | Low | Low | Low | Low |
| Akizawa2021b | Low | Low | Low | Low | Low | Low |
| Barratt2021 | Some Concerns | Low | Low | Low | Low | Some Concerns |
| Besarab2015 | Low | Low | Low | Low | Low | Low |
| Charytan2021 | High | High | High | Low | Low | High |
| Chen2017 | Low | Low | Low | Low | Low | Low |
| Chen2019a | Low | Low | Some Concerns | Low | Low | Some Concerns |
| Chen2019b | Some Concerns | Low | Some Concerns | Low | Low | Some Concerns |
| Chertow 2021a | Low | High | High | Low | Low | High |
| Chertow 2021b | Low | Low | Low | Low | Low | Low |
| Coyne2021 | Some Concerns | Some Concerns | Some Concerns | Low | Low | Some Concerns |
| Coyne2022 | Low | Some Concerns | Low | Low | Low | Some Concerns |
| Csiky2021 | Low | Low | Low | Low | Low | Low |
| Eckardt2021a | Low | Low | Some Concerns | Low | Low | Some Concerns |
| Eckardt2021b | Low | Low | Low | Low | Low | Low |
| Fishbane2021 | Some Concerns | Low | Low | Low | Low | Some Concerns |
| Fishbane2022 | High | Some Concerns | Some Concerns | Low | Low | High |
| Gang2022 | Low | Low | Some Concerns | Low | Low | Some Concerns |
| Holdstock2016 | Low | Low | Low | Low | Low | Low |
| Hou2022 | Low | Low | Low | Low | Low | Low |
| Johansen2023 | Low | Low | Low | Low | Low | Low |
| Martin2017 | Low | Some Concerns | Low | Low | Low | Some Concerns |
| Meadowcroft2019 | Some Concerns | Low | High | High | Low | High |
| Nangaku2021 | Some Concerns | Low | Some Concerns | Low | Low | Some Concerns |
| Nangaku2022 | Low | Low | Low | Low | Low | Low |
| Parmar2019 | Low | Low | Low | Some Concerns | Low | Some Concerns |
| Pergola2016 | Some Concerns | Low | Some Concerns | Low | Low | Some Concerns |
| Provenzano2016 | Low | Low | Some Concerns | Low | Low | Some Concerns |
| Provenzano2021 | Low | Low | Some Concerns | Low | Low | Some Concerns |
| Shutov2021 | Low | Low | Low | Low | Low | Low |
| Singh2021 | Low | Low | Low | Low | Low | Low |
| Singh2022 | Low | Low | Low | Low | Low | Low |
| Toka2025 | Low | Low | Low | Low | Low | Low |
| Wu2024 | Low | Low | Low | Low | Low | Low |
| Yamamoto2021a | Low | Low | Low | Low | Low | Low |
| Yamamoto2021b | Low | Some Concerns | Low | Low | Low | Some Concerns |
| Zhang2023 | Low | High | Low | Low | Low | High |

# Supplementary 4: Publication bias


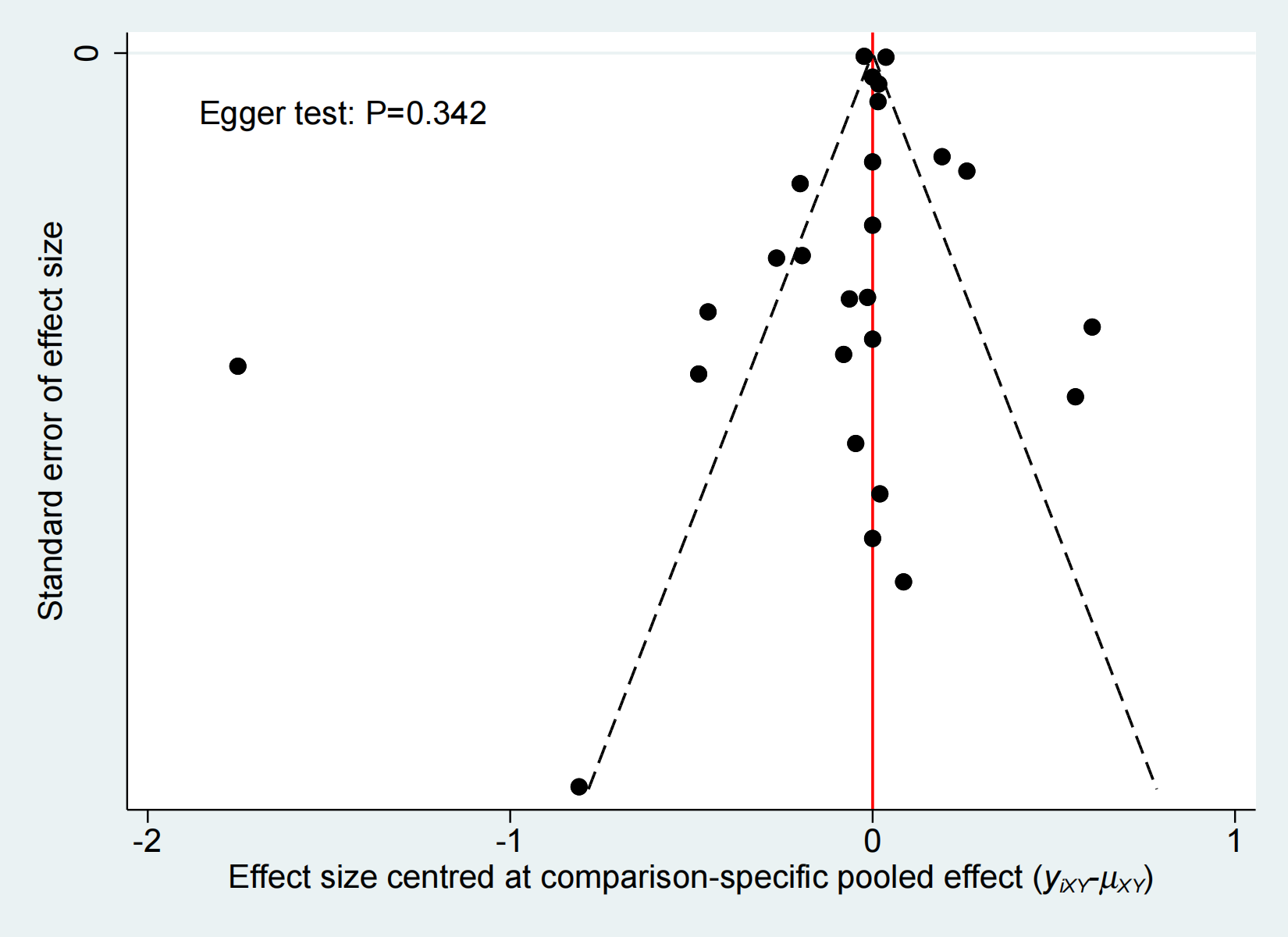


Figure 4.1. Funnel plot of hemoglobin in non-dialysis patients (Egger’s test: p = 0.342).


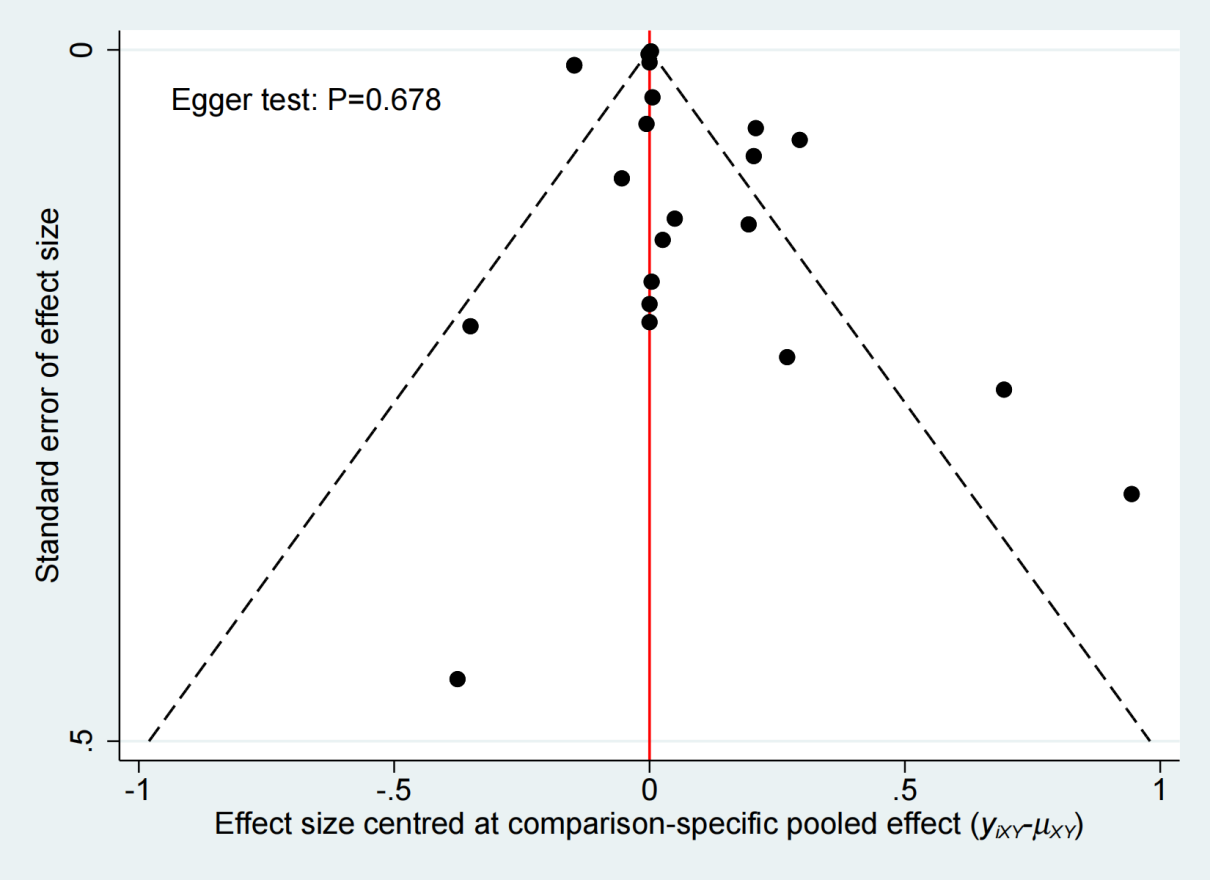


Figure 4.2. Funnel plot of hemoglobin in dialysis patients (Egger’s test: p = 0.678).


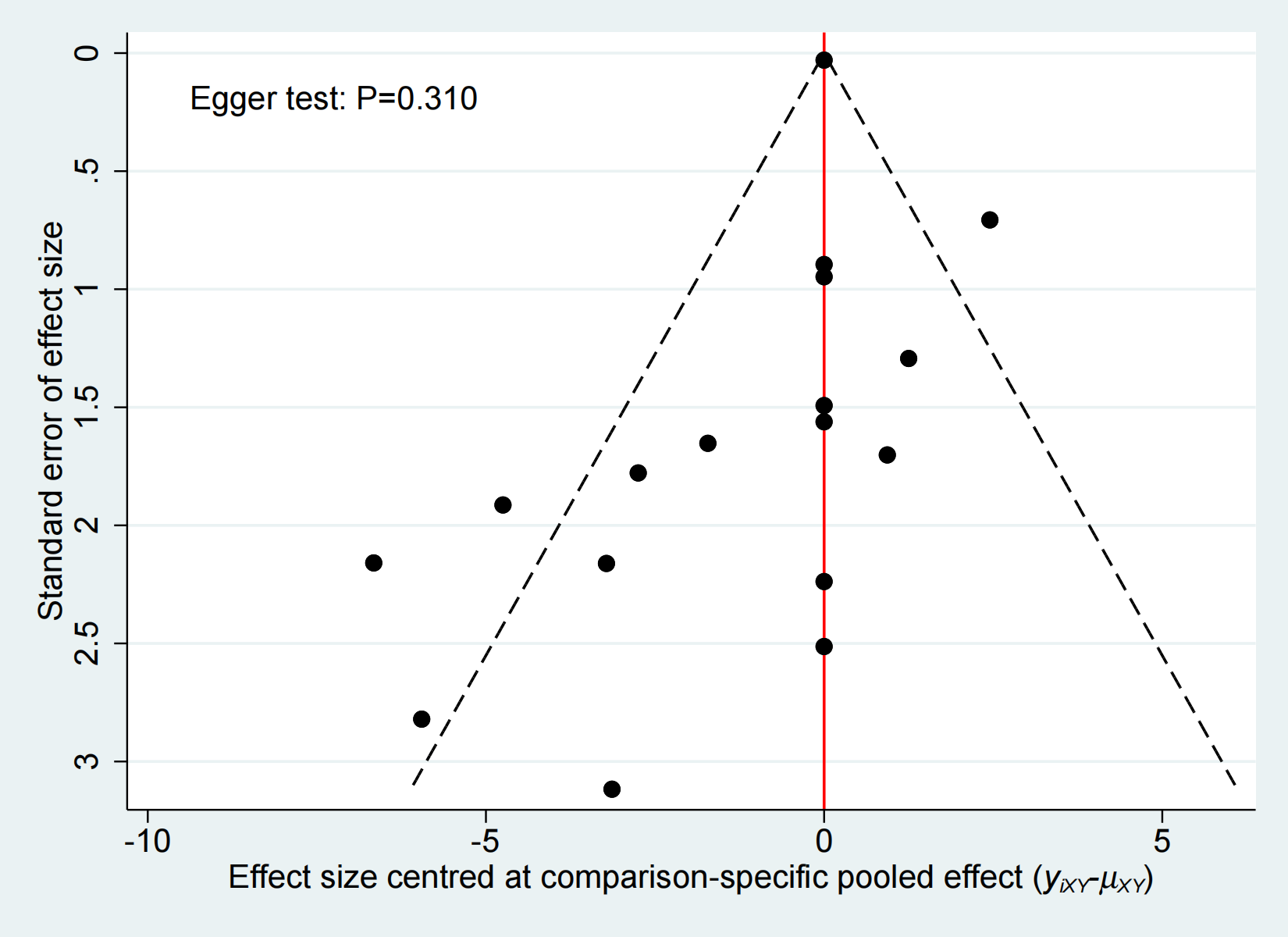


Figure 4.3. Funnel plot of TSAT in non-dialysis patients (Egger’s test: p = 0.310).


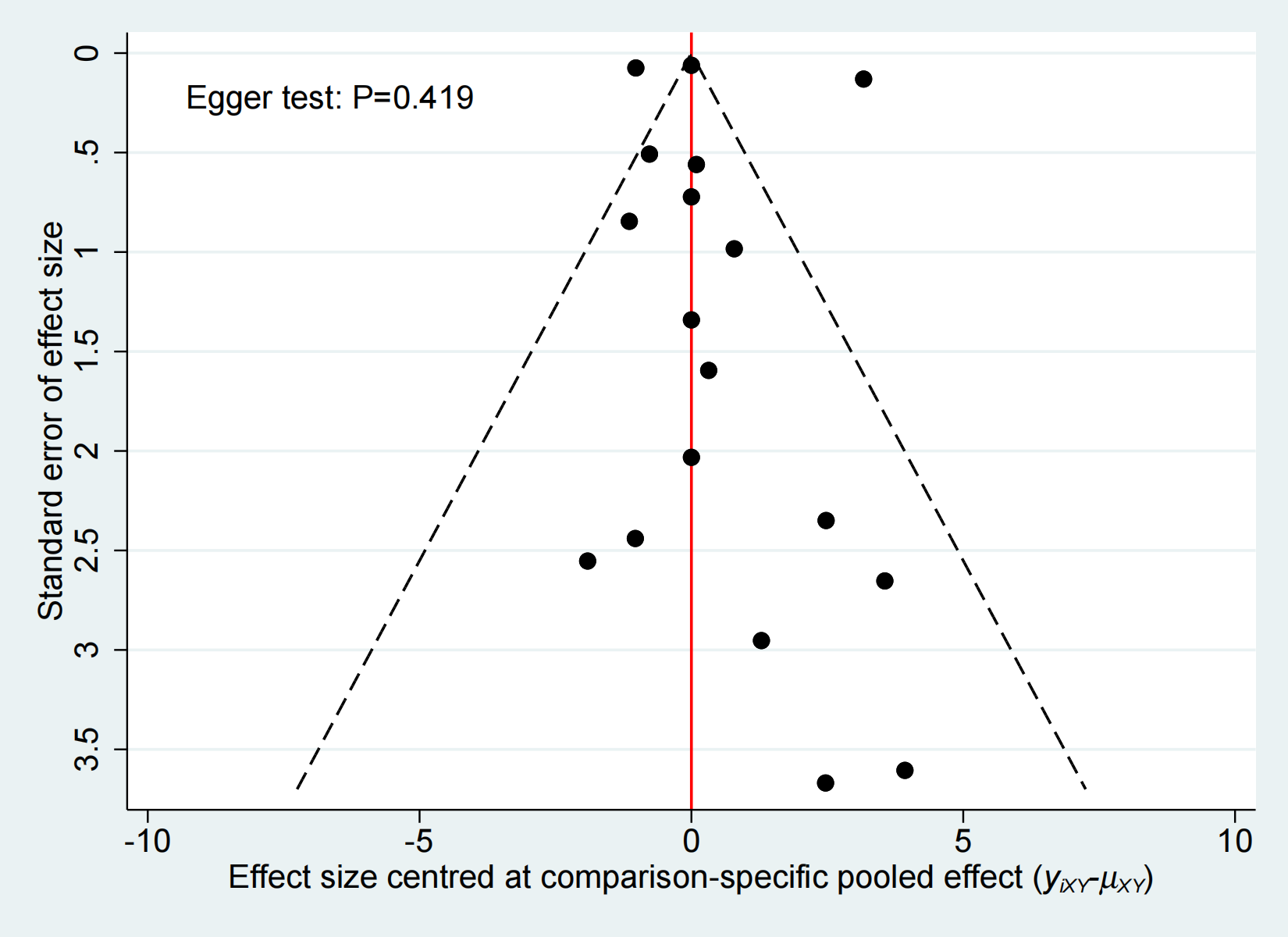


Figure 4.4. Funnel plot of TSAT in dialysis patients (Egger’s test: p = 0.419).


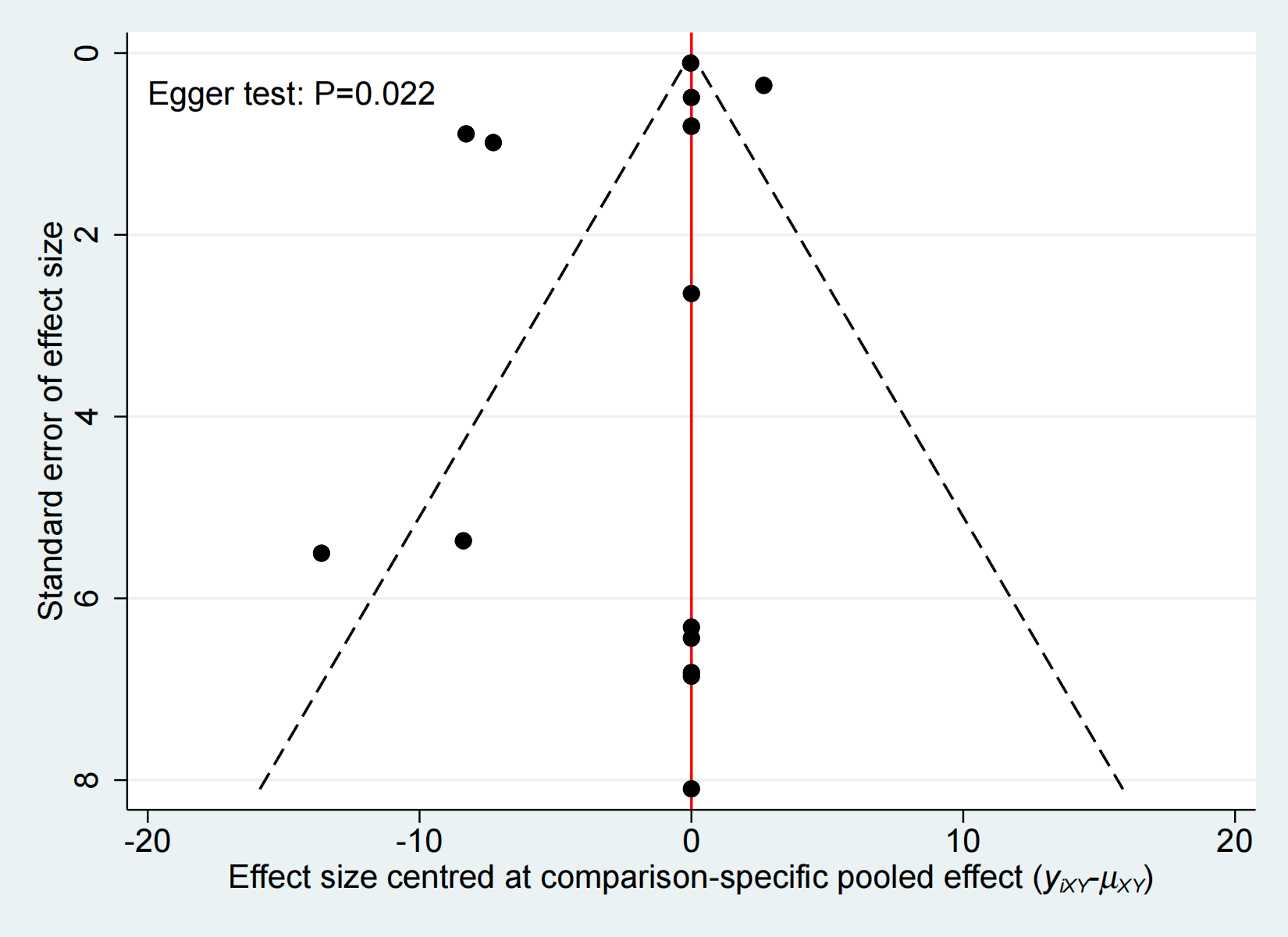


Figure 4.5. Funnel plot of Serum Iron in non-dialysis patients (Egger’s test: p = 0.022).


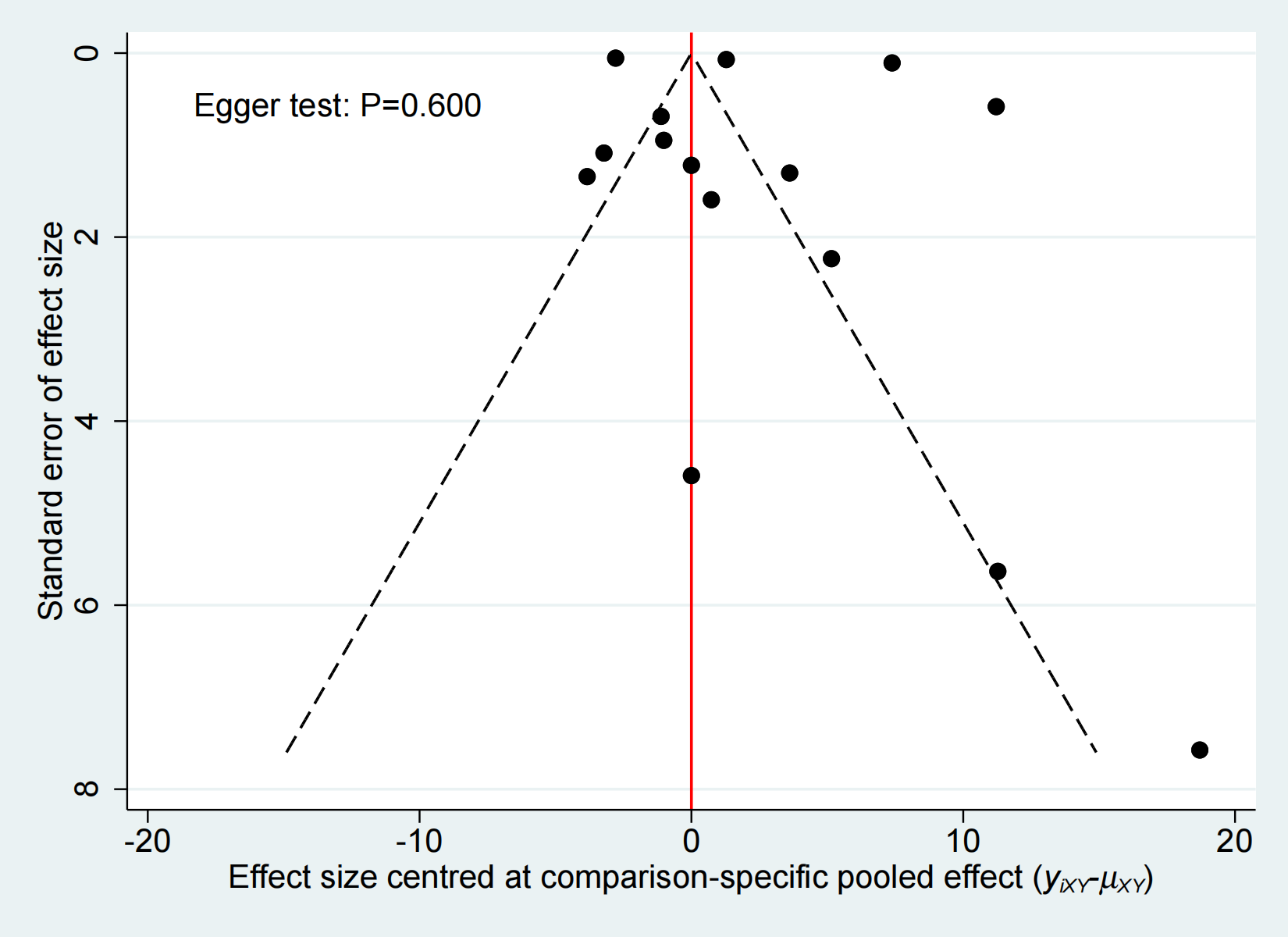


Figure 4.6. Funnel plot of Serum Iron in dialysis patients (Egger’s test: p = 0.600).


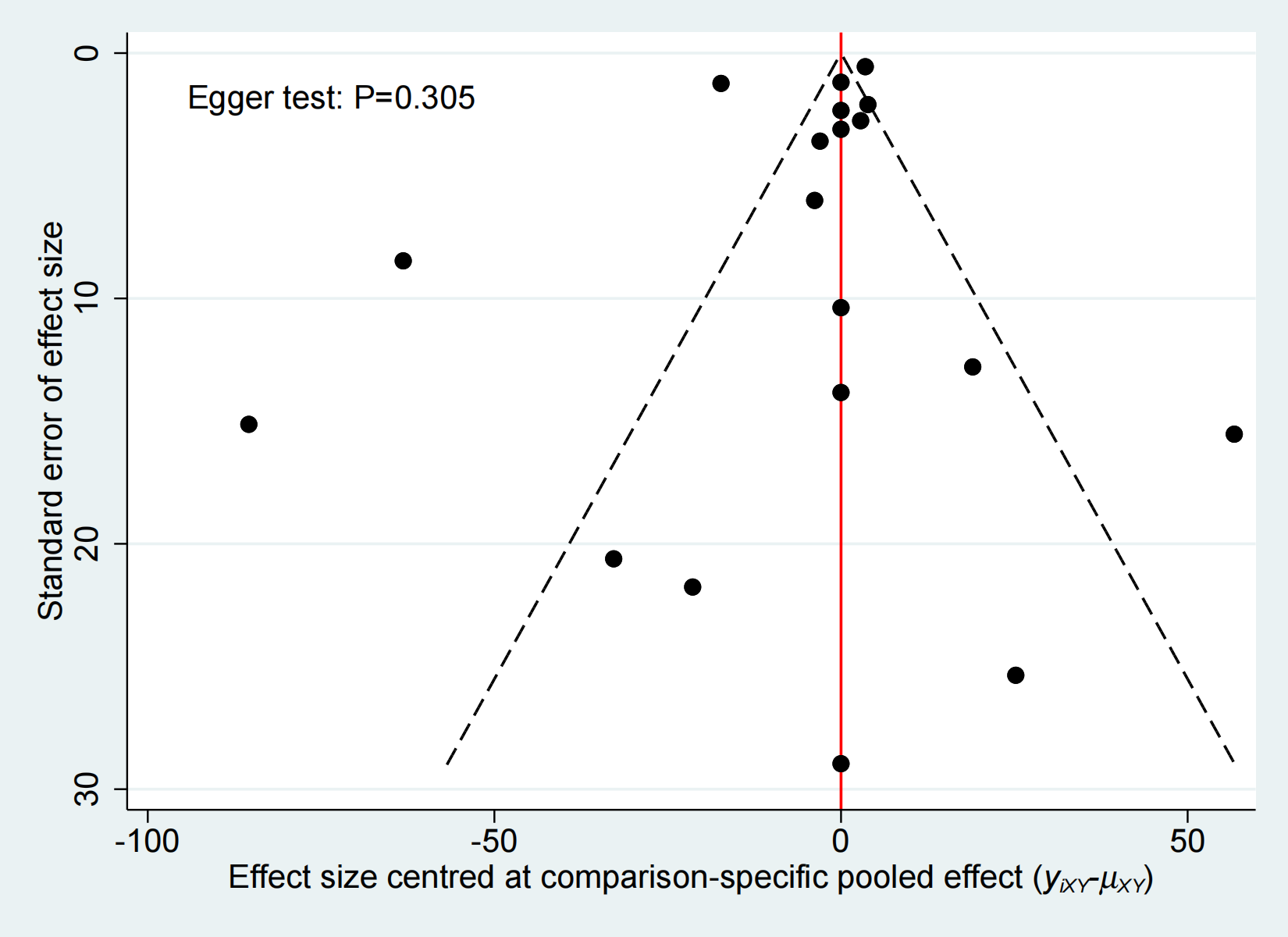


Figure 4.7. Funnel plot of Ferritin in non-dialysis patients (Egger’s test: p = 0.305).


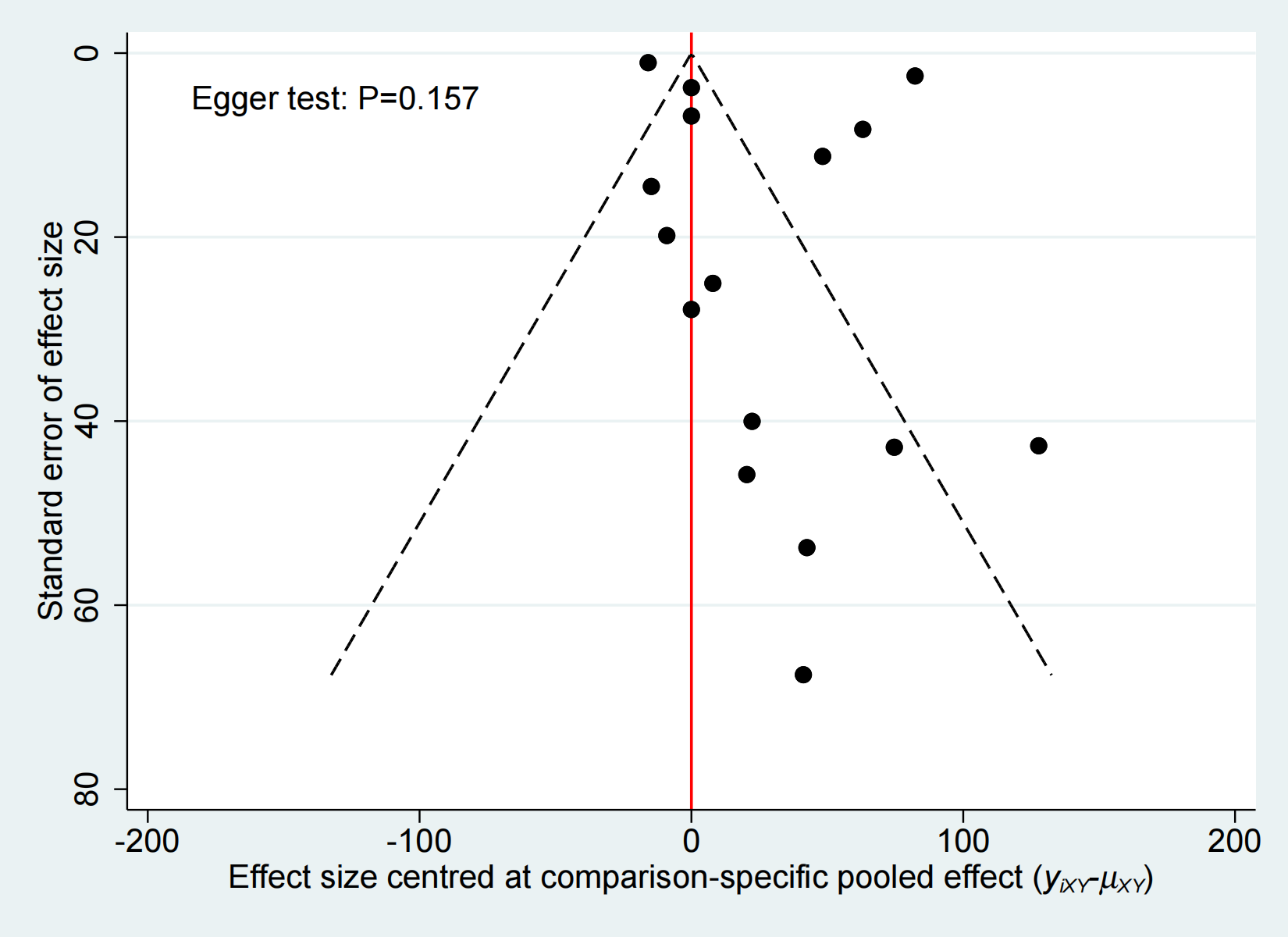


Figure 4.8. Funnel plot of Ferritin in dialysis patients (Egger’s test: p = 0.157).


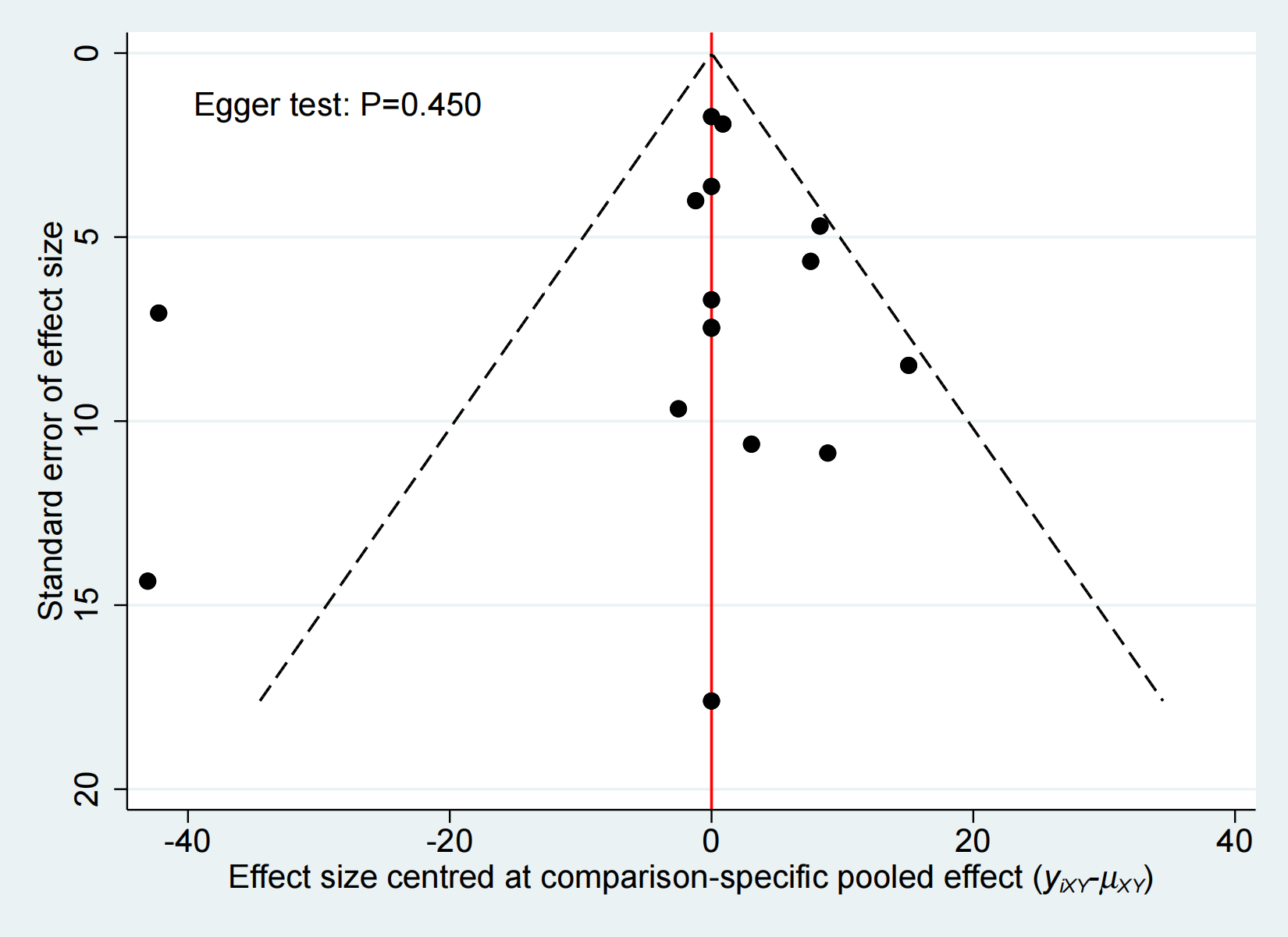


Figure 4.9. Funnel plot of Hepcidin in non-dialysis patients (Egger’s test: p = 0.450).


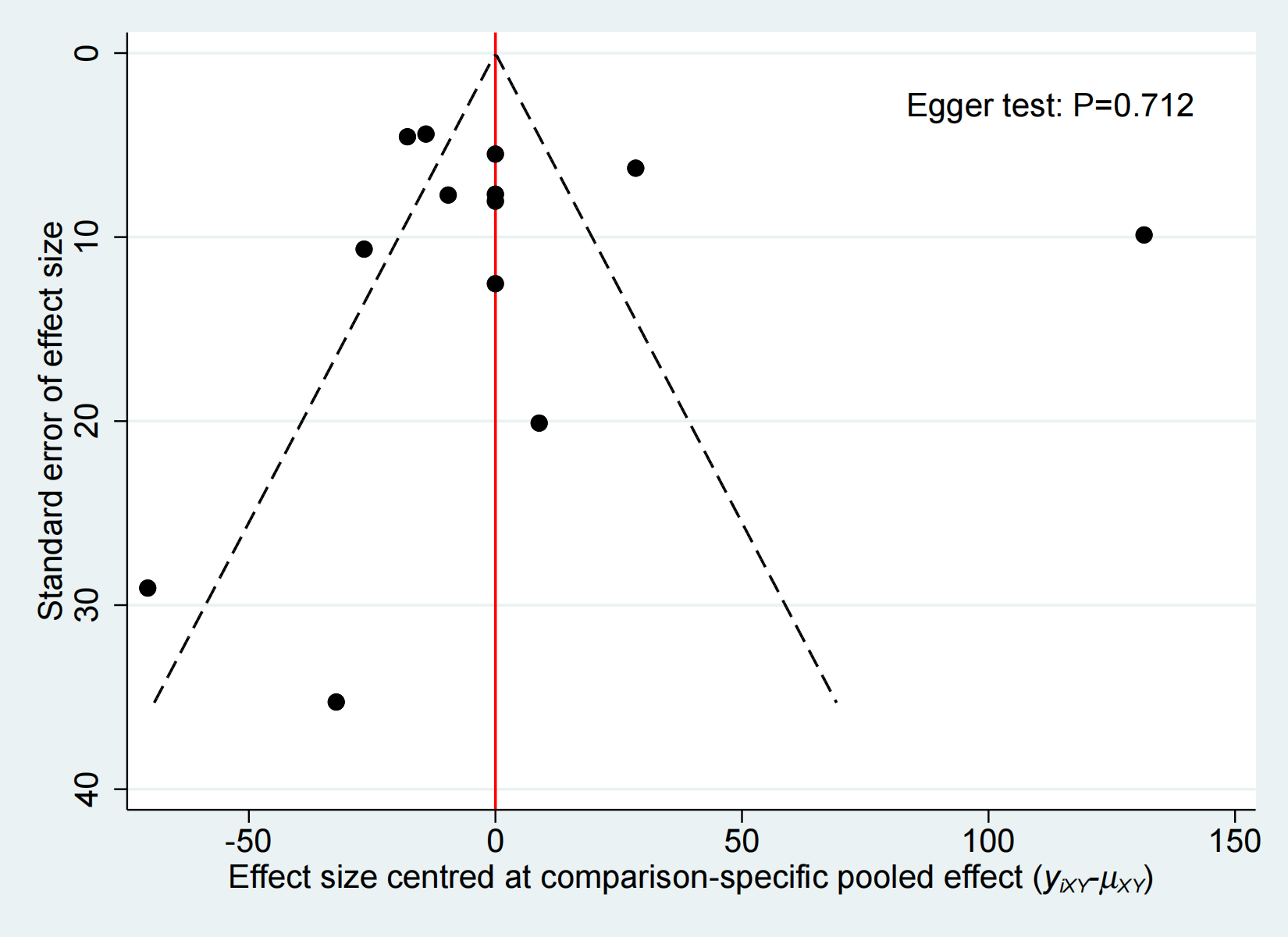


Figure 4.10. Funnel plot of Hepcidin in dialysis patients (Egger’s test: p = 0.712).


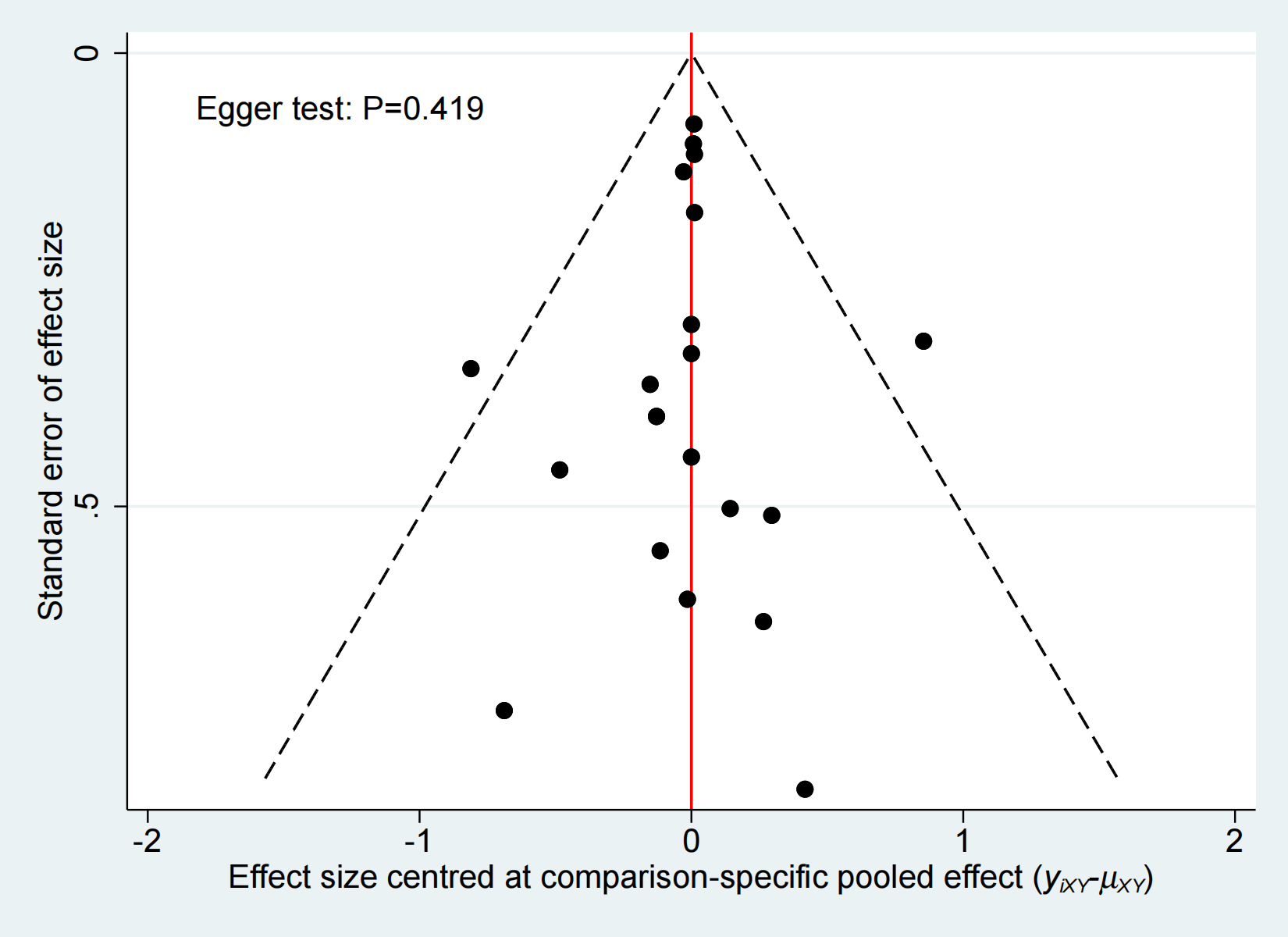


Figure 4.11. Funnel plot of AE in non-dialysis patients (Egger’s test: p = 0.419).


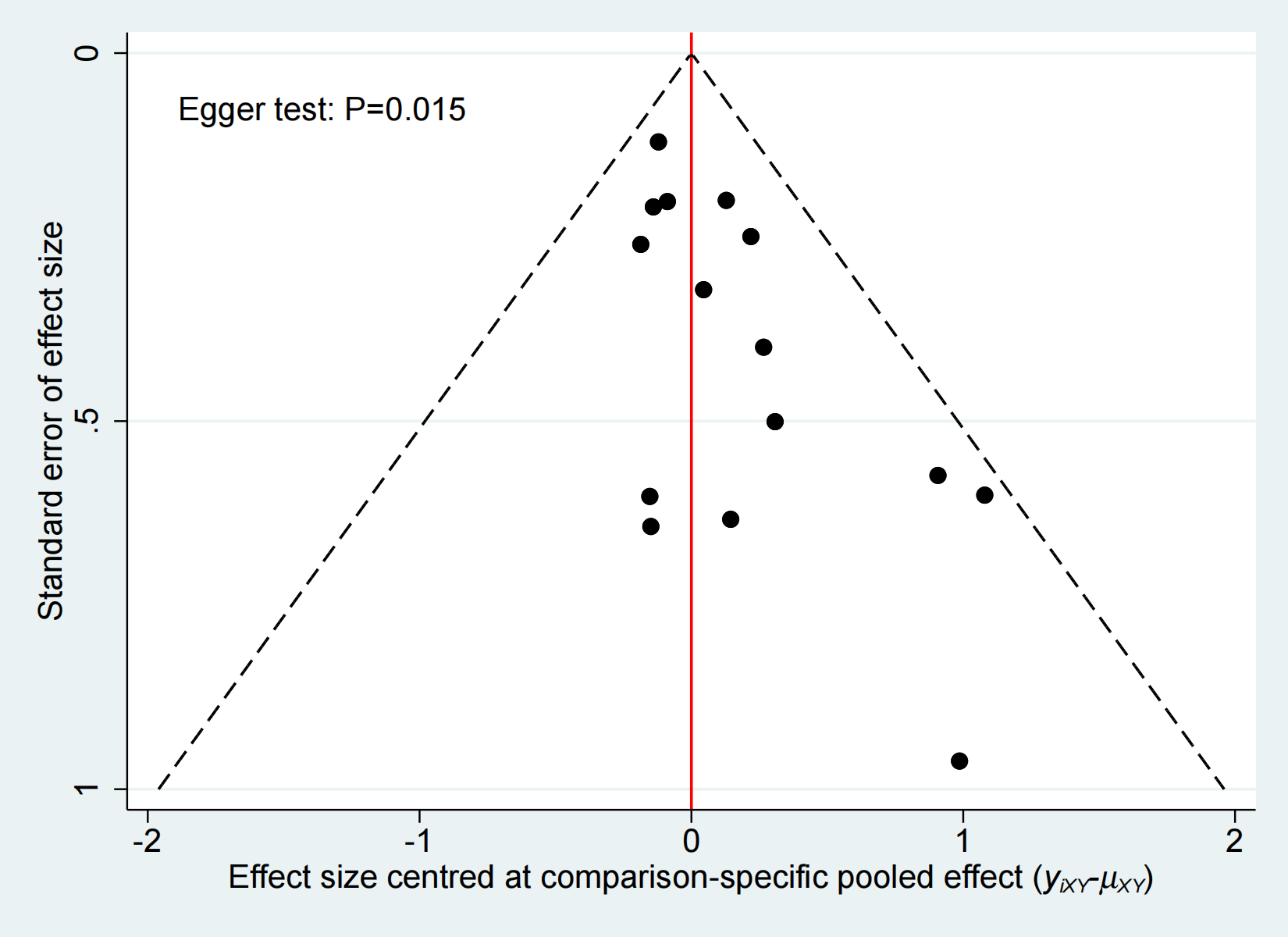


Figure 4.12. Funnel plot of AE in dialysis patients (Egger’s test: p = 0.015).

# Supplementary 5: Forest plot


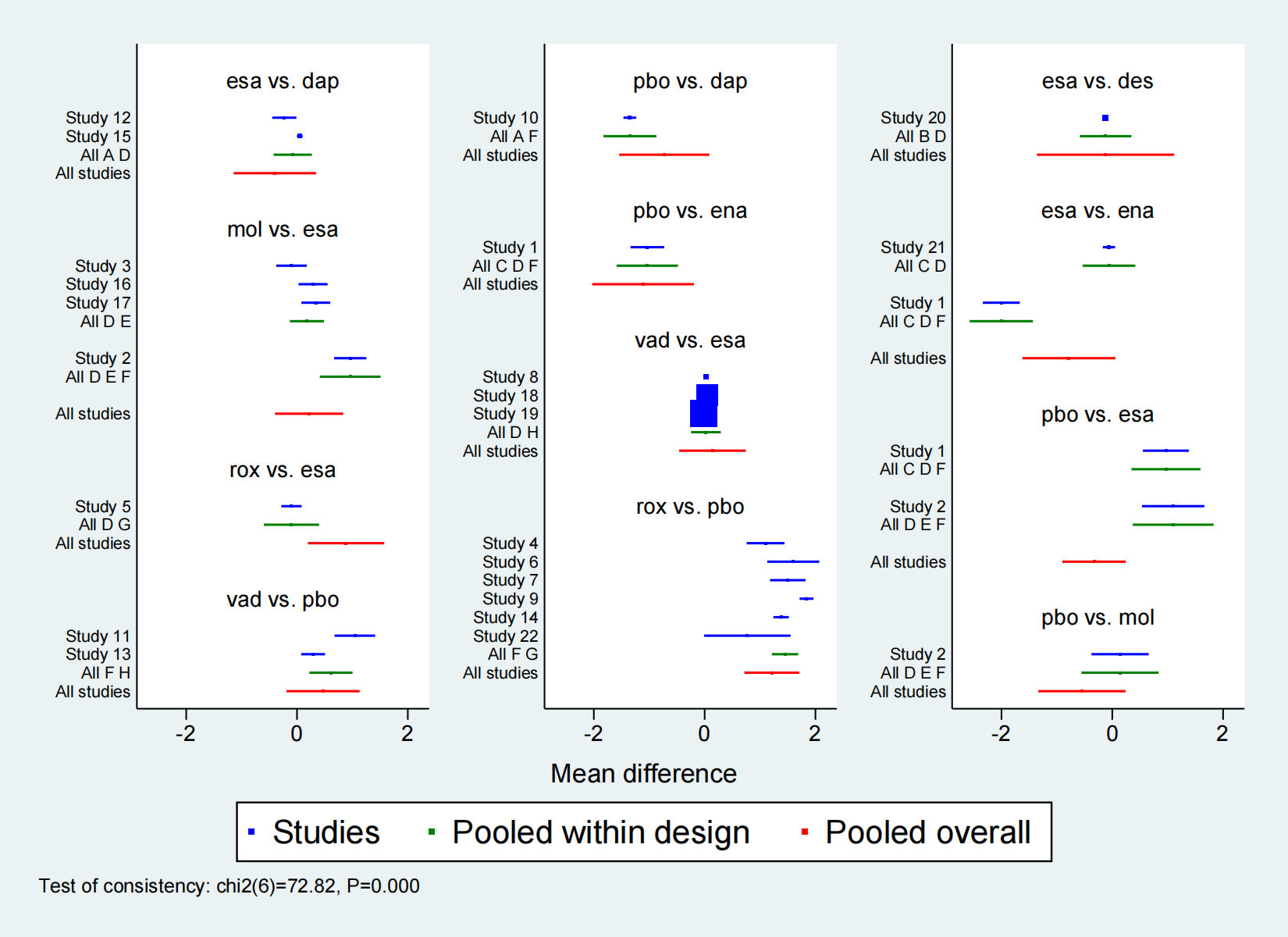


Figure 5.1. Forest plot of hemoglobin in non-dialysis patients (Consistency’s test: p = 0.000).


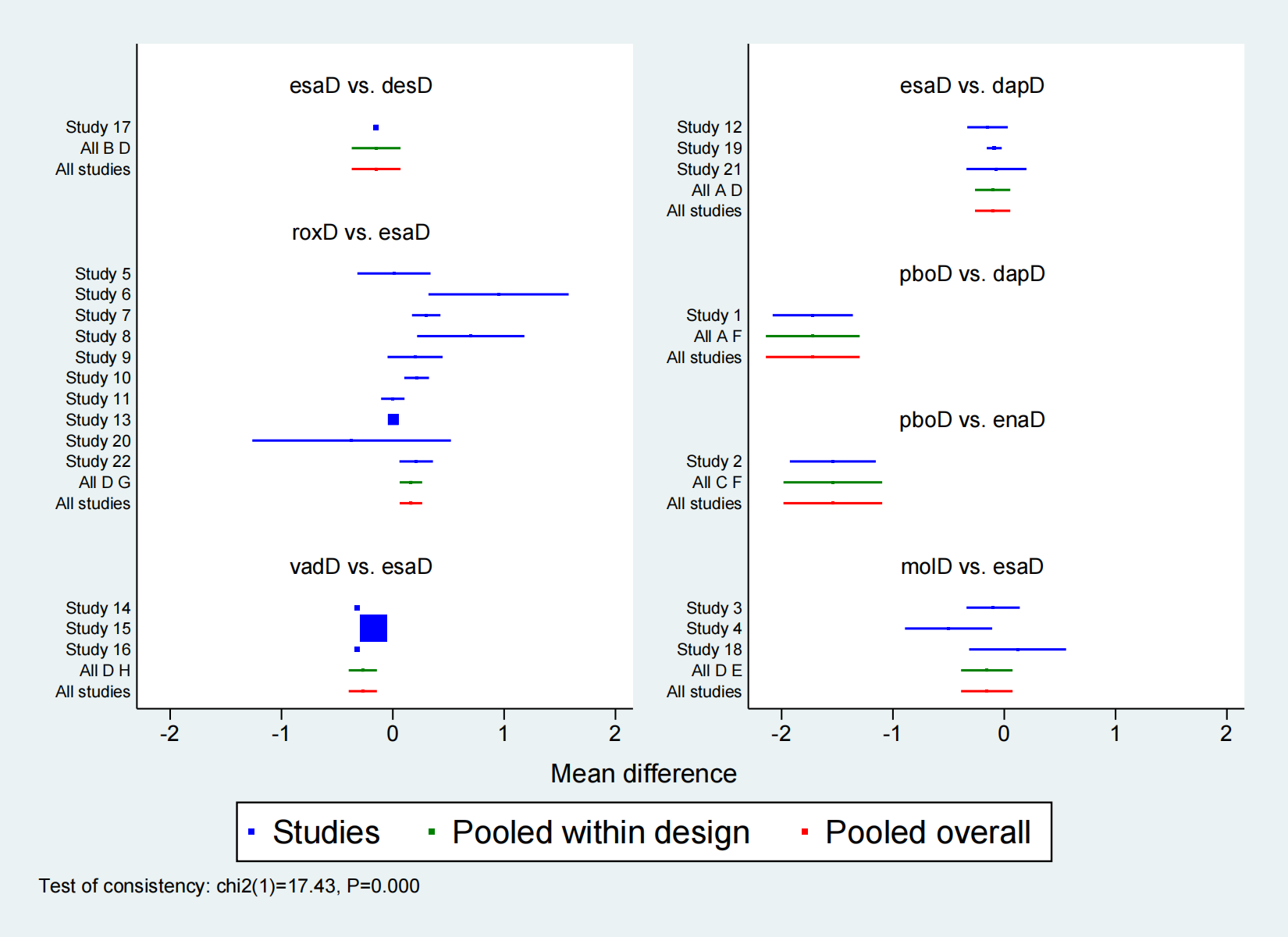


Figure 5.2. Forest plot of hemoglobin in dialysis patients (Consistency’s test: p = 0.000).


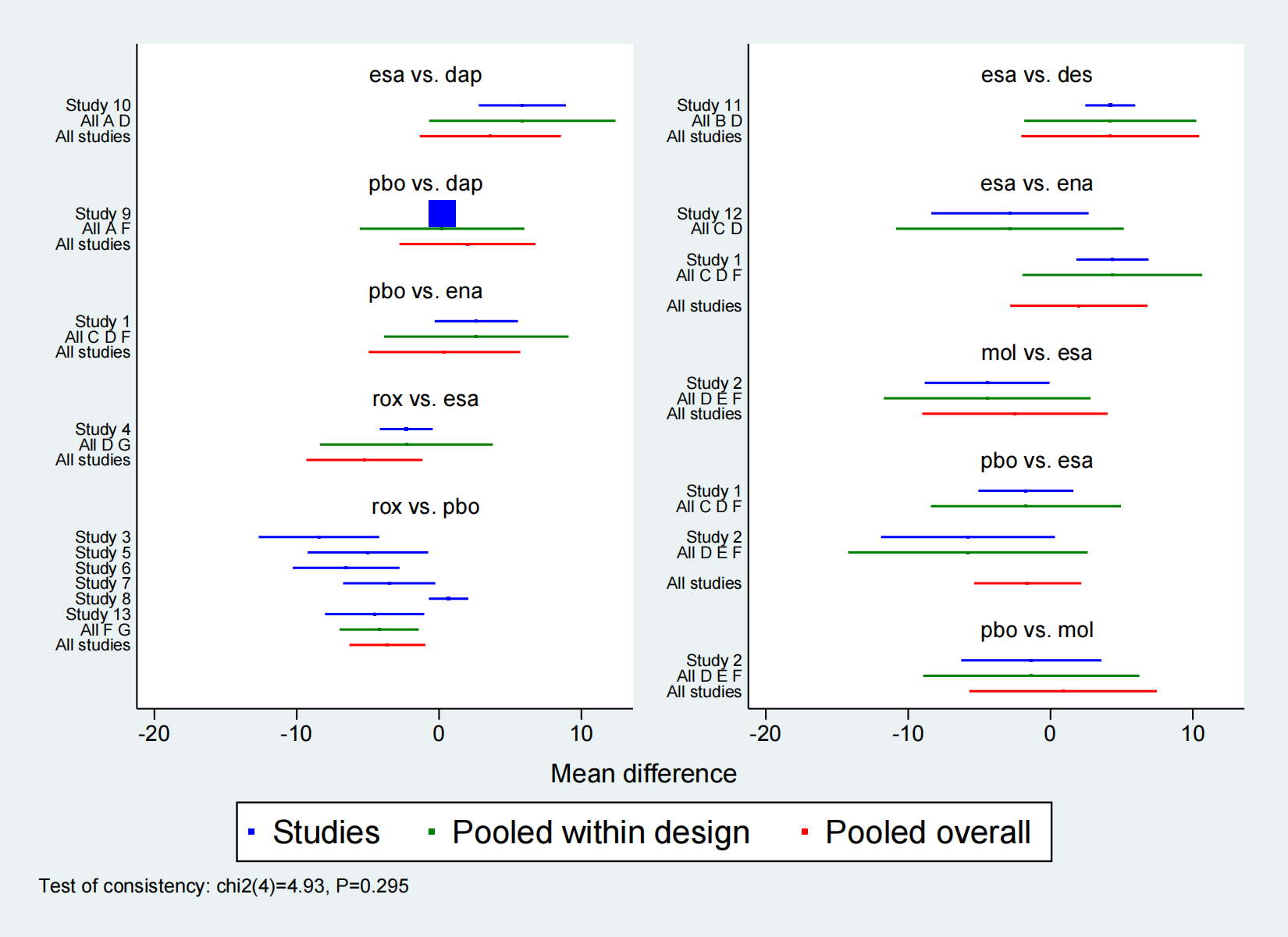


Figure 5.3. Forest plot of TSAT in non-dialysis patients (Consistency’s test: p = 0.295).


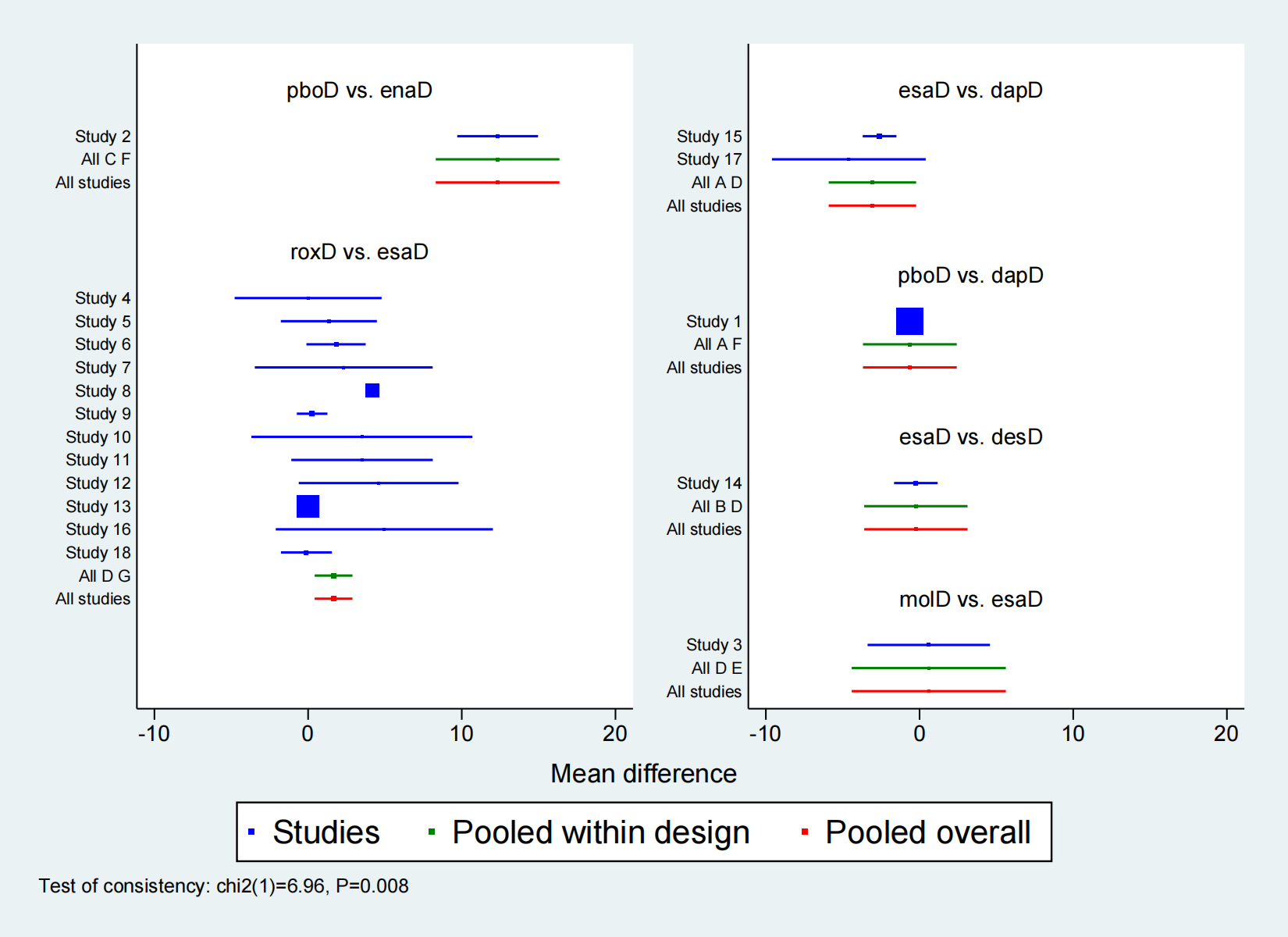


Figure 5.4. Forest plot of TSAT in dialysis patients (Consistency’s test: p = 0.008).


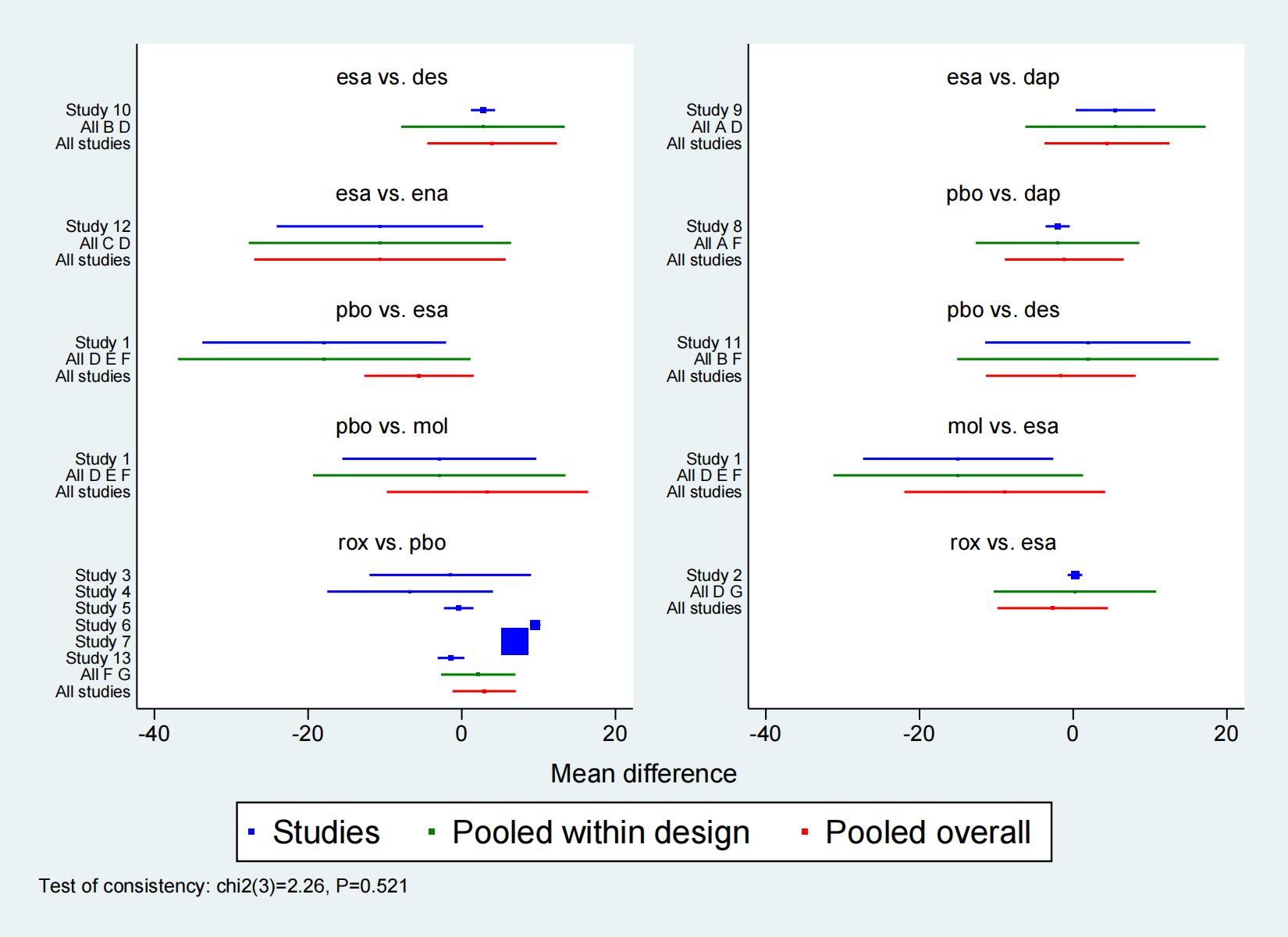


Figure 5.5. Forest plot of Serum Iron in non-dialysis patients (Consistency’s test: p = 0.521).


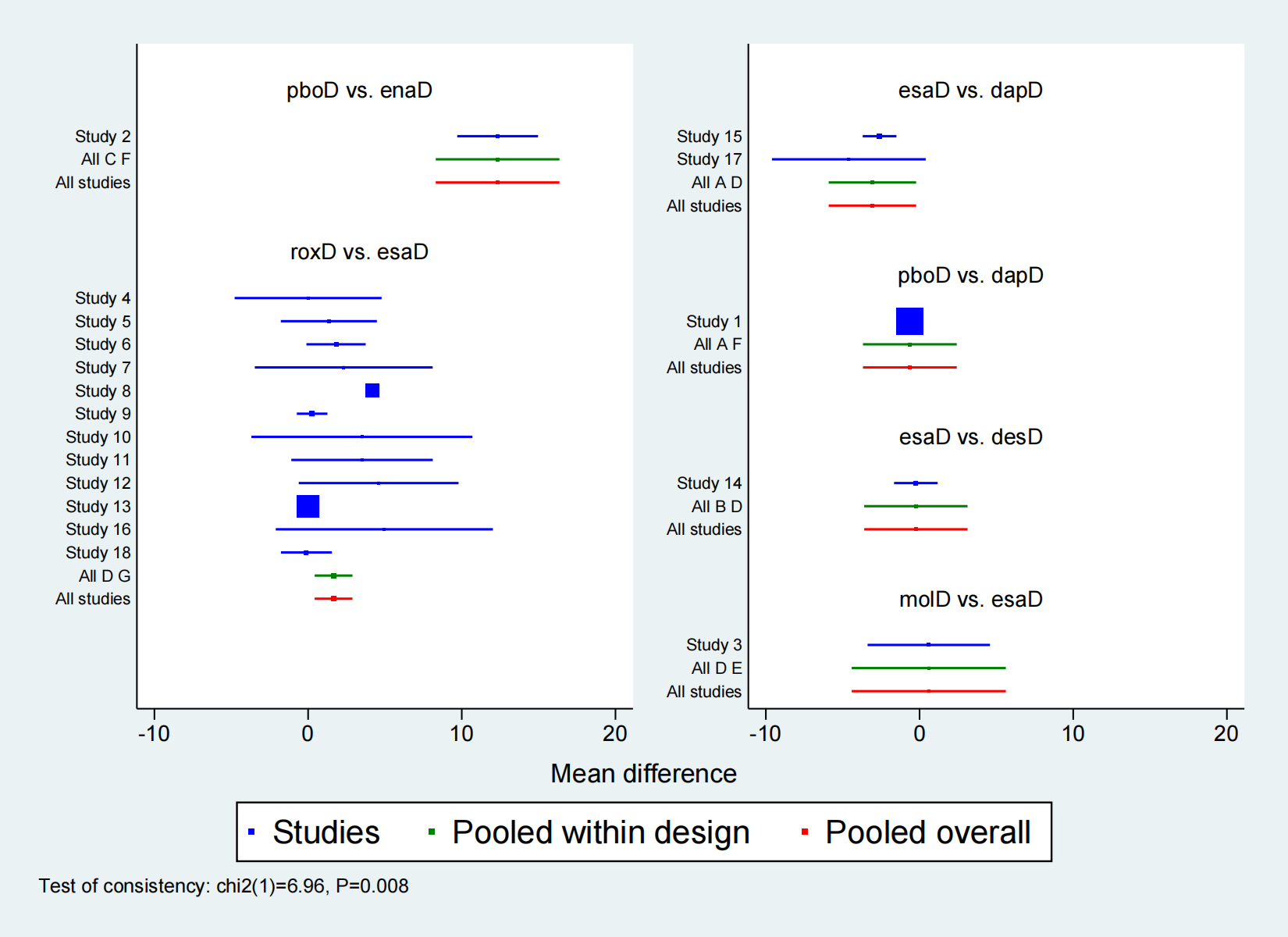


Figure 5.6. Forest plot of Serum Iron in dialysis patients (Consistency’s test: p = 0.008).


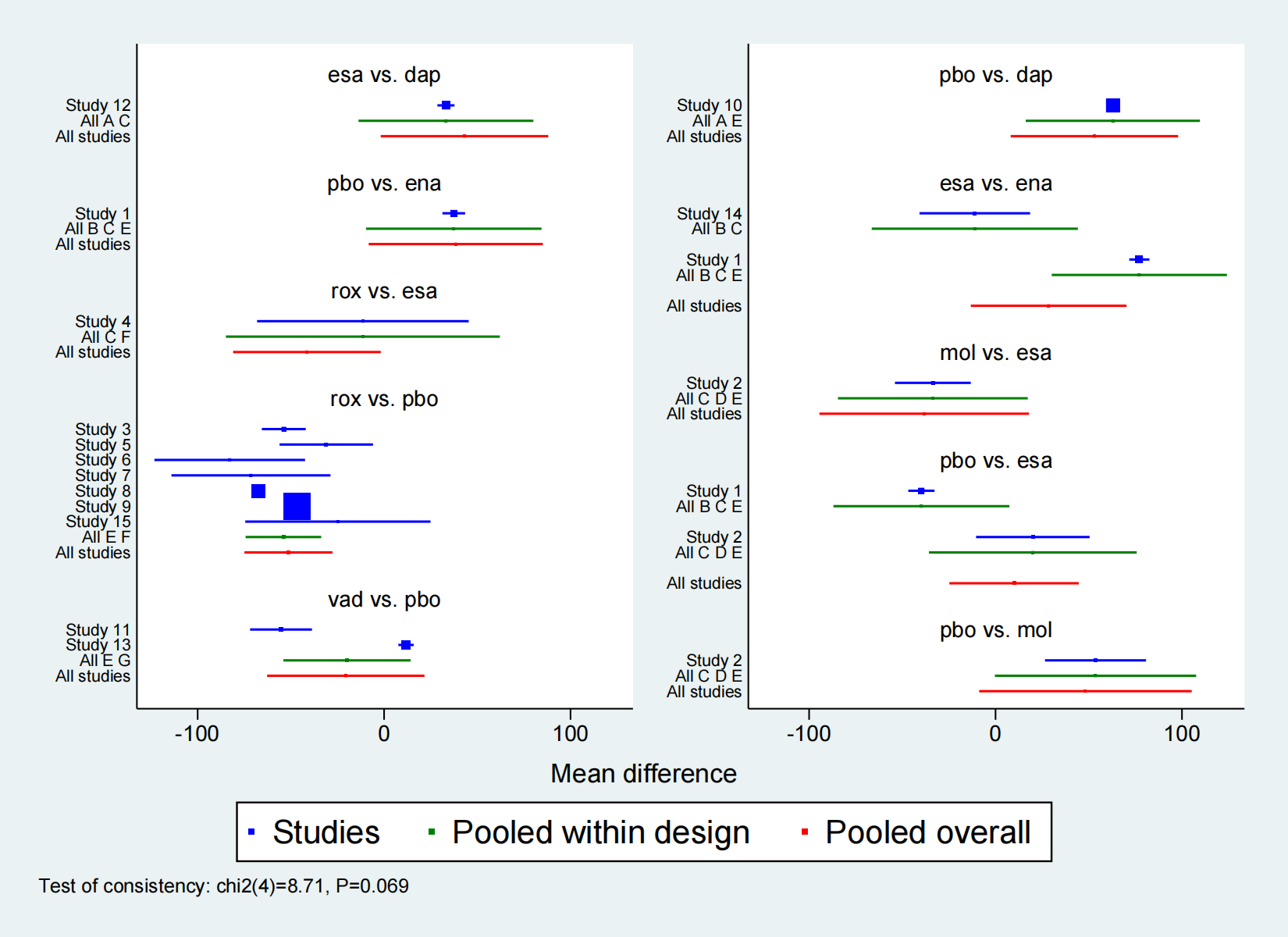


Figure 5.7. Forest plot of Ferritin in non-dialysis patients (Consistency’s test: p = 0.521).


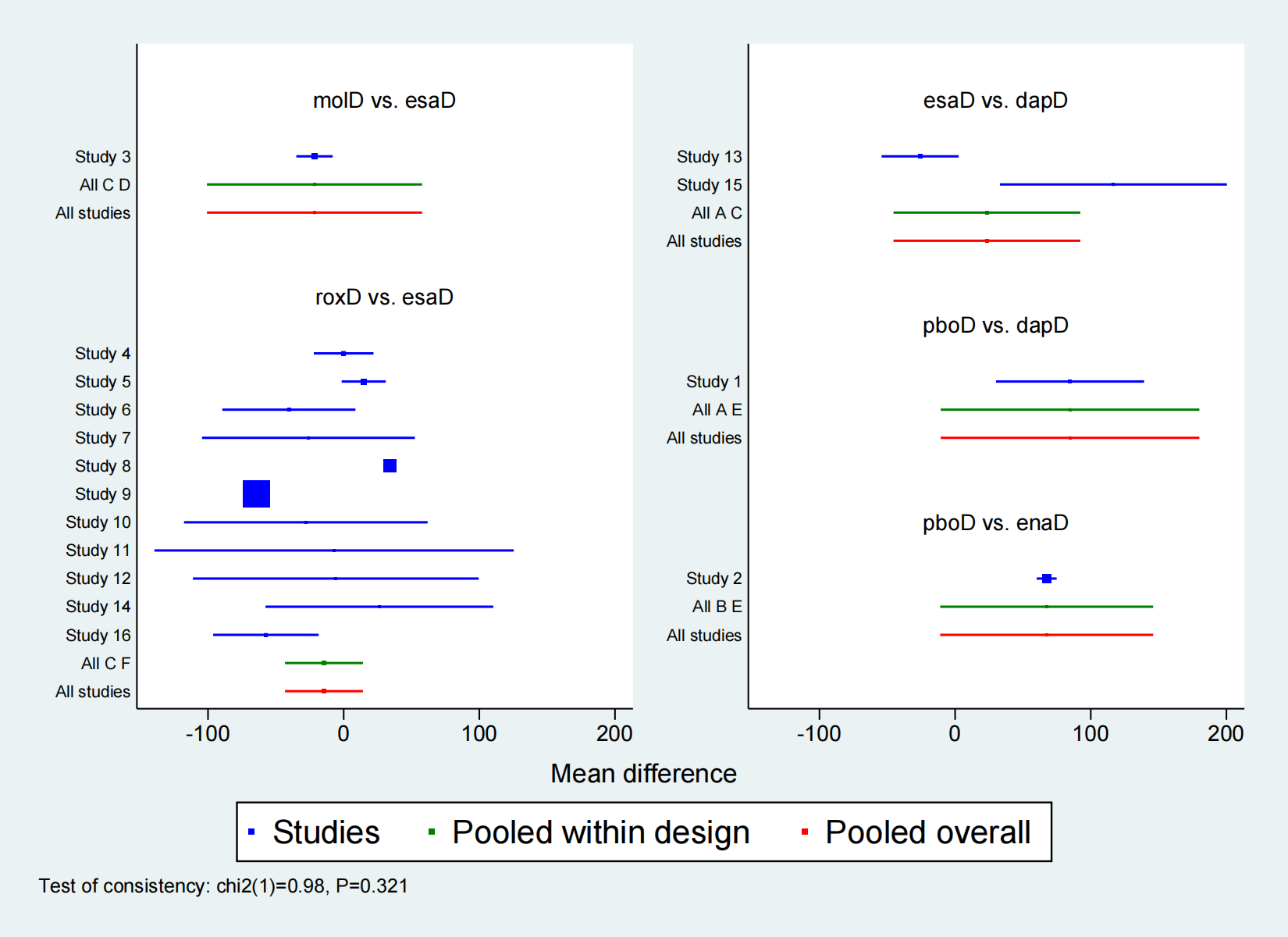


Figure 5.8. Forest plot of Ferritin in dialysis patients (Consistency’s test: p = 0.321).


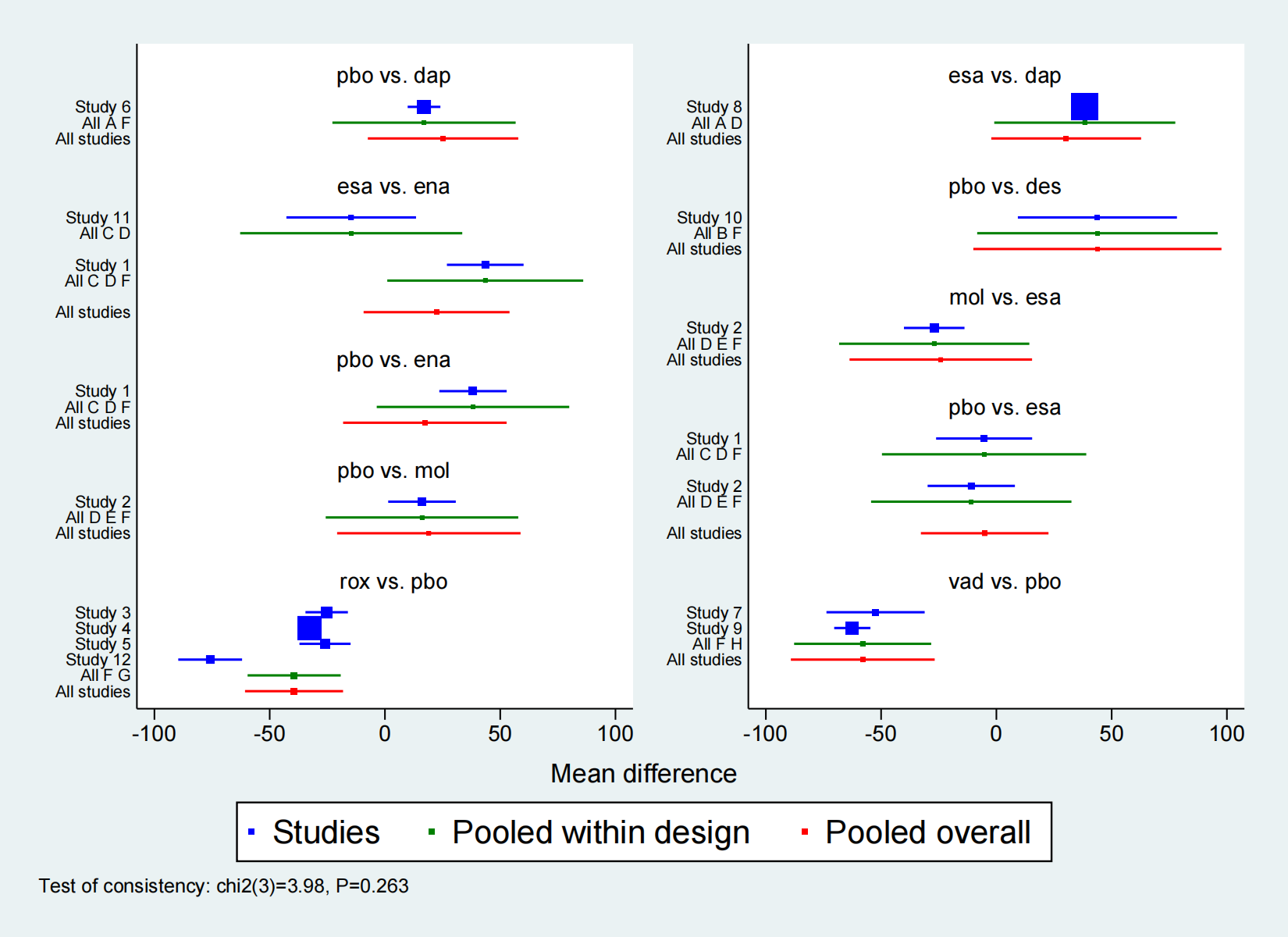


Figure 5.9. Forest plot of Hepcidin in non-dialysis patients (Consistency’s test: p = 0.263).


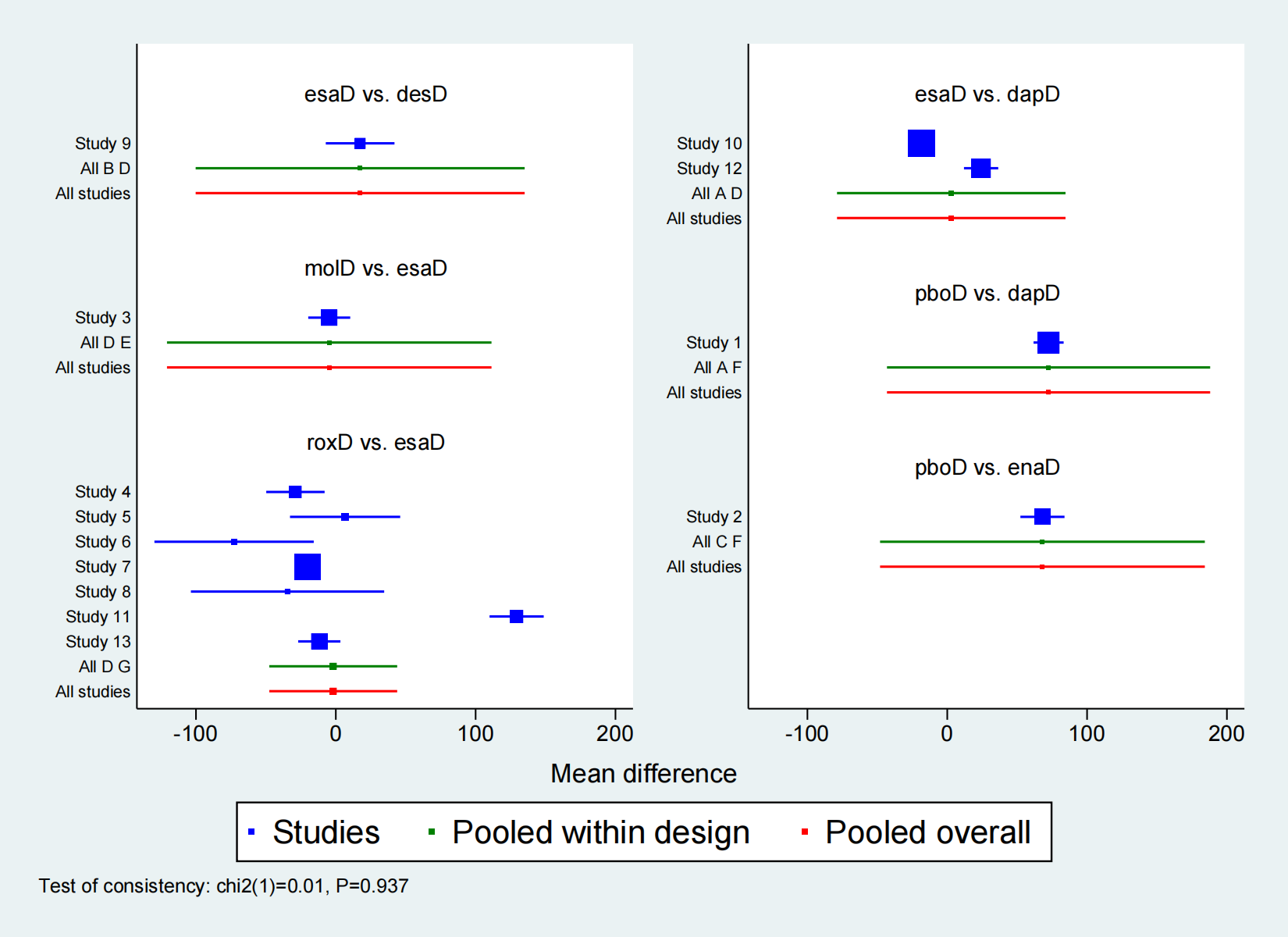


Figure 5.10. Forest plot of Hepcidin in dialysis patients (Consistency’s test: p = 0.937).


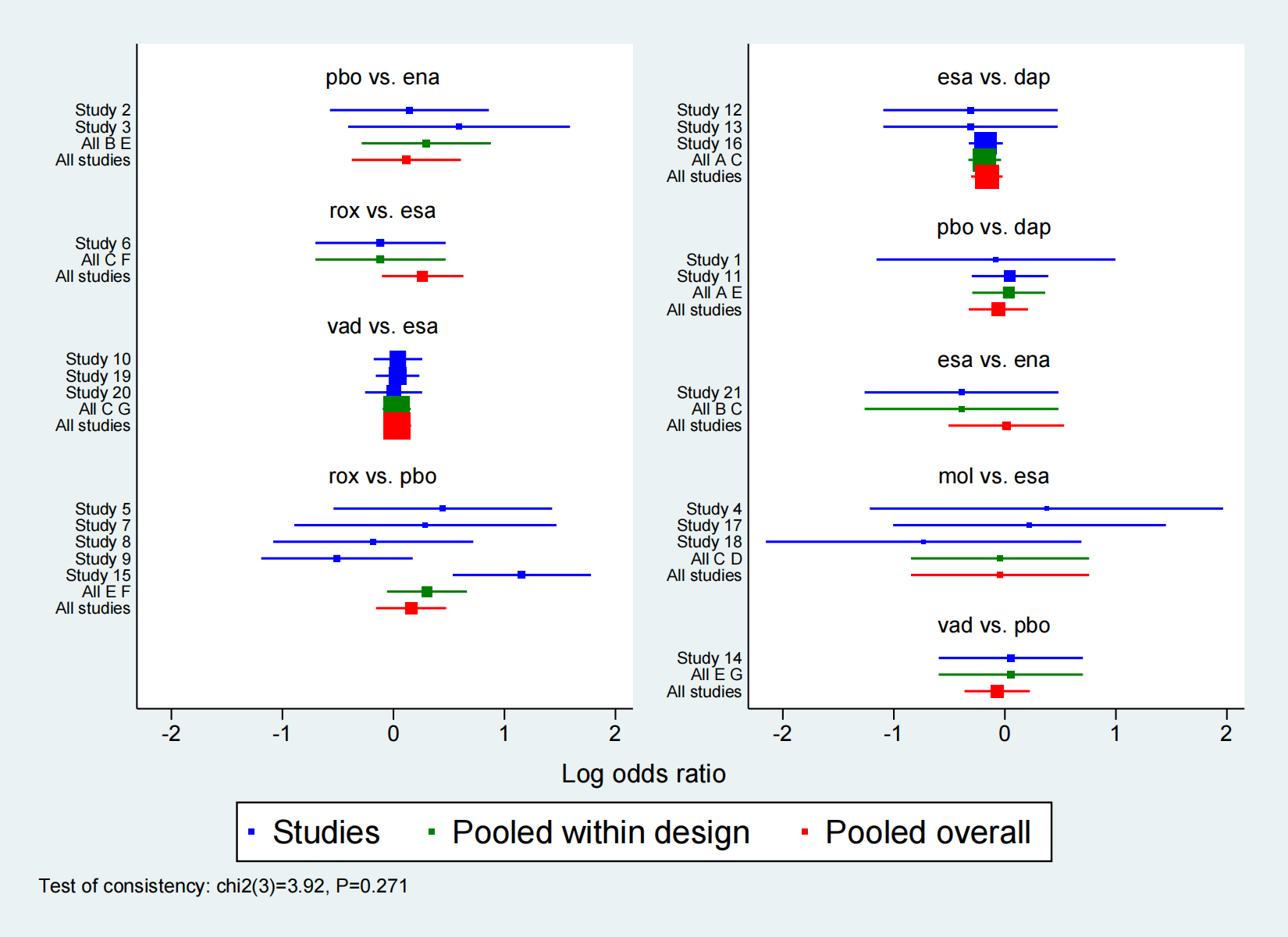


Figure 5.11. Forest plot of AE in non-dialysis patients (Consistency’s test: p = 0.271).


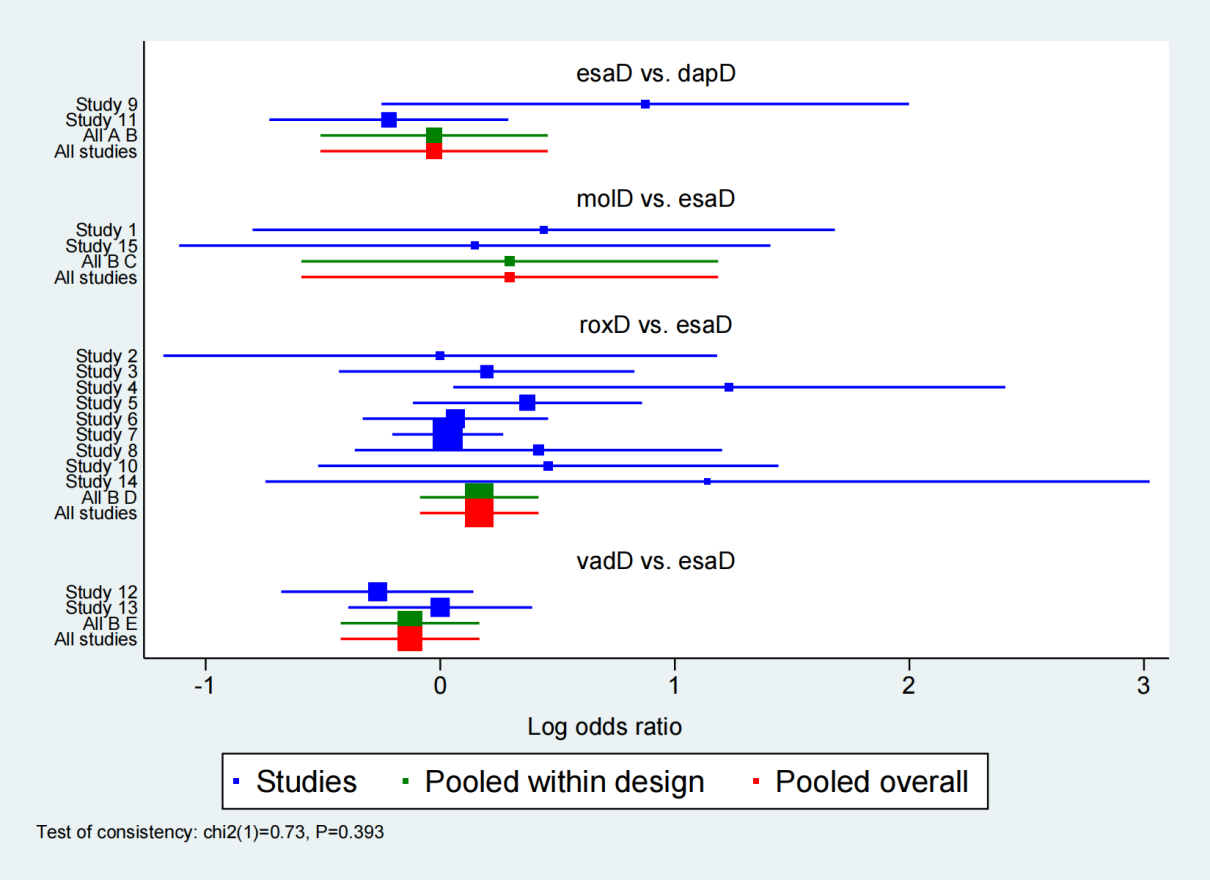


Figure 5.10. Forest plot of AE in dialysis patients (Consistency’s test: p = 0.393).

# Supplementary 6: Meta regression

Table 6.1 Hemoglobin in dialysis patients

| Covariate | Beta | Lower_95CrI | Upper_95CrI |
| --- | --- | --- | --- |
| Baseline TSAT | 0.06 | -0.02 | 0.14 |
| Dialysis frequency | -0.05 | -0.12 | 0.02 |
| Age | -0.03 | -0.1 | 0.05 |
| Publication year | 0.01 | -0.06 | 0.08 |
| Sample size | 0 | -0.05 | 0.06 |
| PHI starting dose | 0.02 | -0.06 | 0.1 |
| Male proportion | -0.01 | -0.08 | 0.06 |

# Supplementary 7: Node splitting method results

Table 7.1 Node splitting method results of hemoglobin in non-dialysis patients

| Side |  | Direct | | Indirect | | Difference | | P>\|z\| | tau |
| --- | --- | --- | --- | --- | --- | --- | --- | --- | --- |
|  |  | Coef. | Std. Err. | Coef. | Std. Err. | Coef. | Std. Err. |  |  |
| A | D | -0.0878576 | 0.4373595 | -0.8148828 | 0.5698609 | 0.7270252 | 0.7184117 | 0.312 | 0.6132728 |
| A | F | -1.35 | 0.6126684 | -0.2716629 | 0.4940092 | -1.078337 | 0.7870246 | 0.171 | 0.6098094 |
| B | D | -0.12 | 0.6232229 | -0.7207593 | 9.573967 | 0.6007593 | 9.594219 | 0.95 | 0.6230865 |
| C | D | -0.9979543 | 0.4321843 | 0.9944516 | 1.253904 | -1.992406 | 1.333543 | 0.135 | 0.5980384 |
| C | F | -1.064334 | 0.6615876 | -1.166633 | 0.693005 | 0.102299 | 0.9606996 | 0.915 | 0.6430906 |
| D | E | 0.3712733 | 0.2878463 | -2.506223 | 1.248014 | 2.877496 | 1.283282 | 0.025 | 0.5586434 |
| D | F | 0.9989029 | 0.2925119 | -1.114795 | 0.1987497 | 2.113698 | 0.3499145 | 0 | 0.332532 |
| D | G | -0.1000002 | 0.5793569 | 1.299814 | 0.3839798 | -1.399815 | 0.6950503 | 0.044 | 0.5717589 |
| D | H | 0.0233318 | 0.3687496 | 0.4528055 | 0.5640723 | -0.4294737 | 0.6739093 | 0.524 | 0.6386174 |
| E | F | 0.0412386 | 0.6752445 | -0.8669598 | 0.4896511 | 0.9081984 | 0.835091 | 0.277 | 0.6225972 |
| F | G | 1.396074 | 0.2476953 | -0.0036511 | 0.6496629 | 1.399725 | 0.694976 | 0.044 | 0.5717625 |
| F | H | 0.6570573 | 0.4644442 | 0.2895403 | 0.4918371 | 0.367517 | 0.6765642 | 0.587 | 0.6389071 |

Table 7.2 Node splitting method results of hemoglobin in dialysis patients

| Side |  | Direct | | Indirect | | Difference | | P>\|z\| | tau |
| --- | --- | --- | --- | --- | --- | --- | --- | --- | --- |
|  |  | Coef. | Std. Err. | Coef. | Std. Err. | Coef. | Std. Err. |  |  |
| A | D | -0.1044497 | 0.0807526 | -0.0300927 | 0.4590946 | -0.074357 | 0.4661368 | 0.873 | 0.1112421 |
| A | F | -1.72 | 0.2146463 | -1.126829 | 29.98387 | -0.5931707 | 29.98464 | 0.984 | 0.1107846 |
| B | D | -0.15 | 0.111179 | -0.2044844 | 5.697568 | 0.0544844 | 5.698652 | 0.992 | 0.1108067 |
| C | F | -1.54 | 0.2259485 | -2.726342 | 59.9809 | 1.186342 | 59.98136 | 0.984 | 0.1107844 |
| D | E | -0.1566894 | 0.1177426 | 0.2387526 | 35.82351 | -0.395442 | 35.8238 | 0.991 | 0.1107847 |
| D | G | 0.1626638 | 0.0514504 | 0.2037634 | 1.079609 | -0.0410996 | 1.080842 | 0.97 | 0.111299 |
| D | H | -0.2696686 | 0.0642616 | 0.2135848 | 1.898439 | -0.4832534 | 1.899527 | 0.799 | 0.1109316 |

Table 7.3 Node splitting method results of TSAT in dialysis patients

| Side |  | Direct | | Indirect | | Difference | | P>\|z\| | tau |
| --- | --- | --- | --- | --- | --- | --- | --- | --- | --- |
|  |  | Coef. | Std. Err. | Coef. | Std. Err. | Coef. | Std. Err. |  |  |
| A | D | -3.068461 | 1.452408 | -0.8491281 | 6.068858 | -2.219333 | 6.239369 | 0.722 | 1.555833 |
| A | F | -0.6300003 | 1.545823 | 9.021947 | 133.3772 | -9.651947 | 133.3861 | 0.942 | 1.544604 |
| B | D | -0.2400001 | 1.705292 | -5.894218 | 452.244 | 5.654218 | 452.2472 | 0.99 | 1.544579 |
| C | F | 12.33 | 2.045503 | -6.973895 | 266.7429 | 19.3039 | 266.7559 | 0.942 | 1.544606 |
| D | E | 0.6 | 2.552305 | 5.547594 | 721.4165 | -4.947594 | 721.4245 | 0.995 | 1.544575 |
| D | G | 1.657324 | 0.628388 | 6.0944 | 12.47052 | -4.437076 | 12.48465 | 0.722 | 1.555831 |

Table 7.4 Node splitting method results of Serum Iron in dialysis patients

| Side |  | Direct | | Indirect | | Difference | | P>\|z\| | tau |
| --- | --- | --- | --- | --- | --- | --- | --- | --- | --- |
|  |  | Coef. | Std. Err. | Coef. | Std. Err. | Coef. | Std. Err. |  |  |
| A | B | -5.418355 | 3.806659 | -3.166448 | 6.00035 | -2.251906 | 7.105905 | 0.751 | 5.218337 |
| A | D | 0.1626638 | 0.0514504 | 0.2037634 | 1.079609 | -0.0410996 | 1.080842 | 0.97 | 0.111299 |
| B | C | -2.6 | 6.831145 | 11.06109 | 1567.594 | -13.66109 | 1567.617 | 0.993 | 5.057392 |
| B | E | 6.1871 | 1.721253 | 10.68995 | 14.10702 | -4.50285 | 14.21207 | 0.751 | 5.218339 |

# Supplementary 8: Comparison of Deviance Information Criterion (DIC) between Bayesian fixed-effect and random-effects models

| **Outcome** | **Fixed-effect DIC** | **Random-effects DIC** | **Best-fit model** | **Interpretation** |
| --- | --- | --- | --- | --- |
| Hemoglobin (ND-CKD) | 8121.4 | 8114.2 | Random-effects | Heterogeneity across non-dialysis trials favored random-effects. |
| Hemoglobin (D-CKD) | 7325.6 | 7318.7 | Random-effects | Between-study variability in dialysis trials supported random-effects. |
| TSAT (ND-CKD) | 5120.9 | 5115.1 | Random-effects | Differences in assay methods and baseline iron status favored random-effects. |
| TSAT (D-CKD) | 4762.3 | 4756.5 | Random-effects | Better fit with random-effects reflecting dialysis-related iron loss variability. |
| Serum Iron (ND-CKD) | 3988.7 | 3982.9 | Random-effects | Random-effects captured heterogeneity across non-dialysis cohorts. |
| Serum Iron (D-CKD) | 3651.2 | 3645.8 | Random-effects | Random-effects provided superior fit in dialysis subgroup. |
| Ferritin (ND-CKD) | 4596.4 | 4590.3 | Random-effects | Variability in baseline ferritin and inflammation favored random-effects. |
| Ferritin (D-CKD) | 4335.8 | 4330.6 | Random-effects | Random-effects yielded lower DIC values in dialysis studies. |
| Hepcidin (ND-CKD) | 3880.2 | 3874.5 | Random-effects | Heterogeneity in measurement techniques supported random-effects. |
| Hepcidin (D-CKD) | 3549.7 | 3543.9 | Random-effects | Random-effects offered better model fit across dialysis trials. |
| Adverse Events (ND-CKD) | 6212.5 | 6213.8 | Fixed-effect | Minimal heterogeneity in AE reporting supported fixed-effect. |
| Adverse Events (D-CKD) | 5887.4 | 5888.6 | Fixed-effect | DIC values were similar; fixed-effect slightly better for parsimony. |

# Supplementary 9: Dataset Used for Network Meta-Analysis

Supplementary File 9.1: Dataset of Hemoglobin Outcomes in Non-Dialysis Patients.

| **id** | **t** | **mean** | **sd** | **n** |
| --- | --- | --- | --- | --- |
| 1.00 | pbo | -0.175 | 0.68 | 23 |
| 1.00 | ena | 0.86 | 0.78 | 150 |
| 1.00 | esa | -1.143 | 0.773 | 24 |
| 2.00 | mol | 1.26 | 0.94 | 193 |
| 2.00 | pbo | 1.4 | 1.14 | 20 |
| 2.00 | esa | 0.3 | 0.75 | 32 |
| 3.00 | mol | -0.3 | 0.79 | 118 |
| 3.00 | esa | -0.2 | 0.78 | 42 |
| 4.00 | pbo | 0.08 | 0.67 | 27 |
| 4.00 | rox | 1.18 | 1.05 | 80 |
| 5.00 | rox | 1.85 | 1.05 | 323 |
| 5.00 | esa | 1.95 | 1.25 | 293 |
| 6.00 | pbo | 0.37 | 0.87 | 30 |
| 6.00 | rox | 1.97 | 1.4 | 61 |
| 7.00 | pbo | 0.4 | 0.8 | 51 |
| 7.00 | rox | 1.9 | 1.2 | 101 |
| 8.00 | vad | 0.93 | 0.51 | 1741 |
| 8.00 | esa | 0.9 | 0.48 | 1735 |
| 9.00 | rox | 2 | 0.95 | 616 |
| 9.00 | pbo | 0.16 | 0.9 | 306 |
| 10.00 | dap | 1.59 | 0.79 | 307 |
| 10.00 | pbo | 0.24 | 0.67 | 307 |
| 11.00 | vad | 1.02 | 0.9 | 72 |
| 11.00 | pbo | -0.024 | 0.67 | 19 |
| 12.00 | dap | 1.51 | 0.79 | 108 |
| 12.00 | esa | 1.28 | 0.85 | 109 |
| 13.00 | vad | 0.27 | 0.90 | 138 |
| 13.00 | pbo | -0.02 | 0.67 | 72 |
| 14.00 | rox | 1.94 | 1.05 | 391 |
| 14.00 | pbo | 0.56 | 0.67 | 203 |
| 15.00 | dap | 0.51 | 0.79 | 1937 |
| 15.00 | esa | 0.56 | 0.85 | 1935 |
| 16.00 | mol | 1.82 | 0.85 | 82 |
| 16.00 | esa | 1.53 | 0.85 | 80 |
| 17.00 | mol | 0.65 | 0.85 | 82 |
| 17.00 | esa | 0.31 | 0.85 | 82 |
| 18 | vad | 1.43 | 0.046 | 879 |
| 18 | esa | 1.38 | 0.047 | 872 |
| 19 | vad | 0.41 | 0.036 | 862 |
| 19 | esa | 0.42 | 0.037 | 863 |
| 20 | des | 1.86 | 0.2 | 294 |
| 20 | esa | 1.74 | 0.1 | 294 |
| 21 | ena | -0.02 | 0.42 | 86 |
| 21 | esa | -0.08 | 0.31 | 86 |
| 22 | rox | 0.42 | 1.79 | 52 |
| 22 | pbo | -0.35 | 1.36 | 19 |

Supplementary File 9.2: Dataset of Hemoglobin Outcomes in Dialysis Patients.

| **id** | **t** | **mean** | **sd** | **n** |
| --- | --- | --- | --- | --- |
| 1.00 | pboD | -1.41 | 0.667 | 19 |
| 1.00 | dapD | 0.31 | 0.9 | 78 |
| 2.00 | pboD | -1.49 | 0.67 | 22 |
| 2.00 | enaD | 0.05 | 1.05 | 60 |
| 3.00 | molD | -0.3 | 0.95 | 157 |
| 3.00 | esaD | -0.2 | 0.62 | 42 |
| 4.00 | molD | -0.3 | 0.75 | 57 |
| 4.00 | esaD | 0.2 | 0.95 | 30 |
| 5.00 | roxD | 2.27 | 1.02 | 74 |
| 5.00 | esaD | 2.26 | 1.02 | 74 |
| 6.00 | roxD | -0.46 | 2.87 | 150 |
| 6.00 | esaD | -1.41 | 2.7 | 151 |
| 7.00 | roxD | 0.39 | 0.93 | 370 |
| 7.00 | esaD | 0.09 | 0.84 | 371 |
| 8.00 | esaD | 0.17 | 0.96 | 22 |
| 8.00 | roxD | 0.87 | 1.17 | 74 |
| 9.00 | roxD | 0.7 | 1.1 | 204 |
| 9.00 | esaD | 0.5 | 1 | 100 |
| 10.00 | roxD | 0.397 | 0.773 | 414 |
| 10.00 | esaD | 0.183 | 0.86 | 420 |
| 11.00 | roxD | -0.5 | 0.2 | 108 |
| 11.00 | esaD | -0.5 | 0.3 | 36 |
| 12.00 | dapD | 1.24 | 0.79 | 157 |
| 12.00 | esaD | 1.09 | 0.85 | 155 |
| 13.00 | roxD | 0.024 | 0.015 | 106 |
| 13.00 | esaD | 0.02 | 0.02 | 53 |
| 14 | vadD | 1.26 | 0.109 | 181 |
| 14 | esaD | 1.58 | 0.108 | 188 |
| 15 | vadD | 0.19 | 0.032 | 1777 |
| 15 | esaD | 0.36 | 0.032 | 1777 |
| 16 | vadD | 0.04 | 0.14 | 304 |
| 16 | esaD | 0.36 | 0.092 | 152 |
| 17 | desD | 0.95 | 0.09 | 196 |
| 17 | esaD | 0.8 | 0.09 | 196 |
| 18 | molD | 0.29 | 1.9 | 153 |
| 18 | esaD | 0.17 | 1.4 | 76 |
| 19 | dapD | 0.04 | 0.22 | 270 |
| 19 | esaD | -0.05 | 0.37 | 137 |
| 20 | roxD | -0.68 | 2.04 | 60 |
| 20 | esaD | -0.31 | 1.66 | 20 |
| 21 | dapD | 0.08 | 0.37 | 171 |
| 21 | esaD | 0.01 | 0.84 | 39 |
| 22 | roxD | 2.57 | 1.27 | 522 |
| 22 | esaD | 2.36 | 1.21 | 521 |

Supplementary File 9.3: Dataset of TSAT Outcomes in Non-Dialysis Patients.

| **id** | **t** | **mean** | **sd** | **n** |
| --- | --- | --- | --- | --- |
| 1 | pbo | -3.75 | 6.3 | 23 |
| 1 | ena | -6.37 | 8.68 | 150 |
| 1 | esa | -2.02 | 5.3 | 24 |
| 2 | mol | -4.25 | 12.45 | 193 |
| 2 | pbo | -5.6 | 10.5 | 20 |
| 2 | esa | 0.2 | 11.6 | 32 |
| 3 | pbo | 0.5 | 7.8 | 27 |
| 3 | rox | -7.94 | 13.88 | 80 |
| 4 | rox | 1.6 | 12 | 323 |
| 4 | esa | 3.9 | 11.5 | 293 |
| 5 | pbo | -3.1 | 7.8 | 18 |
| 5 | rox | -8.1 | 9.3 | 67 |
| 6 | pbo | 0.24 | 7.92 | 30 |
| 6 | rox | -6.29 | 9.79 | 61 |
| 7 | pbo | -1.7 | 9.2 | 51 |
| 7 | rox | -5.2 | 10.4 | 101 |
| 8 | rox | 1.05 | 13.6 | 616 |
| 8 | pbo | 0.38 | 7.8 | 306 |
| 9 | dap | 0 | 0.38 | 307 |
| 9 | pbo | 0.21 | 0.36 | 307 |
| 10 | dap | -3.14 | 11.5 | 108 |
| 10 | esa | 2.71 | 11.5 | 109 |
| 11 | des | 0.81 | 9.7 | 294 |
| 11 | esa | 5.01 | 11.9 | 294 |
| 12 | ena | 5.01 | 19.99 | 86 |
| 12 | esa | 2.16 | 16.87 | 86 |
| 13 | rox | -3.03 | 7.91 | 52 |
| 13 | pbo | 1.5 | 6.1 | 19 |

Supplementary File 9.4: Dataset of TSAT Outcomes in Dialysis Patients.

| **id** | **t** | **mean** | **sd** | **n** |
| --- | --- | --- | --- | --- |
| 1 | pboD | 0 | 0.2 | 19 |
| 1 | dapD | 0.63 | 0.36 | 78 |
| 2 | pboD | 7.84 | 5.9 | 22 |
| 2 | enaD | -4.49 | 3.6 | 60 |
| 3 | molD | 1.2 | 11.3 | 157 |
| 3 | esaD | 0.6 | 11.8 | 42 |
| 4 | roxD | -5.02 | 14.84 | 74 |
| 4 | esaD | -5.02 | 14.84 | 74 |
| 5 | roxD | -1.09 | 13.84 | 150 |
| 5 | esaD | -2.44 | 13.83 | 151 |
| 6 | roxD | -7.96 | 13.7 | 370 |
| 6 | esaD | -9.78 | 13.07 | 371 |
| 7 | esaD | -8.29 | 10.46 | 22 |
| 7 | roxD | -5.97 | 17.95 | 86 |
| 8 | roxD | -4.5 | 1.2 | 204 |
| 8 | esaD | -8.7 | 1 | 100 |
| 9 | roxD | -2.6 | 11.5 | 1051 |
| 9 | esaD | -2.86 | 11.8 | 1055 |
| 10 | roxD | -0.7 | 20.4 | 78 |
| 10 | esaD | -4.2 | 17.8 | 39 |
| 11 | roxD | -2.4 | 16.8 | 108 |
| 11 | esaD | -5.91 | 10.23 | 36 |
| 12 | roxD | -3.26 | 15.57 | 106 |
| 12 | esaD | -7.85 | 15.87 | 53 |
| 13 | roxD | -0.02 | 0.18 | 13 |
| 13 | esaD | -0.03 | 0.2 | 13 |
| 14 | desD | 3.36 | 7.4 | 196 |
| 14 | esaD | 3.12 | 6.9 | 196 |
| 15 | dapD | -1.57 | 3.86 | 270 |
| 15 | esaD | -4.17 | 5.95 | 137 |
| 16 | roxD | 6.16 | 16.52 | 60 |
| 16 | esaD | 1.2 | 13 | 20 |
| 17 | dapD | -4.4 | 6.9 | 171 |
| 17 | esaD | -9 | 15.6 | 39 |
| 18 | roxD | -1.9 | 13.79 | 522 |
| 18 | esaD | -1.79 | 13.52 | 521 |

Supplementary File 9.5: Dataset of Serum Iron Outcomes in Non-Dialysis Patients.

| **id** | **t** | **mean** | **sd** | **n** |
| --- | --- | --- | --- | --- |
| 1 | mol | -11.27 | 39.06 | 193 |
| 1 | pbo | -14.2 | 25.9 | 20 |
| 1 | esa | 3.7 | 32 | 32 |
| 2 | rox | 1.93 | 6.96 | 323 |
| 2 | esa | 1.71 | 5.09 | 293 |
| 3 | pbo | -9.5 | 19.3 | 18 |
| 3 | rox | -11 | 23.3 | 67 |
| 4 | pbo | 2.7 | 23.7 | 30 |
| 4 | rox | -4.02 | 26.56 | 61 |
| 5 | pbo | -0.24 | 6.31 | 51 |
| 5 | rox | -0.64 | 4.36 | 101 |
| 6 | rox | 12.6 | 5.6 | 616 |
| 6 | pbo | 3.05 | 4.8 | 306 |
| 7 | rox | 7.84 | 3.6 | 1384 |
| 7 | pbo | 0.98 | 1.9 | 1377 |
| 8 | dap | 1.68 | 5.8 | 307 |
| 8 | pbo | -0.35 | 12.8 | 307 |
| 9 | dap | 3.23 | 5.8 | 108 |
| 9 | esa | 8.73 | 27 | 109 |
| 10 | des | 3.13 | 7.8 | 294 |
| 10 | esa | 5.89 | 11.4 | 294 |
| 11 | des | 3.34 | 43.81 | 86 |
| 11 | pbo | 5.24 | 26.91 | 30 |
| 12 | ena | 18.6 | 52.86 | 86 |
| 12 | esa | 7.95 | 35.34 | 86 |
| 13 | rox | -0.8 | 4.43 | 52 |
| 13 | pbo | 0.6 | 2.8 | 19 |

Supplementary File 9.6: Dataset of Serum Iron Outcomes in Dialysis Patients.

| **id** | **t** | **mean** | **sd** | **n** |
| --- | --- | --- | --- | --- |
| 1 | pboD | 0 | 4.46 | 19 |
| 1 | dapD | -3.83 | 5.86 | 78 |
| 2 | molD | 4.9 | 24.2 | 157 |
| 2 | esaD | 7.5 | 27 | 42 |
| 3 | roxD | 0.4 | 6.62 | 74 |
| 3 | esaD | 0.4 | 6.6 | 74 |
| 4 | roxD | 1.2 | 6.4 | 150 |
| 4 | esaD | -0.9 | 5.5 | 151 |
| 5 | roxD | -1.07 | 1.6 | 370 |
| 5 | esaD | -15.5 | 11.1 | 371 |
| 6 | esaD | -18.9 | 26.7 | 22 |
| 6 | roxD | 3.02 | 42.98 | 74 |
| 7 | roxD | 0.6 | 0.7 | 204 |
| 7 | esaD | -3.9 | 0.5 | 100 |
| 8 | roxD | 4 | 2.6 | 1051 |
| 8 | esaD | -6.6 | 2.3 | 1055 |
| 9 | roxD | 0.8 | 5.9 | 78 |
| 9 | esaD | -1.4 | 4.2 | 39 |
| 10 | roxD | 5.92 | 39.1 | 108 |
| 10 | esaD | -8.57 | 25.15 | 36 |
| 11 | roxD | 0.21 | 0.16 | 13 |
| 11 | esaD | -0.22 | 0.11 | 12 |
| 12 | dapD | 4 | 9.14 | 270 |
| 12 | esaD | -5.15 | 14.3 | 137 |
| 13 | roxD | 4.35 | 7.41 | 60 |
| 13 | esaD | 0.4 | 5.7 | 20 |
| 14 | dapD | 0.9 | 6.1 | 171 |
| 14 | esaD | -0.8 | 7.6 | 39 |
| 15 | roxD | 4.31 | 37.22 | 522 |
| 15 | esaD | -4.06 | 34.9 | 521 |

Supplementary File 9.7: Dataset of Ferritin Outcomes in Non-Dialysis Patients.

| **id** | **t** | **mean** | **sd** | **n** |
| --- | --- | --- | --- | --- |
| 1 | pbo | -11.36 | 13.1 | 23 |
| 1 | ena | -48.72 | 18.07 | 150 |
| 1 | esa | 28.4 | 11.4 | 24 |
| 2 | mol | -58.72 | 74.65 | 193 |
| 2 | pbo | -5.1 | 57 | 20 |
| 2 | esa | -25.1 | 50.2 | 32 |
| 3 | pbo | -10.3 | 23.2 | 27 |
| 3 | rox | -64.1 | 35.92 | 80 |
| 4 | rox | -131.8 | 355.46 | 323 |
| 4 | esa | -120.42 | 362.08 | 293 |
| 5 | pbo | -37.8 | 40.3 | 18 |
| 5 | rox | -68.8 | 70.1 | 67 |
| 6 | pbo | -28 | 64 | 30 |
| 6 | rox | -110.79 | 132.61 | 61 |
| 7 | pbo | -21.9 | 115.5 | 51 |
| 7 | rox | -93.3 | 146.3 | 101 |
| 8 | rox | -59.62 | 21.7 | 616 |
| 8 | pbo | 7.69 | 15.3 | 306 |
| 9 | rox | -38.4 | 16.9 | 1384 |
| 9 | pbo | 8.1 | 11.8 | 1377 |
| 10 | dap | -65.85 | 17.5 | 307 |
| 10 | pbo | -2.93 | 11.3 | 307 |
| 11 | vad | -75.14 | 36.8 | 72 |
| 11 | pbo | -19.86 | 31.7 | 19 |
| 12 | dap | -53.3 | 17.9 | 108 |
| 12 | esa | -20.12 | 16.5 | 109 |
| 13 | vad | -19.5 | 13.5 | 138 |
| 13 | pbo | -31.25 | 14.9 | 72 |
| 14 | ena | 49.89 | 115.19 | 86 |
| 14 | esa | 38.76 | 80.11 | 86 |
| 15 | rox | -49.09 | 171.34 | 52 |
| 15 | pbo | -24.3 | 38.6 | 19 |

Supplementary File 9.8: Dataset of Ferritin Outcomes in Dialysis Patients.

| **id** | **t** | **mean** | **sd** | **n** |
| --- | --- | --- | --- | --- |
| 1 | pboD | 4.7 | 114.79 | 19 |
| 1 | dapD | -80.01 | 80.45 | 78 |
| 2 | pboD | 55.22 | 15.7 | 22 |
| 2 | enaD | -12.27 | 13.16 | 60 |
| 3 | molD | 26.5 | 36.8 | 157 |
| 3 | esaD | 48 | 40 | 42 |
| 4 | roxD | -74.35 | 68.28 | 74 |
| 4 | esaD | -74.34 | 68.28 | 74 |
| 5 | roxD | -3.98 | 78.41 | 150 |
| 5 | esaD | -18.75 | 64.64 | 151 |
| 6 | roxD | -429.91 | 340.16 | 370 |
| 6 | esaD | -389.52 | 341.11 | 371 |
| 7 | esaD | -70 | 157 | 22 |
| 7 | roxD | -95.96 | 188.77 | 74 |
| 8 | roxD | -99 | 19 | 204 |
| 8 | esaD | -133 | 21 | 100 |
| 9 | roxD | -127.4 | 21.9 | 1051 |
| 9 | esaD | -63.2 | 25.7 | 1055 |
| 10 | roxD | -55.5 | 299.5 | 78 |
| 10 | esaD | -27.6 | 192.3 | 39 |
| 11 | roxD | -195.18 | 287.4 | 108 |
| 11 | esaD | -188.09 | 369.85 | 36 |
| 12 | roxD | -49.35 | 175.3 | 13 |
| 12 | esaD | -43.54 | 79.34 | 12 |
| 13 | dapD | -194.2 | 98.75 | 270 |
| 13 | esaD | -220 | 154.37 | 137 |
| 14 | roxD | -1.55 | 165.6 | 60 |
| 14 | esaD | -27.9 | 166 | 20 |
| 15 | dapD | -59.9 | 331.8 | 171 |
| 15 | esaD | 56.8 | 214.3 | 39 |
| 16 | roxD | -198.47 | 311.7 | 522 |
| 16 | esaD | -141.13 | 328.52 | 521 |

Supplementary File 9.9: Dataset of Hepcidin Outcomes in Non-Dialysis Patients.

| **id** | **t** | **mean** | **sd** | **n** |
| --- | --- | --- | --- | --- |
| 1 | pbo | 0.77 | 33.3 | 23 |
| 1 | ena | -37.42 | 33.12 | 150 |
| 1 | esa | 6.09 | 39.4 | 24 |
| 2 | mol | -13.07 | 26.76 | 193 |
| 2 | pbo | 3 | 32.3 | 20 |
| 2 | esa | 13.9 | 36.32 | 32 |
| 3 | pbo | -3.9 | 19.9 | 27 |
| 3 | rox | -29.18 | 24.35 | 80 |
| 4 | pbo | -4.8 | 8.17 | 30 |
| 4 | rox | -37.5 | 9.53 | 61 |
| 5 | rox | -22.11 | 80.9 | 616 |
| 5 | pbo | 3.88 | 80.9 | 306 |
| 6 | dap | -25.85 | 16.8 | 307 |
| 6 | pbo | -8.9 | 61.2 | 307 |
| 7 | vad | -86.45 | 64 | 72 |
| 7 | pbo | -34.04 | 34.1 | 19 |
| 8 | dap | -27.93 | 11.9 | 108 |
| 8 | esa | 10.41 | 13.5 | 109 |
| 9 | vad | -72.5 | 36.4 | 138 |
| 9 | pbo | -10 | 21.6 | 72 |
| 10 | des | -81.49 | 94.39 | 86 |
| 10 | pbo | -37.67 | 78.67 | 30 |
| 11 | ena | 47.7 | 98.92 | 86 |
| 11 | esa | 33.07 | 88.97 | 86 |
| 12 | rox | -83.11 | 32.1 | 52 |
| 12 | pbo | -7.3 | 23.9 | 19 |

Supplementary File 9.10: Dataset of Hepcidin Outcomes in Dialysis Patients.

| **id** | **t** | **mean** | **sd** | **n** |
| --- | --- | --- | --- | --- |
| 1 | pboD | 3.1 | 22.3 | 19 |
| 1 | dapD | -69.36 | 17.52 | 78 |
| 2 | pboD | 43.28 | 34.1 | 22 |
| 2 | enaD | -24.8 | 26.7 | 60 |
| 3 | molD | 0.6 | 45.7 | 157 |
| 3 | esaD | 5.3 | 43.7 | 42 |
| 4 | roxD | -95.53 | 148.27 | 370 |
| 4 | esaD | -66.66 | 141.61 | 371 |
| 5 | esaD | -77.9 | 75.18 | 22 |
| 5 | roxD | -71.27 | 104.41 | 74 |
| 6 | roxD | -52.35 | 202.75 | 108 |
| 6 | esaD | 20.4 | 129.31 | 36 |
| 7 | roxD | -24.77 | 22.77 | 106 |
| 7 | esaD | -4.68 | 28.91 | 53 |
| 8 | roxD | -78.77 | 87.93 | 13 |
| 8 | esaD | -44.24 | 88.23 | 12 |
| 9 | desD | -36.6 | 94.04 | 196 |
| 9 | esaD | -19.2 | 148.1 | 196 |
| 10 | dapD | -34.27 | 32.1 | 270 |
| 10 | esaD | -52.61 | 46.14 | 137 |
| 11 | roxD | 88.31 | 45.6 | 60 |
| 11 | esaD | -41 | 35.5 | 20 |
| 12 | dapD | -20.6 | 36.7 | 171 |
| 12 | esaD | 3.6 | 34.9 | 39 |
| 13 | roxD | -67.78 | 112.71 | 522 |
| 13 | esaD | -55.96 | 135.32 | 521 |

Supplementary File 9.11: Dataset of AE Outcomes in Non-Dialysis Patients.

| **id** | **t** | **r** | **n** |
| --- | --- | --- | --- |
| 1 | pbo | 6 | 19 |
| 1 | dap | 26 | 78 |
| 2 | pbo | 13 | 47 |
| 2 | ena | 49 | 197 |
| 3 | pbo | 14 | 22.00 |
| 3 | ena | 31 | 63.00 |
| 4 | mol | 8 | 118 |
| 4 | esa | 2 | 42 |
| 5 | pbo | 19 | 27 |
| 5 | rox | 63 | 80 |
| 6 | rox | 296 | 323 |
| 6 | esa | 271 | 293 |
| 7 | pbo | 13 | 18 |
| 7 | rox | 52 | 67 |
| 8 | pbo | 19 | 30 |
| 8 | rox | 36 | 61 |
| 9 | pbo | 25 | 51 |
| 9 | rox | 37 | 101 |
| 10 | vad | 1565 | 1741 |
| 10 | esa | 1553 | 1735 |
| 11 | dap | 213 | 307 |
| 11 | pbo | 216 | 307 |
| 12 | dap | 137 | 149 |
| 12 | esa | 134 | 150 |
| 13 | dap | 137 | 149 |
| 13 | esa | 134 | 150 |
| 14 | vad | 103 | 138 |
| 14 | pbo | 53 | 72 |
| 15 | rox | 373 | 391 |
| 15 | pbo | 176 | 203 |
| 16 | dap | 1545 | 1937 |
| 16 | esa | 1487 | 1935 |
| 17 | mol | 77 | 82 |
| 17 | esa | 74 | 80 |
| 18 | mol | 76 | 82 |
| 18 | esa | 79 | 82 |
| 19 | vad | 573 | 879 |
| 19 | esa | 561 | 872 |
| 20 | vad | 139 | 862 |
| 20 | esa | 139 | 863 |
| 21 | ena | 76 | 86 |
| 21 | esa | 72 | 86 |

Supplementary File 9.12: Dataset of AE Outcomes in Dialysis Patients.

| **id** | **t** | **r** | **n** |
| --- | --- | --- | --- |
| 1 | molD | 11 | 57 |
| 1 | esaD | 4 | 30 |
| 2 | roxD | 68 | 74 |
| 2 | esaD | 68 | 74 |
| 3 | roxD | 129 | 150 |
| 3 | esaD | 126 | 151 |
| 4 | esaD | 4 | 22 |
| 4 | roxD | 32 | 74 |
| 5 | roxD | 96 | 204 |
| 5 | esaD | 38 | 100 |
| 6 | roxD | 359 | 414 |
| 6 | esaD | 361 | 420 |
| 7 | roxD | 891 | 1051 |
| 7 | esaD | 890 | 1055 |
| 8 | roxD | 38 | 78 |
| 8 | esaD | 15 | 39 |
| 9 | dapD | 205 | 220 |
| 9 | esaD | 131 | 135 |
| 10 | roxD | 26 | 108 |
| 10 | esaD | 6 | 36 |
| 11 | dapD | 120 | 157 |
| 11 | esaD | 112 | 155 |
| 12 | vadD | 89 | 181 |
| 12 | esaD | 105 | 188 |
| 13 | vadD | 134 | 304 |
| 13 | esaD | 67 | 152 |
| 14 | roxD | 5 | 13 |
| 14 | esaD | 2 | 12 |
| 15 | molD | 146 | 153 |
| 15 | esaD | 72 | 76 |

# Supplementary 10: Stata Code for Network Meta-Analysis

* 1) Data structure (example)

*-----------------------------

* Required variables (one row per study arm):

* id : study identifier

* t : treatment code (string or numeric; see labels below)

* mean : mean of the continuous outcome

* sd : standard deviation of the continuous outcome

* n : sample size per arm

*

* Example label mapping (edit as appropriate):

* A=rox, B=dap, C=mol, D=des, E=ena, F=vad, P=placebo/standard care

*

* Make sure the working dataset in memory contains the required variables.

*-----------------------------

* 2) Network setup (continuous outcomes; SMD scale)

*-----------------------------

* The "format(augment)" option augments the network to improve connectivity.

network setup mean sd n, study(id) trt(t) format(augment) smd

* Visualize network geometry

network map, lab(size(small))

*-----------------------------

* 3) Frequentist NMA: random- and common-effects

*-----------------------------

* Random-effects

network meta i

* Common-effects (fixed-effect analogue)

network meta c

* Forest plots of NMA estimates

network forest, sort(trt) esref

*-----------------------------

* 4) Treatment ranking (probabilities & SUCRA)

*-----------------------------

* For beneficial outcomes (larger-is-better):

network rank max, all zero reps(5000) gen(prob)

sucra prob*, lab(A B C D E F) // edit labels as needed

* If the outcome is harmful (smaller-is-better), use instead:

* network rank min, all zero reps(5000) gen(prob)

*-----------------------------

* 5) League table

*-----------------------------

netleague, lab(A B C D E F) sort(D C E B A F)

*-----------------------------

* 6) Small-study effects / publication bias (pairwise scale)

*-----------------------------

* Convert to pairwise comparisons

network convert pairs

* Egger's test (meta-regression of effect on its SE)

metabias _y _stderr, egger graph

* Comparison-adjusted funnel plot (random-effects)

netfunnel _y _stderr _t1 _t2, random bycomp add(lfit _stderr _ES_CEN) noalpha

*-----------------------------

* 7) Consistency / Inconsistency assessment

*-----------------------------

* 7a) Global inconsistency (design-by-treatment interaction)

* (Syntax may vary by Stata minor version; consult "help network meta" for options)

capture noisily network meta inconsistency

* 7b) Local inconsistency (node-splitting; all eligible comparisons)

* The "tau" option estimates heterogeneity separately per split.

network sidesplit all, tau

* Interpretation guidance (for reporting):

* - Global test p-value > 0.05: no evidence of inconsistency at the network level.

* - Node-splitting p-value < 0.05: evidence of local inconsistency for that split.

*-----------------------------

* 8) OPTIONAL: Save outputs

*-----------------------------

* graph export "network_map.png", replace

* graph export "network_forest.png", replace

* estimates save "nma_random_estimates.ster", replace

* estimates save "nma_common_estimates.ster", replace
